# APEX1 Nuclease and Redox Functions are Both Essential for Adult Mouse Hematopoietic Stem and Progenitor Cells

**DOI:** 10.1007/s12015-023-10550-0

**Published:** 2023-06-02

**Authors:** Samantha Zaunz, Jonathan De Smedt, Lukas Lauwereins, Lana Cleuren, Charlie Laffeber, Manmohan Bajaj, Joyce H. G. Lebbink, Jurgen A. Marteijn, Kim De Keersmaecker, Catherine Verfaillie

**Affiliations:** 1 Stem Cell Institute, Department of Development and Regeneration, KU Leuven, O&N IV Herestraat 49, 3000 Louvain, Belgium; 2 Present Address: GlaxoSmithKline Biologicals SA, 1300 Wavre, Belgium; 3 Department of Molecular Genetics, Oncode Institute, Erasmus MC Cancer Institute, Erasmus University Medical Center, Rotterdam, Netherlands; 4 Department of Radiotherapy, Erasmus MC Cancer Institute, Erasmus University Medical Center, Rotterdam, The Netherlands; 5 Laboratory for Disease Mechanisms in Cancer, Department of Oncology, KU Leuven, Louvain, Belgium

**Keywords:** APEX1, Mouse Hematopoietic Stem and Progenitor, Expansion, Endonuclease, REF-1

## Abstract

Self-renewal and differentiation of hematopoietic stem and progenitor cells (HSPCs) are carefully controlled by extrinsic and intrinsic factors, to ensure the lifelong process of hematopoiesis. Apurinic/apyrimidinic endonuclease 1 (APEX1) is a multifunctional protein implicated in DNA repair and transcriptional regulation. Although previous studies have emphasized the necessity of studying APEX1 in a lineage-specific context and its role in progenitor differentiation, no studies have assessed the role of APEX1, nor its two enzymatic domains, in supporting adult HSPC function. In this study, we demonstrated that complete loss of APEX1 from murine bone marrow HSPCs (induced by CRISPR/Cas9) caused severe hematopoietic failure following transplantation, as well as a HSPC expansion defect in culture conditions maintaining in vivo HSC functionality. Using specific inhibitors against either the nuclease or redox domains of APEX1 in combination with single cell transcriptomics (CITE-seq), we found that both APEX1 nuclease and redox domains are regulating mouse HSPCs, but through distinct underlying transcriptional changes. Inhibition of the APEX1 nuclease function resulted in loss of HSPCs accompanied by early activation of differentiation programs and enhanced lineage commitment. By contrast, inhibition of the APEX1 redox function significantly downregulated interferon-stimulated genes and regulons in expanding HSPCs and their progeny, resulting in dysfunctional megakaryocyte-biased HSPCs, as well as loss of monocytes and lymphoid progenitor cells. In conclusion, we demonstrate that APEX1 is a key regulator for adult regenerative hematopoiesis, and that the APEX1 nuclease and redox domains differently impact proliferating HSPCs.

**Graphical Abstract:**

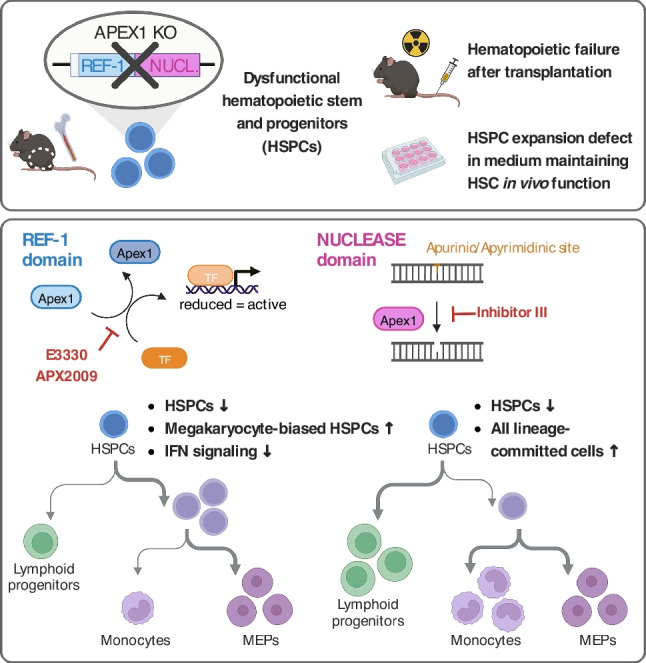

**Supplementary Information:**

The online version contains supplementary material available at 10.1007/s12015-023-10550-0.

## Introduction

Hematopoietic stem cells (HSCs) enable the lifelong process of hematopoiesis, through their self-renewal potential and their long-term multilineage differentiation capacity to replenish all lineage-committed progenitors and mature blood cells. These unique HSC properties are tightly regulated by a complex interplay of both intrinsic and extrinsic cues during both dormancy and proliferation. Despite numerous breakthroughs in the field [1], full understanding of all molecular mechanisms underlying hematopoietic stem and progenitor cell (HSPC) functionality, especially during proliferative demands, remains elusive.

Mammalian apurinic/apyrimidinic endonuclease 1 (APEX1) is a small pleiotropic protein, which is composed of 2 distinct structural catalytic domains [2].

The catalytic C-terminal region of APEX1 exhibits different nucleic acid enzymatic activities. Apurinic/apyrimidinic (AP) endonuclease activity is the predominant nuclease function, with a central role in the base excision repair (BER) pathway. BER is involved in the repair of frequent endogenous DNA single-strand lesions [2]. APEX1 endonuclease processes DNA AP lesions, which can be BER intermediates or *de novo* abasic sites, into single-strand breaks for subsequent gap filling repair [2]. In contrast to other DNA repair pathways [3–9], the role of BER genes such as APEX1 in supporting functional adult HSPCs remains enigmatic. Aside from its canonical function in DNA repair, the APEX1 nuclease domain is also implicated in transcriptional and post-translational regulation [10–12].

The catalytic N-terminal protein domain of APEX1, known as redox effector function (REF-1), controls the DNA binding activity of multiple transcription factors (TFs) through modulation of their redox status [2]. Studies performed chiefly in cancer cell lines have identified TFs such as AP-1, P53, HIF1A, NF-κΒ, PAX5 and STAT3, involved in several cellular responses, as REF-1 targets [13–18]. Almost all the above stated TFs have been implicated in the regulation of HSPC self-renewal and differentiation [19–26].

Considering the multiple functions ascribed to APEX1, we hypothesized that it may be an essential intrinsic regulator of bone marrow (BM) HSPC function. However, studies of the role of APEX1 in adult hematopoiesis and in other tissues have been hampered by the very early embryonic lethality in mice following non-conditional full knock-out (KO) of APEX1 [27, 28]. Cancer cell line studies demonstrated that APEX1 is indispensable for cancer cell survival and proliferation [29–33]. Others demonstrated APEX1 involvement in pro-inflammatory factor expression in lipopolysaccharide-treated macrophage and monocyte cell lines [34–36]. APEX1 KO also impaired class-switch recombination in a B lymphocyte line, without affecting cell viability or proliferation [37]. Only a few studies examined the role of APEX1 in non-transformed cells. APEX1 is required for ex vivo generation, but not survival, of CD34^+^ cells from mouse embryonic stem cells [38]. APEX2, another AP endonuclease, although less efficient than APEX1 [39], was shown to be important during B cell development and regenerative hematopoiesis [40, 41]. In addition, brain-specific [42] KO of APEX1 differently affected development of some but not all neural lineages. Hence, APEX1 appears to play a role in normal progenitor proliferation and lineage differentiation, and this in a cell type and developmental specific manner.

In the current study, we therefore wished to unravel the role of APEX1 and its two main enzymatic domains, in proliferating murine BM HSPCs and during HSPC lineage specification.

## Materials and Methods

Main Methods are described here below, additional Materials and Methods information can be found in the Supplementary Methods.

### Mice

All CRISPR-Cas9 experiments were performed using 8–13 week-old wild type (WT) C57BL/6J-CD45.2, C57BL/6J-CD45.1 (Jackson Laboratory) and homozygous C57BL/6J R26^Cas9GFPdim^ mice (Jackson Laboratory, cat#26179); i.e. ‘Cas9 mouse’. For APEX1 inhibitor experiments, 8–12 week-old WT C57BL/6J-CD45.2 or WT C57BL/6J-CD45.1 males and females were used. All mouse colonies were bred in-house. Transplanted mice were maintained in individually ventilated cages. The KU Leuven animal ethics committee approved all animal experiments (project number P209/2018).

### CRISPR-Cas9 Based Apex1 Knock-Out

The lentiguide vector (Addgene, cat#52963) was modified by replacing the puromycin cassette with a green fluorescent protein gene (i.e. GFP^high^). Two *Apex1* targeting sgRNAs were cloned separately into the plasmid. SgRNA#1 (exon 5) sequence: 5’-GACTGGAATACCGACAGCGT-3’ (GenScript); sgRNA#2 (exon 4) sequence: 5’-ACGGAGCTGACCAGTACTGA-3’ (Sabatini murine library).

### Bone Marrow Derived HSPC Isolation

Mice were sacrificed by cervical dislocation and bone marrow cells were flushed from femurs and tibias using phosphate-buffered saline (Gibco). For the lentiviral transduction and the in vitro expansion experiments, Lin^−^cKit^+^ cells were purified by magnetic-activated cell sorting (MACS) as described in Garcia-Abrego et al*.* [43]. The mean purity (± SD) of MACS-isolated Lin^−^cKit^+^ cells was 68.73 ± 12.16%, with 89.39 ± 10.19% of cells being Lin^−^ cells, 5.14 ± 2.83% LSK cells, and 0.47 ± 0.31% LSK-SLAM cells.

### Bone Marrow Reconstitution Experiment

CD45.1 recipient mice were irradiated twice with 4.5 Gy using an X-ray RS-2000 biological irradiator (Rad Source Technologies) the day before transplantation. 36-48 h after lentiviral transduction, 2.5 × 10^5^ viable Cas9 or WT CD45.2 cells were injected in the tail vein together with 5 × 10^4^ freshly isolated BM CD45.1 cells. During the first 2 weeks after the transplantation, Baytrill (Bayer) was added to the drinking water of transplanted mice. Multilineage differentiation capacity of the GFP^high^ transplanted cells was examined between 2 and 20 weeks post-transplantation using peripheral blood collected via tail puncture. 5 months after transplantation, mice were sacrificed and BM HSPC populations analyzed by flow cytometry.

### HSPC Ex Vivo Culture

Freshly purified Lin^−^cKit^+^ or lentiviral transduced Lin^−^cKit^+^ cells were cultured in polyvinyl alcohol (PVA)-supplemented medium, as described in [44]. Lin^−^cKit^+^ cells were seeded at 10^5^ cells/ml in 24- or 12-well plates, coated with 0.01 mg/ml human fibronectin (Millipore). About 65% of the medium was changed every 2 or 3 days. For the 4-week culture, cells were diluted 1:4 on day 7, 14 and 21. For the albumin-based culture, StemSpan™ SFEM medium (Stem Cell Technologies) was used instead of PVA-supplemented medium. Detailed composition of media can be found in Table S1.

APEX1 inhibitors E3330, APX2009 and APE1 Inhibitor III (Inh. III) (Sigma-Aldrich) were dissolved in dimethylsulfoxide (DMSO). All HSPC cultures were done under 5% O_2_.

### Interferon (IFN) Treatment of REF-1 Inhibited HSPC Cultures

5 × 10^4^ Lin^−^cKit^+^ cells were seeded in 500 µl of PVA-based medium (Table S1) in a 48-well plate, exposed to a REF-1 inhibitor (E3330 5 µM or APX2009 2 µM) or DMSO, and treated continously with 160 ng/ml of recombinant carrier-free mouse IFN-α or IFN-γ (Biolegend). IFNs and REF-1 were refreshed during the 65% medium change at day 3 and 5.

### Flow Cytometry

Flow cytometry was used to assess multi-lineage potential of grafted cells in blood and BM, and HSPC expansion upon APEX1 KO and APEX1 inhibitor treatment, apoptosis, and cell division analysis. Details of the flow cytometry experiments are provided in Supplementary Methods.

### CITE-Sequencing Experiment

Cellular Indexing of Transcriptomes and Epitopes by Sequencing (CITE-Seq) was performed together with the VIB Single Cell Core (Leuven), using 10X Genomics technology. Details about sample preparation, sequencing and bioinformatic analysis are described in Supplementary Methods.

### Quantification and Statistical Analysis

Details regarding statistical analysis, number of experiments and replicates used in each experiment are provided in the figure captions. Raw data prior to matched ANOVA/Mixed model analysis and fold changes were log-transformed. To evaluate whether data were normally distributed, raw, log-transformed or residuals were analyzed using a Shapiro–Wilk test. Log-transformed data were back-transformed for graphical presentation. Statistical testing was performed using GraphPad Prism v9.3.0 (GraphPad Software, Inc.), except for the single cell RNA sequencing (scRNA seq) where testing was done in R (version 4.1.0). A p-value < 0.05 was considered significant.

## Results

### APEX1 is Essential to Maintain HSPC Function during Functional HSC Expansion and Hematopoietic Regeneration

**Fig. 1:**
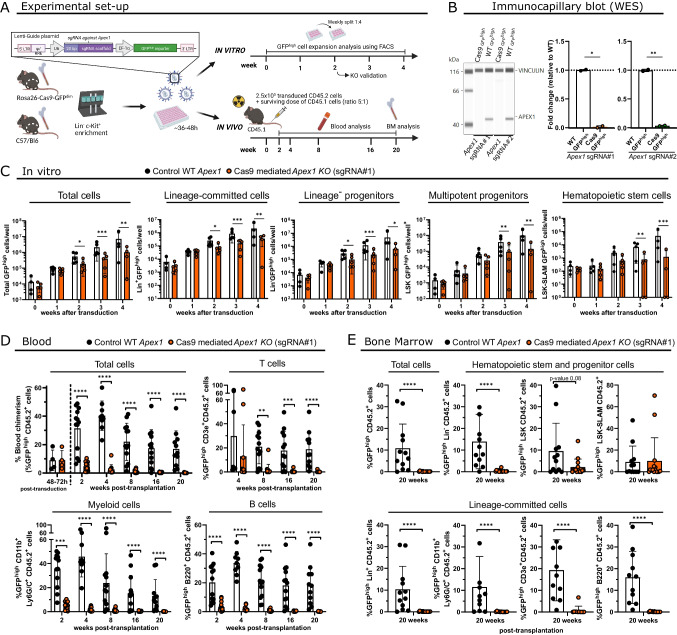
APEX1 is essential to maintain HSPC function during functional HSC expansion and hematopoietic repopulation. (**A**) Experimental set-up to study the role of *Apex1* in HSPCs during long-term in vitro hematopoietic expansion and in vivo hematopoietic regeneration following CRISPR-Cas9 knock-out (KO). (**B**) Immunocapillary blot (WES) showing efficient CRISPR-Cas9 mediated KO of APEX1 in the total progeny of 2-weeks expanded Lin^−^cKit^+^ Cas9 GFP^high^ cells compared to non-edited control cells (progeny of Lin^−^cKit^+^ WT GFP^high^ cells). *N* = 1 with 2 different donors/transductions. Unpaired t test was used to compare Cas9 and WT transduced cells. (**C**) Absolute number of CRISPR-Cas9 mediated APEX1 KO cells (Cas9 GFP^high^ cells, orange dots) or WT cells (WT GFP^high^ cells, black dots), in the total viable cells, Lin^+^, Lin^−^, LSK and LSK-SLAM cells, during a 4-week expansion culture. *N* = 5 independent experiments with 5 independent donors for each group, except for the 4-week time point where *N* = 4. Log scaled axis was used for the expansion graphs. (**D**) Blood percentage of APEX1 KO or WT cells (GFP^high^ cells) present in total and leucocyte lineages (B, T and myeloid) of CD45.2^+^ donor cells at 2-, 4-, 8-, 16- and 20-weeks post-transplantation. Initial transduction efficiency of transplanted cells is shown as percentage of GFP^high^ cells 48–72 h after transduction. N = 4 independent experiments for sgRNA#1, with a total of 9–16 mice per group and timepoint. (**E**) Bone marrow percentage of APEX1 KO or WT cells (GFP^high^ cells) in the total, lineage-committed (Lin^+^ , B, T and myeloid), and HSPC (Lin^−^, LSK and LSK-SLAM) CD45.2^+^ donor cells ± 20 weeks after transplantation. *N* = 4 independent experiments for sgRNA#1, with a total of 12–15 mice per group. Sidak post-hoc tests (following a two-way ANOVA/Mixed model repeated measures analysis) were used to compare the 2 groups (Cas9 and WT) at the different timepoints, in (C) and (D). Mann–Whitney test for all comparisons in (E). Data bars represent the mean ± SD. *p* < 0.05 (*), *p* < 0.01 (**), *p* < 0.001 (***), *p* < 0.0001 (****).

To evaluate the effect of APEX1 loss on adult hematopoiesis, we used a CRISPR-Cas9 KO approach on BM HSPC in combination of an in vitro expansion and an in vivo competitive repopulation assessment (Fig. 1A). Therefore, BM Lin^−^cKit^+^ cells, isolated from Cas9 and WT mice, were transduced with a lentiviral vector, containing an *Apex1* targeting sgRNA and a GFP^high^ reporter. For the ex vivo culture, we used PVA-based medium, that has been shown to maintain repopulating HSCs for up to 1 month (described in Wilkinson et al. [44], and confirmed by our own studies (Figure S2A)), together with the use of 5% O_2_ which helps to selectively enrich for functional HSC populations during the PVA-based expansion, even from unpurified whole BM cells [45]. Highly efficient APEX1 KO by either of the 2 sgRNAs was observed in transduced GFP^high^ progeny 2 weeks post-transduction (Fig. 1B), with no indels detected in predicted off-target regions (Figure S1).

Loss of APEX1 (by both sgRNAs) caused a significant decrease in the relative frequency and expansion of the transduced progeny in the total cells (± 65% expansion decrease for both guides by week 2), lineage-restricted cells (Lin^+^), and the different HSPC populations (Lin^−^, LSK and LSK-SLAM) over the 4 weeks culture period (Fig. 1C, S3A-C). For the HSC population, a ± 76% decrease in total LSK-SLAM expansion was seen after 2 weeks in both APEX1 KO groups.

After transplantation into lethally irradiated recipients, APEX1 KO HSPCs failed to contribute to hematopoietic recovery. Already 2 weeks after transplantation, a significantly lower contribution of APEX1 KO HSPCs (using both sgRNAs) was seen to the total donor leucocyte population (CD45.2^+^), B (B220^+^) and myeloid lineages (Ly6G/Ly6C^+^CD11b^+^) (Fig. 1D, S3D). In line with the blood chimerism, contribution of APEX1 KO cells (both sgRNAs) to the different BM populations 20 weeks after transplantation (Fig. 1E, S3E) was significantly reduced (Lin^+^, B-, T-, myeloid and Lin^−^ progenitor compartments). APEX1 KO multipotent progenitors (LSK cells) were also decreased significantly for sgRNA#2 and almost significantly for sgRNA#1 (*p*-value = 0.08). Although APEX1 KO HSCs (LSK-SLAMs) were clearly unable to generate committed progeny, no significant change was seen between the APEX1 KO and WT LSK-SLAM compartment 20 weeks after transplantation (both sgRNAs), which might be in part due to low-level engraftment of lentivirally transduced LSK-SLAM cells [46, 47], as seen in BM of control mice. No evidence for malignant hematopoiesis in APEX1 KO grafted animals was seen 5 and 12 months after transplantation (Figure S4). Moreover, we demonstrated that there was no repopulation difference at 20 weeks post-transplantation between the non-transduced Cas9 expressing HSCs (GFP^dim^) and the non-transduced WT HSC (GFP^neg^) (Figure S2B).

Overall, our results showed that APEX1 KO in adult HSPCs causes an ex vivo expansion and in vivo hematopoietic repopulation defect.

### APEX1 REF-1 and Nuclease Inhibitions lead to HSPC Expansion Deficits, which are Associated with Enhanced Apoptosis and Decreased Cell Divisions

**Fig. 2:**
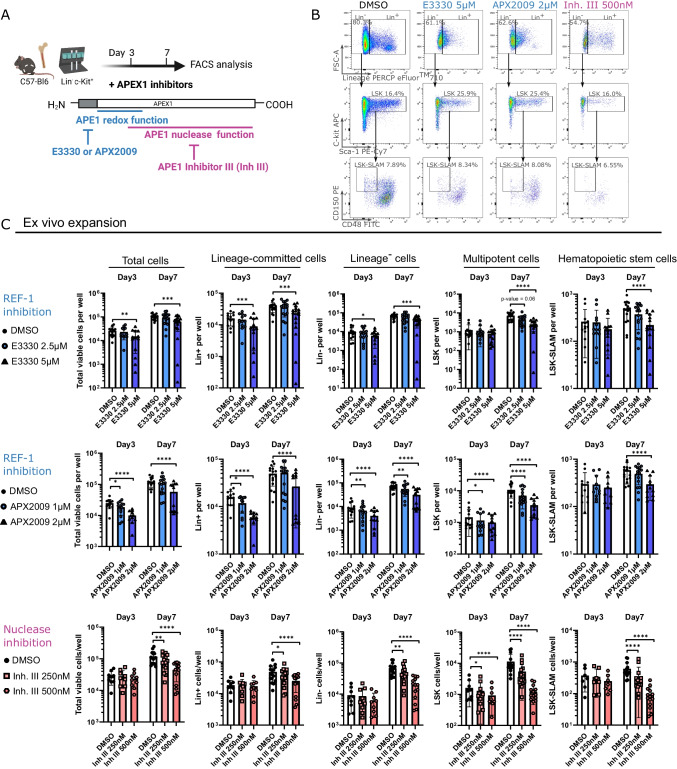
APEX1 nuclease and REF-1 functions are both essential for functional HSPC expansion. (**A**) Schematic representation of the experimental set-up to elucidate the dual role of APEX1 in HSPCs during in vitro expansion, using inhibitors either against the APEX1 redox function (REF-1) (E3330 and APX2009) or APEX1 nuclease function (Inh. III). (**B**) Representative flow cytometry plots showing the different APEX1 inhibitor or DMSO treated HSPC populations on day 7. (**C**) Total expansion (number of cells/well) of the Lin^−^cKit^+^ progeny exposed continuously for 3 or 7 days to low concentrations of E3330 or APX2009 REF-1 inhibitors, or Inh. III nuclease inhibitor. *N* = 5–8 independent experiments, with a total of 9–16 donors per group. Dunnett’s post hoc tests (following a two-way ANOVA/Mixed model sample-matched analysis) were used to compare each treated group to their corresponding DMSO control condition. Data bars represent the mean ± SD. Log scaled axis was used for the expansion graphs. *p* < 0.05 (*), *p* < 0.01 (**), *p* < 0.001 (***), *p* < 0.0001 (****).

To investigate which functional domain(s) of APEX1 affect(s) HSPC function and due to difficulties in creating domain-specific KO in primary HSPCs, we added small molecules that specifically inhibit the nuclease (using Inh. III) [48] or the REF-1 (using E3330 or APX2009) [49, 50] function of APEX1 to PVA-based WT Lin^−^cKit^+^ cultures for 3- or 7-days (Fig. 2A), after identifying an effective concentration with the lowest toxicity (Figure S5A). Inh. III is a competitive inhibitor, binding the active site of the endonuclease domain of APEX1 [48]. E3330 is a REF-1 selective inhibitor that interferes with disulfide bond formations between cysteine residues within the active site of APEX1’s redox domain [51] and is currently being used in different clinical trials as cancer treatment (NCT0337508, NCT03375086). APX2009 is a second generation E3330 analogue, which was found more potent compared to E3330 [52]. All 3 inhibitors have been previously used in diverse studies to decipher the distinct role(s) of the 2 main APEX1 domains [12, 34–36, 38, 52, 53].

REF-1 inhibition by both E3330 and APX2009 (Fig. 2B-C) caused a significant expansion defect in total cells, committed cells (Lin^+^) and Lin^−^ progenitors, already on day 3. After 7 days, the expansion of total, Lin^+^, Lin^−^, LSK and LSK-SLAM cells was significantly impeded, with a clear decreased trend for the lowest concentration of inhibitors as well. Following 7-days 5 µM E3330 and 2 µM APX2009 exposure, a ± 54% and ± 50%, respectively, decrease was seen in the number of LSK-SLAMs compared to the DMSO control. Interestingly, we observed a dose dependent increase in the relative proportion of HSPCs (LSK and LSK-SLAM) on day 3 and of LSK-SLAM cells on day 7 compared to control, suggesting an early accumulation of HSPC among the culture progeny (Figure S5B).

Addition of the nuclease inhibitor (Fig. 2B-C) caused a significant expansion defect of LSK cells on day 3, while not affecting the other compartments. By day 7 all cell populations were decreased by Inh. III (± 84% decrease in LSK-SLAM cells for Inh. III 500 nM). In contrast to cultures treated with the REF-1 inhibitors, Inh. III induced a clear dose-dependent decrease in the proportion of LSK and LSK-SLAM cells, compared to controls (Figure S5B).

**Fig. 3:**
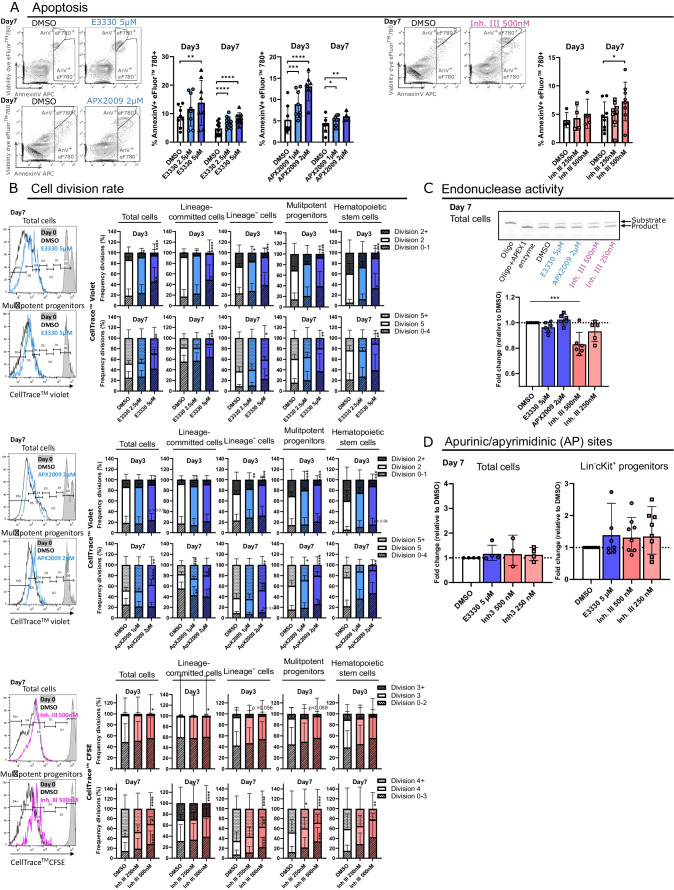
Inhibition of APEX1 REF-1 and nuclease functions in HSPCs leads to enhanced apoptosis and decreased cell proliferation. (**A**) Total apoptotic cells were quantified by flow cytometry using Annexin V and eFluor^TM^780 staining (*N* = 2–5 indep. exp., with 2 biological replicates per experiment) after 3 and 7 days of continuous APEX1 inhibitor exposure. (**B**) Cell divisions of the Lin^−^cKit^+^ progenies were analyzed using CellTrace dyes (*N* = 3 indep. exp., with 5–6 biological replicates in total) after 3 and 7 days of continuous APEX1 inhibitor exposure. (**C**) Apurinic/apyrimidinic (AP) incision assays to measure the endonuclease activity in total progeny of 7-days expanded HSPCs treated with APEX1 inhibitors. *N* = 2 indep. experiments with total of 6 biological replicates per group. (**D**) AP site quantification on total progeny or Lin^−^cKit^+^ sorted cells after ex vivo treatment with APEX1 inhibitors. *N* = 2 indep. experiments with a total of 3–4 biological replicate per group for the total cells, and *N* = 4 with 8 replicates for the Lin^−^c-Kit^+^ cells. Dunnett’s post-hoc tests were used to compare each treated group to their corresponding DMSO control condition (following a two-way ANOVA/Mixed model sample-matched analysis in (A) and (B), or a one-way ANOVA in (C) and (D)). Data bars represent the mean ± SD, except for (C) and (D) where data bars represent geometric mean ± geometric SD. *p* < 0.05 (*), *p* < 0.01 (**), *p* < 0.001 (***), *p* < 0.0001 (****).

Both REF-1 inhibitors caused a concentration-dependent increase in apoptotic cells on day 3 and 7, while the nuclease inhibitor induced apoptosis only on day 7 (Fig. 3A). Almost all HSPC progenies showed reduced numbers of cell divisions on days 3 and 7 in response to both REF-1 and nuclease inhibitors, indicating that both APEX1 functions are necessary to support HSPC division (Fig. 3B).

As a recent study suggested that expanded functional HSCs can be identified as EPCR (CD201) positive cells among the LSK cells [54], we also quantified the number of LSK EPCR^+^ HSCs following REF-1 or nuclease inhibition. In line with the LSK-SLAM quantification, the expansion of LSK EPCR^+^ cells was also significantly decreased in REF-1 inhibitor and nuclease inhibitor treated HSPCs (Figure S5C).

In line with the severe repopulation phenotype seen in the APEX1 KO HSPCs, the combined REF-1 and nuclease inhibitor treatments led to significantly more cell death and a bigger HSPC expansion defect than following addition of the nuclease or REF-1 inhibitors alone (Figure S6).

The nuclease domain plays a role in DNA repair by removing/processing ‘baseless’ AP sites. Therefore, we assessed changes in the DNA endonuclease activity and AP site accumulation in the total progeny and/or progenitor population following the inhibitor treatments. As expected APEX1 nuclease inhibitor (Inh. III 500 nM) decreased the endonuclease activity in the HSPC progeny in contrast to both REF-1 inhibitors, which did not influence the AP endonuclease activity. Even though we noticed the partial impairment in the DNA endonuclease activity, the 7-day Inh. III treatment did not cause DNA AP site accumulation in the total and Lin^−^cKit^+^ progeny cells (Fig. 3C-D). This was contrary to the APEX1 KO, which caused a complete absence of endonuclease activity and a slight increase in DNA damage (AP site and γH2AX-positve cells increased) in HSPCs (Figure S7).

These data support that both APEX1 REF-1 and nuclease domains are required for HSPC proliferation. The HSPC expansion deficit induced by REF-1 and nuclease inhibitors is associated with increased cell death and reduced cell divisions, but no significant accumulation of AP lesions could be detected.

### CITE-Seq Revealed that HSPC Populations were Affected Differentially upon APEX1 REF-1 and Nuclease Deficiencies

**Fig. 4:**
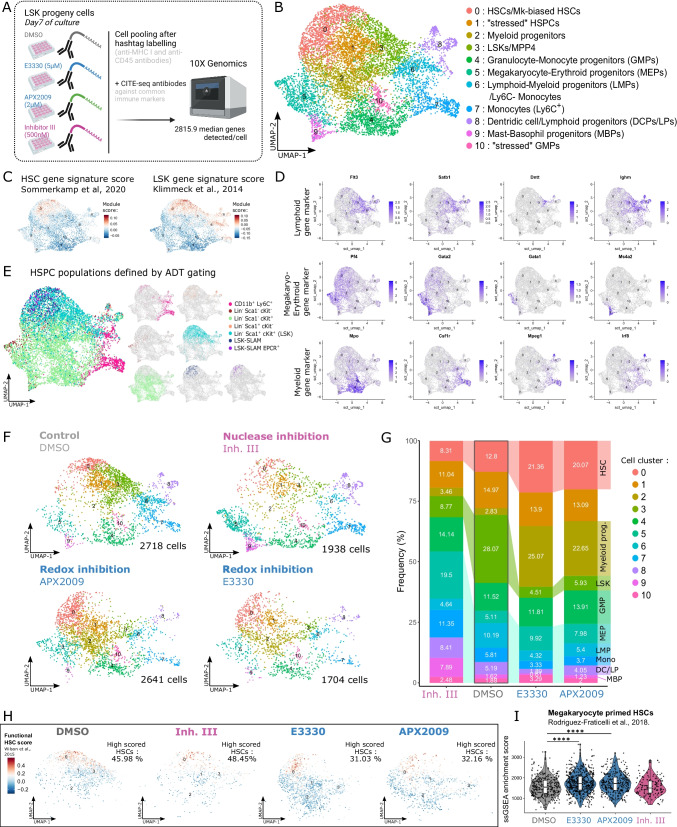
CITE-seq revealed that HSPC populations were affected differentially upon APEX1 REF-1 and nuclease inhibition. (**A**) Overview of 10X Genomic CITE-seq experimental design. (**B**) RNA-UMAP (Uniform Manifold Approximation and Projection) representation of the APEX1 inhibitor-treated and control HSPC samples, revealing 10 distinct cell clusters. (**C**) UMAPs showing the Seurat module enrichment score for the HSC and LSK transcriptomic signatures respectively. (**D**) Expression of some selected gene markers, highlighted on the RNA-UMAP, used to identify the different cell identities in the CITE-seq analysis. (**E**) Antibody-derived tag (ADT) gated HSPC populations, highlighted on UMAP from RNA based clustering. (**F**) UMAP plots for the control treated HSPCs (DMSO), the APEX1 nuclease inhibited HSPCs (Inh. III), and the APEX1 REF-1 inhibited HSPCs (E3330 and APX2009). (**G**) Frequencies of the different annotated CITE-seq clusters, for each of the treated HSPC cultures. Chi-square test was performed on cluster proportions (see Table S6) (**H**) UMAP of cluster 0, 1, 2, and 3 displaying the module score for the gene signature of functional HSCs, as well as the percentage of functional HSCs within the HSC cluster 0 (cut-off = 0.2). (**I**) Single-sample gene set enrichment analysis (ssGSEA) for megakaryocyte primed HSC signature (from Rodriguez-Fraticelli et al., 2018) on cluster 0. Dunn’s post hoc tests (following Kruskal–Wallis rank sum test) were used to compare each treated group to the DMSO. *p* < 0.05 (*), *p* < 0.01 (**), *p* < 0.001 (***), *p* < 0.0001 (****).

To gain insight in the mechanism(s) underlying APEX1 redox and nuclease mediated deficits in HSPC proliferation, we performed 10X CITE-seq on LSK cell progeny 7 days after culture in PVA-based medium with APEX1 inhibitors (Fig. 4A). Using RNA and antibody-derived tags (ADT) data (Fig. 4C-E, S9-10), 10 distinct CITE-seq clusters were identified on the UMAP from all 4 samples (Fig. 4B).

APEX1 nuclease inhibition induced decreased frequencies of HSC, LSK and lymphoid-myeloid progenitor (LMP) cells, while the granulocyte-monocyte progenitor (GMP), megakaryocyte-erythroid progenitor (MEP), monocyte, dendritic cell and lymphoid progenitor (DCP/LP), and mast-basophil progenitor (MBP) clusters were enriched (Fig. 4F-G, Table S6). ADT data also showed increased percentages of lineage committed cells (Lin^+^) and progenitors (Lin^−^Sca1^−^cKit^−^ and Lin^−^Sca1^−^cKit^+^ cells), while the LSKs and HSCs (LSK-SLAM and LSK-SLAM EPCR^+^) were reduced (Figure S10D, Table S7). Following APEX1 REF-1 inhibition, a different cluster distribution was seen. Treatment with either E3330 or APX2009 caused an enrichment in HSC and MEP clusters, while the LSKs, LMPs, DCPs/LPs, MBPs and monocytes were significantly decreased (Fig. 4F-G, Table S6). Additionally, REF-1 inhibition resulted in the appearance of a new progenitor cluster (cluster 2), which were mostly Lin^−^Sca1^−^cKit^+^ cells (Figure S10D) expressing early myeloid and megakaryocyte markers (Fig. 4D, S9A). ADT data confirmed decreased monocytes (CD11b^+^Ly6C^+^ cells), increased LSK-SLAM and LSK-SLAM EPCR^+^ frequencies upon REF-1 inhibition (Figure S10D, Table S7), consistent with previous flow cytometry data on day 3 (Figure S5B). REF-1 inhibition also decreased the percentage of HSCs displaying a functional HSC gene signature [55], which was not seen following nuclease inhibition (Fig. 4H). Single sample gene set enrichment analysis (ssGSEA) confirmed that REF-1 inhibition induced expression of megakaryocyte-biased genes in the HSCs (cluster 0), compared to control and Inh. III treated cultures (Fig. 4I).

REF-1 and nuclease functions of APEX1 are both important for HSPC maintenance during in vitro expansion, but their inhibition leads to highly distinct transcriptional changes, as revealed by the differences in the CITE-seq-defined cell identities.

### Differential Gene Expression and Regulon Activity Analyses show Mainly Downregulation of Interferon Signaling in HSPC Populations caused by APEX1 REF-1 Deficiency

To reveal the mechanisms underlying the distinct distribution of the cell populations upon APEX1 inhibition, we performed differentially expressed gene (DEG) and differentially activated regulon (DAR) analyses following REF-1 and nuclease inhibitions (Supplement File 5–7).

**Fig. 5:**
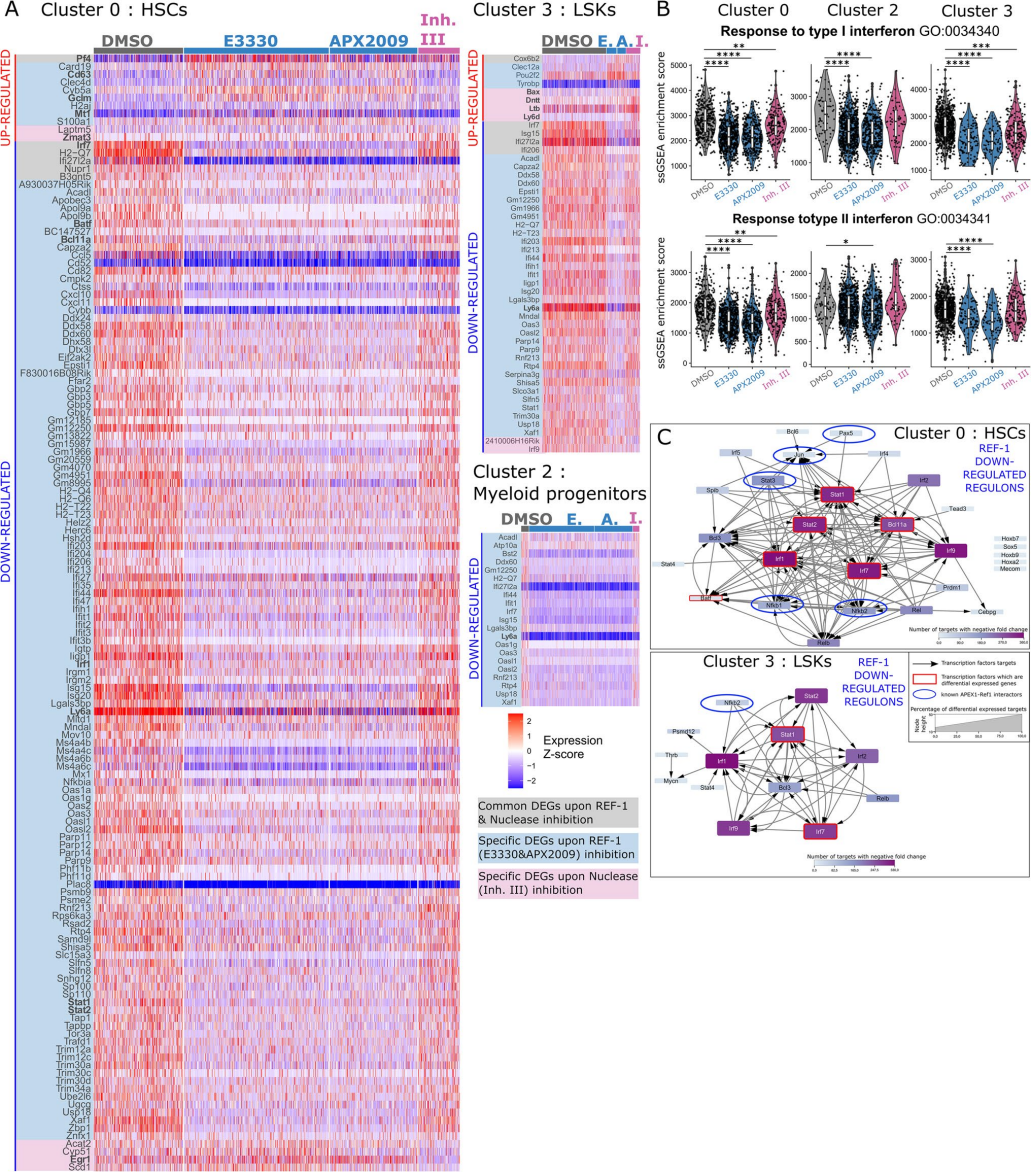
Differential gene expression and regulon activity analyses identify chiefly downregulated interferon signaling in HSPC progeny following APEX1 REF1 inhibition. (**A**) Heatmaps displaying differentially expressed genes (DEGs) in the HSC cluster 0, the LSK cluster 3 and the early myeloid progenitor cluster 2, after 7-days of APEX1 REF-1 (E3330 and APX2009) or APEX1 nuclease (Inhibitor III) inhibition compared to DMSO treated cells. DEGs common to all 3 inhibitor treatments are highlighted in gray, DEGs present after both REF-1 inhibitions are highlighted in blue and DEGs only present after nuclease inhibition with Inh. III are highlighted in pink. (**B**) Single-sample gene set enrichment analysis (ssGSEA) for interferon responses of cluster 0, 2, and 3 (HSCs, early myeloid progenitors, and LSKs) using published gene lists (GO:0034340 for type I interferon and GO:0034341 for type II interferon). Dunn’s post hoc tests (following Kruskal–Wallis rank sum test) were used to compare each treated group to the DMSO. *p* < 0.05 (*), *p* < 0.01 (**), *p* < 0.001(***), *p* < 0.0001 (****). (**C**) Cytoscape representation of the specific downregulated regulons upon both APEX1-REF-1 inhibition for the HSC cluster 0 and the LSK cluster 3. The nodes are the differentially active transcription factors (TFs) following REF-1 inhibition, and the node size represents the percentage of target genes of each TF that are differential expressed. The node gradient color (from light blue to dark purple) represents the number of target genes that are negatively regulated compared to DMSO condition (log2 fold change <0). TFs that are among the DEGs are highlighted by red squares, and the TFs that were previously published to interact directly with REF-1 are highlighted by blue circles. The black arrows indicate when a TF has another TF as target gene.

REF-1 inhibition caused transcriptional changes in HSC, LSK and myeloid progenitor clusters (0, 3 and 2), consisting mainly of downregulated DEGs, with prominent downregulation of interferon response genes (IRGs) (Fig. 5A). Consistently, the HSPC marker *Ly6a*, reported to be upregulated in HSCs in response to IFNα [56], was downregulated in all progenitor clusters. REF-1 inhibition also downregulated the interferon type I and II response in HSCs, LSKs and myeloid progenitors as reflected by the ssGSEA enrichment score for the respective gene ontogeny terms (Fig. 5B). Other downregulated DEGs included the TFs, *Batf* and *Bcl11a*, shown to be essential for HSC functionality and lymphoid lineage development [57–60]. Among the few upregulated DEGs in REF-1-inhibited HSCs, we identified marker genes that were among the highly expressed in the myeloid progenitor, MEP and BMP clusters (*Cd63, Gclm, Mt1*) (Fig. 5A, Figure S9A). In line with DEG analysis, DAR analysis for REF-1 inhibitor treated HSCs and LSKs showed a decreased activity for many interferon-related TFs (*Stat1, Stat2* and several *Irfs*), and for the nuclear factor-kappa B (NF-κB) family and related factors (*NF-κB1, NF-κB2, Relb, Rel, Bcl-3*) (Figure S11A). *Stat1, Stat2, Irf1, Ir7, Bcl11a and Irf9* were found central TFs driving the negative regulation of most target genes in the REF-1-inhibited HSCs (Fig. 5C), and are possibly controlled by previously identified REF-1 targets (*Jun, Pax5, Stat3 and Nf-κb1*) [13, 14,16, 17].

Among the upregulated regulons in HSCs, we identified *Cebpβ,* which induces myeloid differentiation and proliferation in stressed HSCs [61], as well as TFs (*Gata1*, *Mafg, Nfe2, Kfl1, Fli, Myb*) that control myeloid and megakaryocyte differentiation [62–64]. Some of these latter pro-differentiation TFs were also found among upregulated DARs in LSKs (Figure S11B).

In the more committed progenitor clusters (GMPs, MEPs, LMPs, monocytes and DCPs/LPs), REF-1 inhibition also caused downregulation of IRGs, and/or interferon related regulons (*Stat1, Stat2, Irf-1, Irf-7, Irf-9*). Additionally, LMPs, DCPs/LPs and monocytes appeared to be transcriptionally megakaryocyte-poised, reflected by upregulated MEP-specific regulons such as *Gata2, Tal1, Fli, Nfe2,* and *Mafg* [62, 63] (Figure S12).

Thus, the APEX1 REF-1 function appears to regulate interferon related TFs and genes in proliferating HSPCs. Decreased expression of IRGs due to REF-1 inhibition leads to impaired expression or activity of stem cell-essential genes/TFs, while also priming HSC and different progenitor cells towards the megakaryocyte lineage at the detriment of the monocyte/lymphoid lineages.

### HSPC Differentiation Trajectories of APEX1 Nuclease-Inhibited Cells Highlight Earlier Upregulation of Lineage-Restricted Regulators

In contrast to the REF-1 inhibitors, much fewer transcriptional changes were induced in HSPCs by APEX1 nuclease inhibition (Fig. 5, S11A). The p53 target gene *Zmat3*, known to play a role in translation regulation [65], was upregulated in HSCs. Additionally, in both HSC and LSK clusters, a pro-apoptotic signature was detected with the upregulation of *Bax* and higher activity of the *Zfp110* regulon [66, 67]. Pro-differentiation changes were also observed with an increased expression of the megakaryocytic marker *Pf4* gene in HSCs, and the upregulation of lymphoid markers (*Dntt Ltb, Ly6d*) in LSK cells. Moreover, and in contrast to REF-1 inhibition, nuclease inhibition decreased expression of *Egr-1* and its regulon *Fosl1*, where downregulation of the *EGR-1* network was recently linked to impaired HSC function [68]. Although downregulation of some IRG genes was also seen in Inh. III treated HSC and LSK clusters, this was much less profound compared to the REF-1 inhibition.

While REF-1 inhibition enhanced expression/activity of chiefly pro-megakaryocytic progenitor markers, nuclease inhibition promoted differentiation towards all blood lineages. For instance, Inh. III exposure induced the granulocyte and monocyte master regulons *Cebpe* and *Irf8* [69, 70] in GMPs; the B cell and monocyte/macrophage developmental regulator *Prdm1* [71] in LMPs; *Runx2* and *Klf2,* involved in plasmacytoid dendritic cell (DC) [72] and Ly6C^low^ monocyte development [73] respectively, in the DCP/LP cluster; and the erythroid *Klf-1* regulon [62] in MEPs (Figure S12C).

**Fig. 6:**
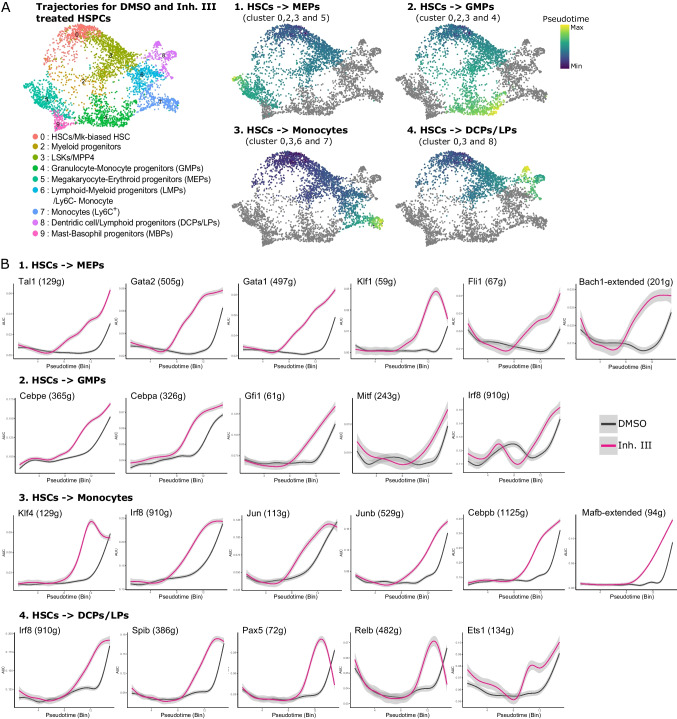
Earlier activation of differentiation regulons following APEX1 nuclease inhibition in HSPCs. (**A**) Pseudotime inferred by Slingshot for 4 distinct trajectories (megakaryocyte-erythroid progenitor (MEP), granulocyte-monocyte progenitor (GMP), monocyte and dendritic cell/lymphoid progenitor (DCP/LP)) of DMSO and Inh. III treated HSPCs is shown on the RNA-UMAP. For each of the 4 trajectories, the HSCs (cluster 0) were defined as root cells. Clusters of interest for each trajectory were subsetted prior to *Slingshot* analysis. Cluster 0, 2, 3, and 5 were used for the MEP trajectory, cluster 0, 2, 3, and 4 for GMP trajectory, cluster 0, 3, 6, and 7 for the monocyte trajectory, and cluster 0, 3, and 8 for the DCP/LP trajectory. Cells of clusters displayed in gray were not used for the respective pseudotime analysis. (**B**) Selected differentiation regulons for each of the 4 defined trajectories and their median regulon activity along the pseudotime. These selected regulons are among the regulons that are significantly differentially active along the pseudotime between the nuclease inhibitor (pink line) and DMSO (black line) treated cultures (entire list of differentially active regulons in Figure S13, Supplement File 8). Number indicated in between brackets after the regulon is the number of targets identified in the entire data set by SCENIC analysis.

To further investigate the apparent increased maturation in nuclease-inhibited progeny, we performed trajectory inference analysis for the MEP, GMP, monocyte and DCP/LP lineages on DMSO and nuclease inhibitor treated samples (Fig. 6A). Many regulons were differentially active along the pseudotimes between the nuclease-inhibited and control cells (Figure S13). For each of the 4 trajectories, APEX1 nuclease-inhibited samples displayed an earlier enhanced activity of master TFs (regulons) promoting differentiation (Fig. 6B). Pro-MEP (*Tal1, Gata2, Gata1*) [62], pro-erythroid (*Klf1*) [62], pro-megakaryocyte (*Fli*) [62] and pro-myeloid (*Bach1)* [74] TFs in the MEP trajectory; granulopoiesis (*Cebpe, Cebpa, Gfi1*) [69], mast cell development (*Mitf*) [75] and monocyte differentiation (*Irf8*) [70] regulons in the GMP trajectory; monocytic (*Irf8, Klf4, Jun, Junb*, and *Cebpb)* [70] and macrophage (*Mafb*) [76] differentiation TFs in the monocyte trajectory; TFs necessary for plasmacytoid DC differentiation (*Irf8* and *SpiB)* [72], B development cell (*Pax5*) [22], development/maturation of DC, B-, T- and NK cells (*RelB and Ets-1)* [77, 78] in the DCP/LP trajectory; were all active in the nuclease inhibitor sample before being active in the DMSO sample (Fig. 6B).

Overall, the transcriptomic data demonstrated that the APEX1 nuclease function, contrary to the redox function, has only a minor gene regulating function in HSPCs. However, its inhibition switches on differentiation programs towards all hematopoietic lineages, thus promoting HSPC commitment.

### APEX1 REF-1 Function was Dispensable during HSPC Proliferation in Inflammatory Cytokine Inducing Conditions

**Fig. 7:**
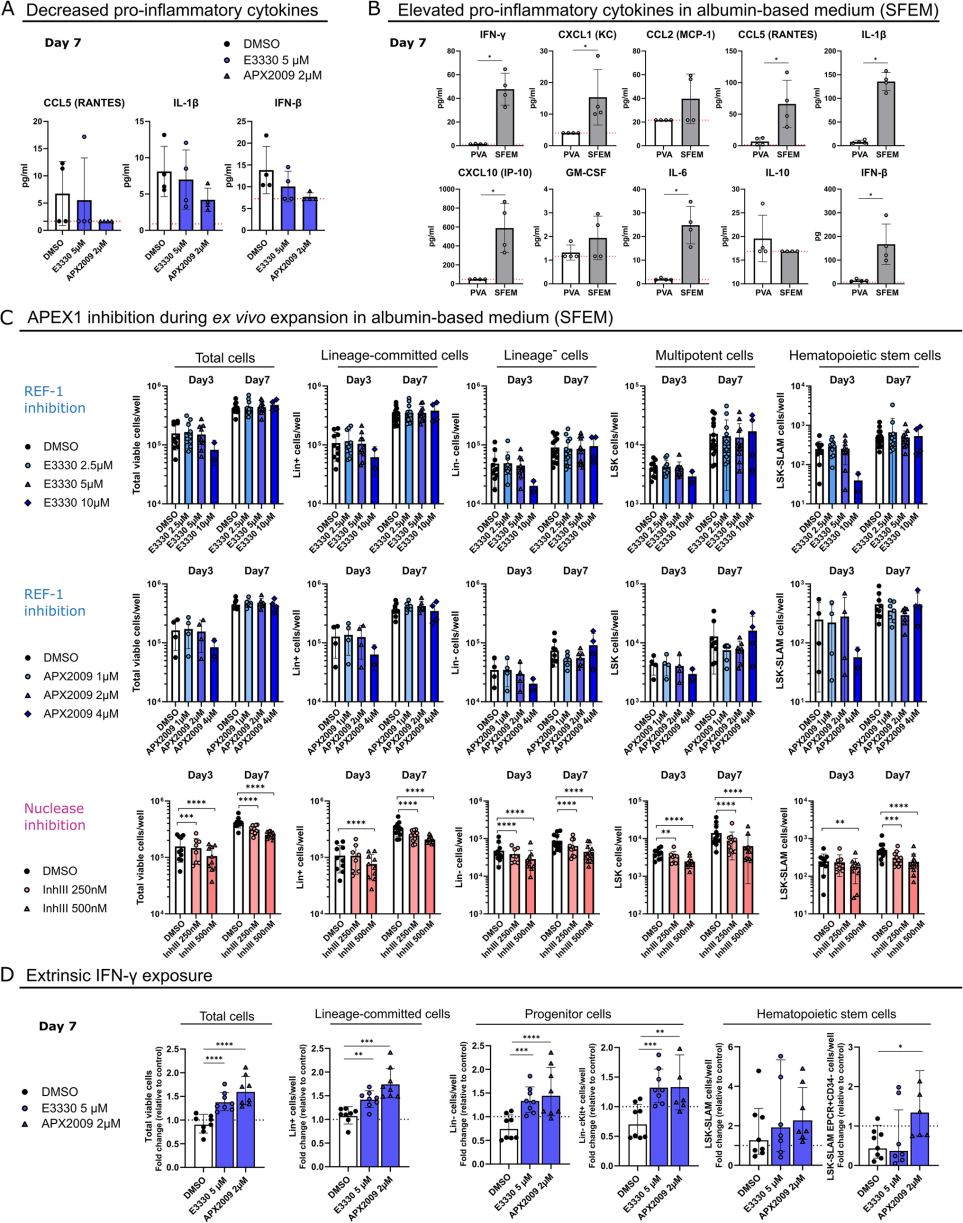
APEX1 REF-1 function was dispensable during HSPC proliferation in inflammatory cytokine inducing conditions. (**A**) CCL5, IL-1β and IFN-β levels in the supernatant of day 7 HSPC cultures in the presence of REF-1 inhibitors. *N* = 2 independent culture experiments, with 2 biological replicates per experiment. (**B**) Cytokine measurement in the supernatant of PVA- and SFEM-based HSPC cultures after 7 days. *N* = 2 independent culture experiments, with 2 biological replicates per experiment. Mann–Whitney test was used to compare the groups. (**C**) Expansion of the different Lin^−^cKit^+^ progeny exposed continuously for 3 or 7 days to the E3330 or APX2009 REF-1 inhibitors, or the Inhibitor III (Inh. III) nuclease inhibitor in albumin-based medium (SFEM). Log scaled axis was used for the expansion graphs. *N* = 2–7 independent experiments, with a total of 4–13 donors per group for day 7, and N = 1–6 independent experiment for day 3, with a total of 2–10 replicates per group. (**D**) Expansion of HSPC progeny (total cells, Lin^−^, Lin^−^cKit^+^, LSK-SLAM and LSK-SLAM EPCR^+^CD34^−^) following IFN-γ treatment with and without REF-1 inhibitors. Fold change of expansion was calculated for each sample relative to their corresponding non-IFN-γ control with and without REF-1 inhibitors. *N* = 4 independent culture experiments, with a total of 6–8 biological replicates per group. Dunnett’s post hoc tests (following a one-way (in (D)) or two-way (in (C)) ANOVA/Mixed model matched analysis) were used to compare each treated group to their corresponding DMSO control condition. Data bars represent the mean ± SD, except for (D) where data bars repesent geometric mean ± geometric SD *p* < 0.05 (*), *p* < 0.01 (**), *p* < 0.001 (***), *p* < 0.0001 (****).

The transcriptomic data surprisingly showed that decreased expression of IRGs might underlie HSPC expansion and survival defects following REF-1 inhibition. Indeed, when we measured inflammatory cytokine/chemokine concentrations in HSPC-conditioned PVA medium in the presence of REF-1 inhibitors, the already very low concentrations of CCL5, IL-1β, and IFN-β appeared to decrease even further (Fig. 7A). However, it is commonly believed that high levels of pro-inflammatory cytokines or IFN-α/γ treatment impair HSC functionality and ex vivo expansion [44, 79–81].

In all the above HSPC expansion experiments, we used a PVA-supplemented medium described to reduce inflammatory factors secreted by HSCs and their progeny and thereby allowing expansion of in vivo repopulating HSCs, compared to albumin containing medium [44]. During hematopoietic development where—as in our in vitro PVA-based culture system—HSPCs are highly proliferative, IFN signaling is required for HSPC functionality [82, 83]. This let us to hypothesize that although REF-1 function is required for functional HSPC expansion in medium containing low levels of inflammatory factors (PVA-based), it might be dispensable for HSPC under culture conditions (albumin-containing, such as SFEM), that do not support functional expansion of the immature HSPC compartment but are associated with fast differentiation of HSPCs into Lin^+^ cells and production of high levels of inflammatory cytokines (Fig. 7B, S14).

In line with our hypothesis, no significant HSPC expansion defect was observed following REF-1 inhibition in SFEM culture conditions (Fig. 7C, S15A). E3330 or APX2009 did neither affect the percentage, nor the absolute number of cells on day 3 or day 7, and this for all the different HSPC progeny populations. Even when we increased the inhibitor concentration, no expansion defect was seen. By contrast, addition of the nuclease inhibitor to the SFEM cultured cells resulted in a similar expansion defect as seen in PVA-based medium (Fig. 7C, S15A).

To further demonstrate that the effect of REF-1 inhibitors on HSPC expansion in PVA-based medium is mediated by loss of IRGs, we repeated the cultures adding a low concentration of IFN-γ or IFN-α for 7 days in the presence of the REF-1 inhibitors. Addition of IFN-γ to the control DMSO cultures tended to decrease progenitors (Lin^−^ and Lin^−^cKit^+^) and HSCs (defined as LSK-SLAM EPCR^+^CD34^−^cells, to enrich for functional HSCs after IFN exposure [84]). By contrast, addition of IFN-γ to the REF-1 inhibitor treated HSPCs increased the total cell number, progenitors (Lin^−^ and Lin^−^cKit^+^) as well as HSCs (LSK-SLAM EPCR^+^CD34^−^), even if the latter was only significant for APX2009. Thus, IFN-γ could partially rescue the REF-1 mediated expansion defect of HSPCs cultured in PVA-based medium (Fig. 7D, Figure S15B). However, IFN-α did not significantly influence the expansion of REF-1 treated HSPCs (Figure S15C).

These studies demonstrate that the APEX1 REF-1 mediated inhibition of interferon related TFs and genes in proliferating HSPCs is only observed when HSPCs are cultured in PVA-based medium, which limits production of inflammatory cytokines and expands functional HSCs. Consistently, addition of exogenous IFN-γ to PVA-based medium could partially rescue the effect of the REF-1 inhibitor, APX2009, on HSPC expansion.

## Discussion

We provide here, to our knowledge, the first evidence that APEX1 is an intrinsic key regulator for adult hematopoiesis, as APEX1-deficient HSPCs were unable to restore the blood system following transplantation. This in vivo phenotype was also recapitulated under culture conditions maintaining functional HSCs [44]. Use of specific APEX1 domain-blocking chemicals demonstrated that both the APEX1 nuclease and redox activities are crucial for the support of HSPC and lineage-committed progenitor survival and proliferation. Single-cell transcriptomics of HSPCs and their progeny identified distinct mechanisms responsible for hematopoietic defects induced by nuclease and redox APEX1 inhibition. Whereas inhibition of the APEX1 nuclease function induced an early activation of differentiation programs, inhibition of the APEX1 redox function significantly downregulated IRGs in HSCs and their progeny, which appears to be required for maintenance and expansion of HSPC culture conditions maintaining in vivo repopulating HSCs [44].

Most studies evaluating the role of APEX1 nuclease and redox domains in different cell types and tissues found one catalytic function to be the most relevant [29, 38, 85–87]. REF-1, not the nuclease function, was needed to generate mouse embryonic CD34^+^ progenitors in vitro [38]. By contrast we provide evidence that both the APEX1 nuclease and redox domains are indispensable to support adult BM HSPC proliferation, and that inhibitors of either domain differentially affected HSPCs and their progenies.

Indeed, even though decreased HSPC expansion, enhanced apoptosis, and reduced cell division were induced by REF-1 and nuclease inhibitors, single-cell CITE-seq analysis demonstrated distinct transcriptional changes underlying the expansion defects following inhibition of either domain. In the presence of the APEX1 nuclease inhibitor, a significant decrease in HSCs and multipotent progenitors was observed, with a concurrent increased frequency of lineage-committed cells and progenitors. The higher vulnerability of HSPCs, compared to the more committed cells following nuclease inhibition, was associated with pro-apoptotic and pro-differentiation transcriptional changes in HSPCs. Cell division-uncoupled HSC differentiation into downstream lineages has recently been reported [88]. Hence, nuclease deficiency favors HSPC fate specification over maintenance, while simultaneously limiting their self-renewal activity. Interestingly, HSCs with accumulated DNA damage can be forced either into differentiation to limit their self-renewal capacity [58, 89], or into DNA damage-induced apoptosis [90]. Although our scRNA seq data showed upregulation of *Wig-1*, a p53 target gene induced in HSPCs after in vivo exposure to benzene or 5-fluoro-uracil [91, 92], we could not detect enhanced DNA damage lesions after 7 days of Inh. III exposure. It is possible that under nuclease inhibition conditions, other DNA repair proteins may be able to resolve the AP DNA lesions in an APEX1-independent manner [39, 93, 94], even if APEX1 is believed to be the most effective AP site-processing enzyme in mammals.

HSPC expansion loss might also be caused by the non-DNA repair functions of the nuclease APEX1 domain. For instance, the APEX1 nuclease domain has been shown to modify gene transcription by affecting RNA decay and/or miRNA processing [11, 12], or even through its non-canonical role in DNA demethylation [95]. In line with the latter example, HSPC dysfunction has recently been correlated to DNA hypermethylation and chromatin accessibility changes, which were associated with downregulation of the *Egr-1* network [68]. We demonstrated that *Egr-1*, a TF that can regulate HSC proliferation [96], was specifically downregulated in HSCs upon Inh. III treatment. Finally, the APEX1 nuclease domain might also affect hematopoiesis by regulating cell fate decisions, as has been described for instance for stem cell differentiation towards the neuroectoderm lineage and stem cell reprogramming [97].

Following APEX1 REF-1 inhibition with E3330 or APX2009, HSCs and their downstream lineages were biased towards the megakaryocyte lineage, with an accumulation of early myeloid progenitors and concurrent loss of LSKs, LMPs, lymphoid and DC progenitor cells, as well as monocytes. This suggests a lineage specific effect of the APEX1 REF-1 function. In line with this notion is the observation that murine BM cells treated with E3330 produced fewer myeloid progenitors, GMPs, and erythroid progenitors in colony forming assays [98]. Likewise, E3330 treatment caused adult and embryonic stem cells to differentiate towards some but not all neuronal sub-types [99].

Our transcriptomics data identified APEX1 redox function to be key for regulating IRGs in expanding HSPCs. Variable effects of REF-1 inhibition on IFN signaling in other systems have been reported. E3330 was shown to activate p38 MAPK signaling in antigen presenting cells and thereby modulating IFN-γ production in T cells [100]. By contrast, other studies demonstrated that REF-1 induces lipopolysaccharide-dependent pro-inflammatory cytokines in monocytic or macrophage cell lines in an NF-κB dependent manner [34, 35]. Similar to p38 MAPK, NF-κB is also known to control interferon signaling [79, 101].

In general, it is believed that high levels of IFN signaling impair in vitro and in vivo BM HSC self-renewal capacity [56, 79–81, 102, 103]. However, we here demonstrated that IFN signaling is also required for functional ex vivo expansion of adult murine HSCs. This is in line with what has been observed during development, where IFN signaling supports perinatal HSC and lymphoid progenitor development [82, 83], as well as during ex vivo human HSC culture using UM171, where pro-inflammatory NF-κB mediated signaling is required for true HSC expansion [104]. The skewing of differentiation towards the megakaryocyte lineage at the detriment of monocytic differentiation caused by REF-1 inhibition is consistent with studies that illustrated that IFN type I signaling repressed in vitro megakaryocyte differentiation, while being required for Ly6C^high^ monocyte differentiation [105, 106]. Our results also show that REF-1 inhibition does not impair HSPCs when cultured under conditions that induce much higher levels of inflammatory factors (albumin-containing SFEM medium); conditions associated with significantly less HSC maintenance compared to PVA-based culture that maintains functional HSCs [44]. Hence, the REF-1-mediated impairment of HSPC appears to be specific for culture conditions where inflammatory cytokines are low, and as a result repopulating HSCs are expanded. Partial rescue of the HSPC expansion defect (due to APX2009) was achieved by IFN-γ treatment. To achieve a complete rescue might require that cells are exposed to not only IFN-γ but to a balanced mixture of several inflammatory cytokines. Alternatively, the fact that HSCs, like other stem cells, intrinsically express IFN-independent IRGs and do poorly respond to exogenous IFN-β [107], might explain that the effect of REF-1 inhibition can only be partially rescued by exogeneous IFN-γ and not by IFN-α. Further studies will have to elucidate why IFN-γ only partially rescued the APX2009 treated HSPCs and not the E3330 ones. Moreover, it remains to be determined if the decreased IRG expression and reduced related TF activity in the HSPCs following REF-1 inhibition is due to specific HSPC-intrinsic IFN signaling reduction, or if it is caused by reduced extrinsic IFN/inflammatory signaling in the altered mature lineages of the culture.

In conclusion, our study highlights the importance of APEX1 during regenerative hematopoiesis. In addition, our data indicate differential roles for the APEX1 nuclease and REF-1 functions in HSPC expansion and maintenance. Domain specific KO mouse models could elucidate the function of both APEX1 domains during in vivo hematopoiesis. Such studies might also shed light on the role of APEX1 during steady-state hematopoiesis, where most adult HSCs are dormant. The APEX1 nuclease function plays predominately a role in HSPC survival and maintenance even if we do not yet fully understand this mechanism. By contrast, APEX1 REF-1 functions by regulating interferon transcriptional networks in HSPCs and their progeny, thereby supporting functional proliferation of HSPCs. Whether inhibition of the APEX1 REF-1 function blocks differentiation towards monocyte and DC/lymphoid lineages and favors MEP commitment, and/or whether some committed cells are more susceptible to die than others due to decreased IFN/inflammatory signaling, still needs further evaluation. Also, which TF(s) is (are) the upstream REF-1 target(s) in HSPCs, governing the IRG expression, remains to be determined. Finally, this study may aid in our understanding of potential hematopoietic side effects associated with the use of APEX1 inhibitors in cancer therapy and suggests a possible beneficial therapeutic use of E3330 in interferonopathies .

## Supplementary Information

Below is the link to the electronic supplementary material.

Supplementary file 1 (XLSX 722 KB) contains the top RNA cluster marker genes for each of the 10 defined CITE-seq cell identities.

Supplementary file 2 (XLSX 36 KB) contains the top ADT markers for each of the 10 defined CITE-seq cell identities.

Supplementary file 3 (XLSX 33 KB) contains the lists of genes used to calculate different enrichment scores.

Supplementary file 4 (XLSX 91 KB) contains the top Regulons identified by SCENIC for each of the 10 defined CITE-seq cell identities.

Supplementary file 5 (XLSX 3322 KB) contains DEG analysis between Inhibitor III/E3330/APX2009 and DMSO for each of the 10 defined CITE-seq cell identities.

Supplementary file 6 (XLSX 1437 KB) contains DAR analysis between Inhibitor III/E3330/APX2009 and DMSO for each of the 10 defined CITE-seq cell identities.

Supplementary file 7 (XLSX 36 KB) contains DEGs and DARs common to all 3 APEX1 inhibitors, and specific to the APEX1 nuclease or redox inhibition.

Supplementary file 8 (XLSX 163 KB) contains the results of differential regulon activity analysis along pseudotime for the 4 distinct differentiation trajectories between the Inhibitor III and DMSO treatments.

Supplementary file 9 (DOCX 15.3 MB) contains all the Supplementary Methods, Tables and Figures.

## Data Availability

CITE-seq data can be accessed under GEO:GSE218981. Other data from this study are available from the corresponding author upon reasonable request.
