# Supplementary material for: APEX1 Nuclease and Redox Functions are Both Essential for Adult Mouse Hematopoietic Stem and Progenitor Cells
Source: Stem Cell Rev Rep. 2023 Jun 2;19(6):2052–72. doi: 10.1007/s12015-023-10550-0 (PMC10390635; doi:10.1007/s12015-023-10550-0)
Supplement: Supplementary file 9 — (DOCX 15.3 MB) contains all the Supplementary Methods, Tables and Figures. [file 12015_2023_10550_MOESM9_ESM.docx]

**Supplementary Methods, Tables and Figures**

**APEX1 nuclease and redox functions are both essential for adult mouse hematopoietic stem and progenitor cells.**

Samantha Zaunz^1^, Jonathan De Smedt^1,2^, Lukas Lauwereins^1^, Lana Cleuren^1^, Charlie Laffeber^3^, Manmohan Bajaj^1^, Joyce H. G. Lebbink^3,4^, Jurgen A. Marteijn^3^, Kim De Keersmaecker^5^, Catherine Verfaillie^1^.

**Authors affiliations:**

^1^ Stem Cell Institute, Department of Development and Regeneration, KU Leuven, Leuven, Belgium;

^2^ Current address: GlaxoSmithKline Biologicals SA, 1300 Wavre, Belgium;

^3^ Department of Molecular Genetics, Oncode Institute, Erasmus MC Cancer Institute, Erasmus University Medical Center, Rotterdam, Netherlands;

^4^ Department of Radiotherapy, Erasmus MC Cancer Institute, Erasmus University Medical Center, Rotterdam, The Netherlands;

^5^ Laboratory for Disease Mechanisms in Cancer, Department of Oncology, KU Leuven, Leuven, Belgium.

**Corresponding author:** Samantha Zaunz

Email: samantha.zaunz@kuleuven.be, zaunzsamantha@gmail.com Phone: +32 16 37 72 83

Address: Stem Cell Institute of the KU Leuven, O&N IV Herestraat 49, 3000 Leuven, Belgium.

**Supplementary Methods**

**Lentiviral vector production**

Lentiviral particles were produced in low passage HEK293T cells (Clontech) using a selected sgRNA lentiguide GFP^high^ plasmid, the 2^nd^ generation packaging and envelope plasmids psPAX2 (Addgene, cat#12260) and pMD2.G (Addgene, cat#12259), and the transfection reagent FuGENE® HD (Promega) in Opti-MEM medium (Gibco). Transfection medium was changed after approximately 10-12 hours and replaced by DMEM high glucose Glutamax medium (Gibco), supplemented with 10% fetal bovine serum (Sigma-Aldrich), 1% Penicillin-Streptomycin, 1% sodium pyruvate (Gibco). 48h post-transfection, the medium was collected and filtered (0.45µm filter, Millipore). Lentiviral particles were concentrated using the Vivaspin20 tube concentrators (Sartorius), before being aliquoted and stored at -80°C. Most viral vector batches were titrated on fresh BM derived Lin^-^ cells, to determine the volume of concentrated viral particles needed to reach maximum transduction efficiency, before the toxicity related drop in transduction efficacy.

**Lentiviral transduction of HSPCs**

Magnetic-activated cell sorting (MACS) purified Lin^-^cKit^+^ cells from Cas9 and WT CD45.2 mice were seeded at 8×10^4^ cells/well in a round-bottom 96-well plate coated with fibronectin, using 200μl/well of SFEM medium (Stem Cell Technologies) supplemented with 100 ng/mL thrombopoietin TPO, 50 ng/mL stem cell factor (SCF) and 1% Penicillin-Streptomycin. The HSPCs were transduced by spinoculation (90 minutes at 500 g), using concentrated lentiviral particles (batch average of ± 13.6 μl/well) and 8 μg/mL of polybrene (Millipore). 12 and 18 hours post-transduction, a half-medium change was performed. 36-48 hours post-transduction, cells were used for transplantation and/or long-term *ex vivo* culture in PVA-based medium (as described in the methods section of the article).

**Bone marrow histology**

Tibia of some mice transplanted with APEX1 KO and WT cells, after 5 months post-transplantation, was processed for paraffin embedding and hematoxylin-eosin staining, as described in ^[1]^. Brightfield microscope pictures were taken with the ZEISS Axio Imager Z1/Apotome microscope and AxioCam MRC5 (40X objective).

**1-year post-transplantation follow-up experiment**

For the 1-year transplantation experiments, white blood cell (WBC) counts were obtained using the Scil Vet abc device, and the GFP^high^ percentage within the CD45.2^+^ transplanted cells was assessed by flow cytometry at different timepoints after transplantation. 2 out of the total of 26 transplanted recipients were discarded from the analysis as they died shortly after transplantation due to low repopulation and/or due to irradiation toxicity. 2 mice were sacrificed at 3 and 6 months after transplantation due to development of dermatitis, and their BM chimerism was analyzed (Figure S4C). 1 year after transplantation, BM was analyzed by flow cytometry.

**Flow cytometry**

All the staining steps described below were performed at 4°C for 30 min in FACS buffer (phosphate-buffered saline (PBS) with 0.1% bovine serum albumin (BSA)) and cells were washed with FACS buffer after each staining step, unless otherwise specified. For the peripheral blood and the bone marrow analysis after transplantation, the erythrocytes were lysed for 10 min in red blood cell lysis solution (BioLegend) before processing to flow cytometry staining. Detailed antibody and dilution information can be found in Table S4.

Peripheral blood cells were stained with anti-mouse CD3e APC, anti-mouse/human CD11b PE, anti-mouse Ly-6G/Ly-6C PE, anti-mouse CD45R PE-Cyanine7, anti-mouse CD45.1 Brilliant Violet 605 or anti-mouse CD45.1 APC-Cyanine7, and anti-mouse CD45.2 eFluor 450.

The same panel of antibodies was used to analyze the BM cells after transplantation. Post-transplantation BM cells were also stained with a second panel, including a primary anti-mouse lineage biotin antibody cocktail and secondary antibodies. The latter included anti-mouse CD45.1 Brilliant Violet 605, anti-mouse CD45.2 eFluor 450, streptavidin eFluor710, anti-mouse c-Kit APC, anti-mouse Sca-1 PE-Cyanine7, anti-mouse CD150 PE and anti-mouse CD48 APC-eFluor 780. Mice with a CD45.2 repopulation percentage below 5% were excluded from the 5 months post-transplantation BM analysis. GFP^high^ % within the LSK-SLAM population was not quantified when the number of total events in that specific population was less than 10 events.

For the CRISPR-Cas9 and APEX1 inhibitor treated HSPC expansion experiments, cells were first stained with an anti-mouse lineage biotin cocktail. Next, secondary staining was performed in AnnexinV binding buffer (140mM NaCl solution with 2.5mM CaCl_2_ and 0.01M HEPES) using streptavidin eFluor710, anti-mouse c-Kit PE-Cyanine7, anti-mouse Sca-1 BV605, anti-mouse CD150 PE, anti-mouse CD48 eFluor450, AnnexinV-APC and viability Dye-eFluor780.

For the cell division quantification, freshly MACS purified Lin^-^cKit^+^ cells were labelled using the CellTrace^TM^ Violet or CellTrace^TM^ CFSE (Invitrogen) proliferation dye according to the supplier’s recommendation. Briefly, cells were incubated with 5µM per 10^6^ of cells, diluted in prewarmed DPBS with calcium and magnesium (Gibco), for 20 min at 37°C. Next, at least three times the staining volume of DPBS with 1% BSA (Miltenyi) was added to the cells and incubated at 37°C for 5 min. Finally, cells were washed with DPBS before being plated for *ex vivo* culture or stained. For flow cytometry after culture, CellTrace^TM^ Violet or CellTrace^TM^ CFSE labelled cells were first stained with anti-mouse lineage biotin cocktail. After the primary staining, cells were labelled with the viability Dye-eFluor780, streptavidin eFluor710, anti-mouse c-Kit APC, anti-mouse Sca-1 PE-Cyanine7, anti-mouse CD150 PE, anti-mouse CD48 FITC or eFluor450.

For IFN-exposed cultures, cells were first stained with anti-mouse lineage biotin cocktail. Secondary staining included : viability Dye-eFluor780, streptavidin eFluor710, anti-mouse CD34 FITC, anti-mouse CD201 (EPCR) APC, anti-mouse Sca-1 BV605, anti-mouse c-Kit PE-Cyanine7, anti-mouse CD150 PE and anti-mouse CD48 eFluor450. In this experiment, following the IFN treatment, functional hematopoietic stem cells were defined as Lin^-^cKit^+^Sca^+^CD150^+^CD48^-^ (LSK-SLAM) cells that were also CD201^+^CD34^-^(Figure S17), such as described in ^[2]^.

For combination treatment experiments, cells were first stained with anti-mouse lineage biotin cocktail. Secondary staining included : viability Dye-eFluor780, streptavidin eFluor710, anti-mouse CD201 (EPCR) APC, anti-mouse Sca-1 Super Bright 436, anti-mouse c-Kit PE-Cyanine7, anti-mouse CD150 PE and anti-mouse CD48 FITC.

For stainings on the expanded HSPC progenies, 10 000 counting beads (Invitrogen) were added to each sample before acquisition, to quantify the cell expansion (absolute number of cells/well). For the APEX1 inhibitor experiments, cell expansion calculations were based on AnnexinV and CellTrace^TM^ stainings. Flow cytometry data were acquired on the BD FACS Symphony or the BD FACS Canto II (Becton Dickinson), and analyzed using FlowJo V10 software. Single stained cells were used for setting up compensation matrix and Fluorescence Minus One (FMO) controls were included to facilitate cell gating when needed.

Figure S16 and S17 show gating strategies that were employed.

**Fluorescence-activated Cell Sorting (FACS)**

For off-target analysis, nanocapillary immunoassay and AP incision assay, total GFP^high^ cells were sorted from 2-week cultured progeny cells of the transduced Lin^-^cKit^+^ cells (WT and Cas9).

For the viable Lin^-^cKit^+^GFP^high^ (sgRNA transduced WT and Cas9 cells) sorts, cells were stained with viability Dye-eFluor 780, anti-mouse lineage APC antibody cocktail, anti-mouse c-Kit PE and anti-mouse Sca-1 SuperBright 436. For the viable Lin^-^cKit^+^ sorts from the inhibitor treated samples, cells were stained with viability Dye-eFluor 780, anti-mouse lineage FITC antibody cocktail, anti-mouse c-Kit APC and anti-mouse Sca-1 PeCyanine7.

For the freshly isolated LSK cell sort, BM MACS isolated Lin^-^ were stained for the anti-lineage FITC antibody cocktail, anti-mouse c-Kit APC, anti-mouse Sca-1 PeCyanine7, anti-mouse CD150 PE, anti-mouse CD48 eFluor450 and the viability Dye-eFluor 780.

Before running the 10X Genomics CITE-seq, LSK progeny for each treatment condition was sorted for live cells using viability Dye-eFluor 780.

All the sorts were performed on the MA9000 cell sorter (Sony) or on the BD Aria III (Becton Dickinson). Figure S18 shows gating strategies that were employed for the sorts.

**DNA extraction and off-targets analysis**

Genomic DNA was extracted from sorted GFP^high^ cell pellets, using the Gentra Puregene Tissue Kit (Qiagen), according to the manufacturer’s protocol. Polymerase chain reactions (PCRs) were carried out, using the Q5 High-Fidelity DNA Polymerase (New England Biolab) following the manufacturer’s protocol, to amplify the sgRNA-targeted (on-targets) and untargeted genomic regions (off-targets) (Table S2). Forward and reverse primers used for these amplifications are listed in Table S3. After confirming the expected product size by gel electrophoresis, the PCR products were purified using the PureLink PCR Purification Kit (Invitrogen) prior to Sanger Sequencing (Eurofins Genomics). For each sgRNA, the top 7 off-target regions, predicted using the *in silico* CRISPR-Cas9 guide RNA design checker tool (Integrated DNA Technologies), were sequenced. To determine the indel (small insertions and deletions introduced by Cas9 editing) percentage, the sequenced amplicons were aligned against the non-edited control amplicons using the ICE (Interference of CRISPR Edits) tool from Synthego.

**Nanocapillary immunoassay**

GFP^high^ progeny of transduced Cas9 or WT Lin^-^cKit^+^ cells was FACS-sorted 2 weeks post-transduction. Cell pellets were lysed in RIPA buffer (Sigma-Aldrich) supplemented with EDTA-free protease and phosphatase cocktail inhibitors (Roche) for 30 min, with occasional vortexing. Protein supernatants were collected after a 20 min centrifugation at 4°C and quantified using the Pierce^TM^ BCA Protein Assay kit (ThermoFisher Scientific). For the WES (Automated Western Blots with Simple Western; ProteinSimple) protein detection, samples were loaded on a 25-well assay plate at a concentration of 0.2µg/µl, together with a 12-230 kD standard ladder, following the manufacturer’s recommendations. Anti-APEX1 (Abcam, cat#189474) and anti-VINCULIN (Cell Signaling Technology, cat#13901T) antibodies were used at 1/20 and 1/10,000, respectively. Data were analyzed using the Compass Software (ProteinSimple).

**Apurinic/apyrimidinic (AP) incision assays**

Protein extractions for the AP incision assay were performed according to the instructions and using the extraction ‘Buffer A’ described in ^[3]^. Prior to the extraction, the total day 7 progeny of Lin^-^cKit^+^ cells treated with the APEX1 inhibitors, or the sorted total GFP^high^ cells from transduced Cas9 and WT (after 2 weeks of culture) cells, were briefly washed with PBS, lysed and snap-frozen in Buffer A.

The 5’- CAA TAA TAA CAC GC/idSp/ CGA CCA GTC CTG CTT TTG CAG GAC TGG TCG CGC GTG TTA TTA TTG /3Cy5Sp/-3’ (Integrated DNA Technologies) fluorescently labelled hairpin oligonucleotide, containing an AP site analogue (idSp), was self-annealed by incubation at 95°C for 5 min, followed by slow cooling down to room temperature (RT) in 25mM HEPES (pH 7.5) with 150mM KCl. NEB4 buffer (50 mM KAc, 20mM TRIS-Ac, 10mM Mg-Ac, 1mM DTT) supplemented with 0.1 mg/ml of BSA and 0.5% of PVA was used as reaction buffer for the assay, and to dilute the protein extracts. For the APEX1 inhibitor treated samples, the same concentration of inhibitor, as used during culture, was added to the protein extracts and the final reaction volume due to the reversible nature of the inhibitors^[4–7]^. Before mixing with the annealed oligonucleotide, the protein extracts were pre-heated at 37°C for 5 min (APEX1 KO and WT samples) or for 1h (for the inhibitor treated samples). The AP incision reactions were carried out at a final concentration of 10ng/µl protein extracts and 10µM double-stranded hairpin DNA, for 5 min at 37°C. To stop the enzymatic nicking, an equal volume of urea solution (8M urea with 0.3% SDS) was added to the reactions at RT, followed by 3 min formamide (Honeywell Riedel-de Haën) denaturation at 70°C. Denatured samples were loaded on a denaturing 15% acrylamide (19:1 acryl:bis) 8M urea-TBE (tris/borate/EDTA buffer) PAGE gel to separate cleaved product from un-cleaved oligonucleotide substrate. The fluorescent signal on the gels was analyzed using a Typhoon™ imager (Cytiva). The Cy5 fluorophore was excited at 635nm and emission was passed through the Cy5 670BP30 filter. Band intensities were quantified using Image J software^[8]^. Purified APEX1 enzyme (NEB) at final concentration of 2.5 units/ml was included as a positive control in the assay.

**Apurinic/apyrimidinic (AP) site quantification**

To quantify AP sites, the aldehyde reactive probe-based DNA Damage Assay kit ab211154 (Abcam) was used on genomic DNA of total culture or sorted cells, according to the manufacturer’s instructions. Absorbance was measured using a GloMax® Discover Microplate Reader (Promega).

**Phospho-gamma H2AX staining**

Approximately 50 000 sorted cells were seeded onto a CellView slide (Greiner) using CellTak™ Adhesive glue (Corning). Sorted Lin^-^cKit^+^ cells were allowed to attach for 20-30 min at 4°C. After cell attachment, samples were fixed using 4% paraformaldehyde for 15 min at RT. Cells were then blocked and permeabilized for 1 hour at RT with a 5% normal donkey serum (Jackson ImmunoResearch) and 0.3% Triton X-100 PBS solution, before being stained for 2 hours at RT with anti-phospho-γH2AX (Cell Signaling Technology, cat#9718), diluted 1/200 in 0.5% BSA 0.3% Triton X-100 PBS solution. After PBS washes, cells were stained for 1 hour at RT with 1/500 secondary donkey anti-rabbit antibody Alexa 647 (Invitrogen, cat#A31573) and 1/50,000 Hoechst33342 (Thermo Scientific, cat#62249) diluted in DAKO REAL^TM^ Antibody Diluent solution (Agilent). After washing, coverslips were mounted in prolong gold anti-fade reagent (Invitrogen). Confocal images were taken with the Zeiss LSM880 at RT, with a plan-apochromat 40X/1.3 oil objective and with a pinhole opening of 76µM for both channels. Cell Profiler software was used for imaging analysis. Percentage of cells presenting at least 1 γH2AX focus pernucleus was calculated. Pan-nuclear γH2AX positive cells were excluded from analysis.

**Cytokine quantification**

For the cytokine quantification present in culture media, Lin^-^cKit^+^ cells were seeded at 5x10^4^ cells/100µl of PVA-based or SFEM medium (Table S1) in a 96-well plate. After 3 and 7 days, the supernatant was analyzed using the mouse anti-virus response LEGENDplex kit (Biolegend), including IFN-γ, KC, TNF-α, MCP-1, IL-12p70, RANTES, IL-1β, IP-10, GM-CSF, IL-10, IFN-β, IFN-α, IL-6. The 13 multi-analyte flow assay kit was used according to the manufacturer’s protocol. Analytes that could not be detected in both culture media were excluded in the figure; IL-12p70, GM-CSF, IL-10, IFN-α on day3 and TNF-α, IL-12p70, IFN-α on day 7.

**10X Genomics CITE-sequencing**

An average of 4x10^4^ sorted LSKs (from 3 donor sets, sorted LSK cells contained 5.50±0.04 % LSK-SLAM) were plated per well in fibronectin-coated 48 well plates and cultured in PVA-medium with APEX1 inhibitors or DMSO for 7 days, as described above. 3 biological culture replicates for each condition were pooled before cell staining with hashtag oligos (HTO) and antibody derived tag (ADT).

For CITE-seq labelling, about 1 to 2 million cells were counted, isolated and spun down. The cell pellet was resuspended and incubated for 30 min on ice with 25µl of staining mix in PBS containing 0.04% BSA, True-Stain Monocyte Blocker™ (BioLegend, 426101), TruStain FcX Block (BioLegend, cat#101320), viability dye eFluor™ 780 (Invitrogen, 1/2000), and the mouse cell surface protein antibody panel containing 158 oligo-conjugated antibodies (TotalSeq™-A, BioLegend) and 10 TotalSeq-A isotype controls (see Table S5) according to the manufacturer's instructions and as previously described^[9]^. 4 different samples (DMSO, E3330, APX2009, Inh. III) were tagged using TotalSeq-A hashtag antibodies (anti-mouse CD45 and MHC class I A0311 (cat#155821), A0312 (cat#155823), A0313 (cat#155825) and A0314 (cat#155827) respectively). After the viability sort (Figure S18B), the single-cell suspensions were pooled together and resuspended at an estimated final concentration of 1000 cells/µl and loaded on a Chromium GemCode Single Cell Instrument (10X Genomics) to generate single-cell gel beads-in-emulsion (GEM). The scRNA-Seq libraries were prepared using the GemCode Single Cell 3’ Gel Bead and Library kit, version NextGEM 3.1 (10X Genomics) according to the manufacturer’s instructions with the addition of amplification primers (3nM, 5’CCTTGGCACCCGAGAATT*C*C and 5’GTGACTGGAGTTCAGACGTGTGC*T*C) during cDNA amplification to enrich the TotalSeq-A cell surface protein and HTOs. Size selection with SPRIselect Reagent Kit (Beckman Coulter, cat#B23318) was used to separate amplified cDNA molecules for 3’ gene expression and cell surface protein construction. TotalSeq-A protein library construction including sample index PCR using Illumina’s Truseq Small RNA primer sets and SPRIselect size selection was performed according to the manufacturer’s instructions. The cDNA content of pre-fragmentation and post-sample index PCR samples was analyzed using the 2100 BioAnalyzer (Agilent). Sequencing libraries were loaded on an Illumina NovaSeq flow cell at VIB Nucleomics core with sequencing settings according to the recommendations of 10X Genomics, pooled in a 80/25 ratio for the combined 3’ gene expression and cell surface protein samples, respectively.

The Cell Ranger pipeline (10X Genomics, version 6.1.2) was used to perform sample demultiplexing and to generate FASTQ files for read 1, read 2 and the i7 sample index for the gene expression and cell surface protein libraries. Read 2 of the gene expression libraries was mapped to the reference genome (mouse GRCm38) using Cell Ranger. Filtered count matrices for RNA, HTO and ADT expression generated by Cell Ranger pipeline, were used for downstream analysis.

**CITE-seq analysis**

For the RNA matrix, only genes that were expressed in at least 3 cells, were kept for analysis. Outlier cells were identified, by a multivariate approach based on all quality metrics (number of UMIs, genes detected, and percentage of mitochondrial RNA), using the R package *scater*^[10]^. *MULTIseqDemux()* function in *Seurat* (version 4.1.0)^[11]^ was used to demultiplex centered log-ration (CLR) normalized HTO matrix, in order to annotate each cell to their experimental group or identify the cell as doublet/negative cell. Additional filtering of low-quality cells was performed after demultiplexing (percentage of mitochondrial genes/cells >4% and number of detected genes/cell <1,200) (Figures S6A), and doublet or negative (not hashed) cells were removed. A total of 2718 cells for the DMSO, 1938 cells for the Inh. III, 2641 cells for the E3330 and 1704 cells for APX2009 treatment conditions were analyzed, with a median sequencing depth of 2815.9 genes per cell for the 4 experimental conditions.

RNA data were SCT-transformed and simultaneously the cell cycle effect (S phase and G2M phase scores determined according to ^[12]^) was regressed out. SCT-normalized and cell cycle corrected RNA data was used for all the downstream analysis. Principal component analysis (PCA) dimensional reduction was performed using the default *Seurat* setting, and the first 25 principal components (PCs) were used for clustering the cells with a resolution of 0.7, resulting in 10 cell identities on the Uniform Manifold Approximation and Projection (UMAP). ADT data were CLR normalized across the cells and scaled. For the flow cytometry like gating, the raw ADT was normalized with the *DSB* (denoised and scaled by background) package ^[13]^, by defining empty droplet background (cut-off protein library size < 1.5 and cut-off RNA library size < 2.7), using isotype controls (IgG2a-Mouse-k, IgG-Hamster, IgG2b-Mouse-k, IgG2b-Rat-k, IgG2a-Rat-k, IgG1-Mouse-k, IgG2c-Rat-k, IgG1-Rat-k, IgD) and the default settings. Proteins with maximum count ≤ 1 per cell were removed. After the DSB normalization, CITE-seq protein data were used to define the different HSPC populations using different flow cytometry R packages (flowWorkspace, ggcyto, flowSpecs, flowcore) (Figure S10C-D).

Cluster annotation was done using the expression of selected marker genes for HSC, lymphoid, megakaryo-erythroid and myeloid lineages^[14–18]^ (Figure 4D and Figure S9B), as well as using the top cluster marker genes (Figure S9A, Supplement File 1), the top ADT marker (Figure S10, Supplement File 2), and the top regulons for each cluster identified by SCENIC (Figure S9C, Supplement File 4). HSC cluster 0 and LSK cluster 3 identity annotations were further confirmed using previously published LSK and HSC transcriptomic signatures from bulk RNAseq^[19,20]^ (Figure 4C). Stressed cell cluster 1 and 10 showed higher mitochondria gene percentage per cell compared to all other clusters (Figure S8C).

The top RNA and the top ADT markers (CLR normalized) per cluster (Supplement File 1 and 2 respectively) were detected with the *FindAllMarkers* function in Seurat (for RNA data with logfc.threshold=0.1, min.diff.pct=0.05, only.pos=T; for ADT data, min.diff.pct = 0.01 with logfc.threshold = 0, only.pos=T). Enrichment score for LSK^[19]^, HSC^[20]^ and functional HSC (MolO signature)^[21]^ (Supplement File 3) gene signatures were calculated using the Seurat *AddModuleScore* function (default settings). Single-sample gene set enrichment analysis (ssGSEA)^[22]^ was performed on individual clusters, using *escape* R package with default settings and defined gene sets (GO:0034340 and GO:0034341 for interferon type I and II; megakaryocyte lineage-primed markers from ^[23]^, Supplement File 3). For each cluster, Dunn’s test with Benjamini-Hochberg correction was used to compare the ssGSEA scores of the different inhibitor samples to DMSO.

The SCENIC workflow^[24]^ was used to identify active regulons, which are TFs and their co-regulated target genes, and to calculate cell-specific AUC score for each identified regulon in the data set. Prior to the *GRNBoost2* GRN interference, SCT-normalized RNA count matrix was filtered for genes that are also present in the SCENIC mouse databases (500bp-upstream and centered-10kb databases of mm9-refseq_r45-mc9nr were used). Only positive correlations between targets and TFs were detected. 520 active regulons were identified in our dataset. The top active regulons for each of the 10 cell identities were selected based on their positive mean regulon activity score (Z-score) computed within cluster of interest (Supplement File 4).

For each inhibitor, we performed a chi-square test (with a Benjamini-Hochberg correction for multiple testing) to assess if cluster proportions (RNA and ADT) differed between DMSO control and inhibitor-treated populations. Additionally, log2 fold changes between observed versus expected cluster proportions was calculated.

**Differential signature analysis**

The *Seurat FindMarkers* function (logfc.threshold=0.1,min.diff.pct=0.05, group.by="Sample") was applied on each cluster to identify the DEGs (Supplement File 5). The DARs for each cluster were identified based on the SCENIC regulon AUC scores for all 520 identified regulons, using the Wilcoxon test with a Benjamini-Hochberg correction (Supplement File 6). Both DEG and DAR analyses compared each the inhibitor-treated samples with the DMSO condition. For the heatmaps summarizing the DARs per cluster, the mean activity score for all regulons were first scaled across the cluster of interest.

From all these DEG and DAR analyses, common (DEGs or DARs present in all 3 inhibitors) and specific changes for the nuclease inhibition (only for Inh. III, and not for E3330 and/or APX2009) and the 2 redox inhibitions (common to E3330 and APX2009, and not present for Inh. III) were highlighted for each cluster (adj. p-value<0.05) (Supplement File 7). The heatmaps for “stressed” cluster 1 and 10, were excluded from Figure 5 and Figure S11-12, as these clusters did not change massively upon APEX1 inhibitor treatment.

Cytoscape (version 3.9.1) was used to visualize the dysregulated regulons of the HSC cluster 0 and the LSK cluster 3.

**Trajectory inference**

For the trajectory inference, 4 distinct differentiation trajectories (megakaryocyte-erythroid progenitor (MEP), granulocyte-monocyte progenitor (GMP), monocyte and dendritic cell/lymphoid progenitor (DCP/LP)) were defined for the DMSO and Inh. III samples. Therefore, clusters of interest for each trajectory were subsetted, and UMAP coordinates were calculated on each subset using *Seurat*. Cluster 0, 2, 3, and 5 were used for the MEP trajectory; cluster 0, 2, 3, and 4 for GMP trajectory; cluster 0, 3, 6, and 8 for the monocyte trajectory; and cluster 0, 3, and 8 for the DCP/LP trajectory. Subsequently, *Slingshot*^[25]^ trajectories were inferred for each of the 4 subsets, with cluster 0 as root population. Next, cells were binned (15 bins) and mixed ANOVA tests with Benjamini-Hochberg correction were used to identify differential regulon activity along the pseudotime axis (based on AUC score), comparing DMSO and Inh. III treatments. Regulons were differentially active between DMSO and Inh. III if the magnitude of activity was consistently different (adj. p-value sample) and if the activity trends differed between treatments (adj. p-value interaction) (Supplement File 8).

**References**

1. Mesnieres, M., Böhm, A.-M., Peredo, N., Trompet, D., Valle-Tenney, R., Bajaj, M., Corthout, N., Nefyodova, E., Cardoen, R., Baatsen, P., Munck, S., Nagy, A., Haigh, J. J., Khurana, S., Verfaillie, C. M., & Maes, C. (2021). Fetal hematopoietic stem cell homing is controlled by VEGF regulating the integrity and oxidative status of the stromal-vascular bone marrow niches. *Cell Reports*, *36*(8), 109618. https://doi.org/10.1016/j.celrep.2021.109618

2. Rabe, J. L., Hernandez, G., Chavez, J. S., Mills, T. S., Nerlov, C., & Pietras, E. M. (2020). CD34 and EPCR coordinately enrich functional murine hematopoietic stem cells under normal and inflammatory conditions. *Experimental Hematology*, *81*, 1-15.e6. https://doi.org/10.1016/j.exphem.2019.12.003

3. Vodenkova, S., Azqueta, A., Collins, A., Dusinska, M., Gaivão, I., Møller, P., Opattova, A., Vodicka, P., Godschalk, R. W. L., & Langie, S. A. S. (2020). An optimized comet-based in vitro DNA repair assay to assess base and nucleotide excision repair activity. *Nature Protocols*, *15*(12), 3844–3878. https://doi.org/10.1038/s41596-020-0401-x

4. Rai, G., Vyjayanti, V. N., Dorjsuren, D., Simeonov, A., Jadhav, A., Wilson, D. M., & Maloney, D. J. (2012). Synthesis, Biological Evaluation, and Structure–Activity Relationships of a Novel Class of Apurinic/Apyrimidinic Endonuclease 1 Inhibitors. *Journal of Medicinal Chemistry*, *55*(7), 3101–3112. https://doi.org/10.1021/jm201537d

5. Kelley, M. R., Wikel, J. H., Guo, C., Pollok, K. E., Bailey, B. J., Wireman, R., Fishel, M. L., & Vasko, M. R. (2016). Identification and Characterization of New Chemical Entities Targeting Apurinic/Apyrimidinic Endonuclease 1 for the Prevention of Chemotherapy-Induced Peripheral Neuropathy. *Journal of Pharmacology and Experimental Therapeutics*, *359*(2), 300–309. https://doi.org/10.1124/jpet.116.235283

6. Shimizu, N., Sugimoto, K., Tang, J., Nishi, T., Sato, I., Hiramoto, M., Aizawa, S., Hatakeyama, M., Ohba, R., Hatori, H., Yoshikawa, T., Suzuki, F., Oomori, A., Tanaka, H., Kawaguchi, H., Watanabe, H., & Handa, H. (2000). High-performance affinity beads for identifying drug receptors. *Nature Biotechnology*, *18*(8), 877–881. https://doi.org/10.1038/78496

7. Kelley, M. R., Luo, M., Reed, A., Su, D., Delaplane, S., Borch, R. F., Nyland, R. L., Gross, M. L., & Georgiadis, M. M. (2011). Functional Analysis of Novel Analogues of E3330 That Block the Redox Signaling Activity of the Multifunctional AP Endonuclease/Redox Signaling Enzyme APE1/Ref-1. *Antioxidants & Redox Signaling*, *14*(8), 1387–1401. https://doi.org/10.1089/ars.2010.3410

8. Schindelin, J., Arganda-Carreras, I., Frise, E., Kaynig, V., Longair, M., Pietzsch, T., Preibisch, S., Rueden, C., Saalfeld, S., Schmid, B., Tinevez, J.-Y., White, D. J., Hartenstein, V., Eliceiri, K., Tomancak, P., & Cardona, A. (2012). Fiji: An open-source platform for biological-image analysis. *Nature Methods*, *9*(7), 676–682. https://doi.org/10.1038/nmeth.2019

9. Scheyltjens, I., Van Hove, H., De Vlaminck, K., Kancheva, D., Bastos, J., Vara-Pérez, M., Pombo Antunes, A. R., Martens, L., Scott, C. L., Van Ginderachter, J. A., Saeys, Y., Guilliams, M., Vandamme, N., & Movahedi, K. (2022). Single-cell RNA and protein profiling of immune cells from the mouse brain and its border tissues. *Nature Protocols*, *17*(10), 2354–2388. https://doi.org/10.1038/s41596-022-00716-4

10. McCarthy, D. J., Campbell, K. R., Lun, A. T. L., & Wills, Q. F. (2017). Scater: Pre-processing, quality control, normalization and visualization of single-cell RNA-seq data in R. *Bioinformatics*, btw777. https://doi.org/10.1093/bioinformatics/btw777

11. Hao, Y., Hao, S., Andersen-Nissen, E., Mauck, W. M., Zheng, S., Butler, A., Lee, M. J., Wilk, A. J., Darby, C., Zager, M., Hoffman, P., Stoeckius, M., Papalexi, E., Mimitou, E. P., Jain, J., Srivastava, A., Stuart, T., Fleming, L. M., Yeung, B., … Satija, R. (2021). Integrated analysis of multimodal single-cell data. *Cell*, *184*(13), 3573-3587.e29. https://doi.org/10.1016/j.cell.2021.04.048

12. Tirosh, I., Izar, B., Prakadan, S. M., Wadsworth, M. H., Treacy, D., Trombetta, J. J., Rotem, A., Rodman, C., Lian, C., Murphy, G., Fallahi-Sichani, M., Dutton-Regester, K., Lin, J.-R., Cohen, O., Shah, P., Lu, D., Genshaft, A. S., Hughes, T. K., Ziegler, C. G. K., … Garraway, L. A. (2016). Dissecting the multicellular ecosystem of metastatic melanoma by single-cell RNA-seq. *Science*, *352*(6282), 189–196. https://doi.org/10.1126/science.aad0501

13. Mulè, M. P., Martins, A. J., & Tsang, J. S. (2022). Normalizing and denoising protein expression data from droplet-based single cell profiling. *Nature Communications*, *13*(1), 2099. https://doi.org/10.1038/s41467-022-29356-8

14. Nestorowa, S., Hamey, F. K., Pijuan Sala, B., Diamanti, E., Shepherd, M., Laurenti, E., Wilson, N. K., Kent, D. G., & Göttgens, B. (2016). A single-cell resolution map of mouse hematopoietic stem and progenitor cell differentiation. *Blood*, *128*(8), e20–e31. https://doi.org/10.1182/blood-2016-05-716480

15. Weinreb, C., Rodriguez-Fraticelli, A., Camargo, F. D., & Klein, A. M. (2020). Lineage tracing on transcriptional landscapes links state to fate during differentiation. *Science*, *367*(6479), eaaw3381. https://doi.org/10.1126/science.aaw3381

16. Baccin, C., Al-Sabah, J., Velten, L., Helbling, P. M., Grünschläger, F., Hernández-Malmierca, P., Nombela-Arrieta, C., Steinmetz, L. M., Trumpp, A., & Haas, S. (2020). Combined single-cell and spatial transcriptomics reveal the molecular, cellular and spatial bone marrow niche organization. *Nature Cell Biology*, *22*(1), 38–48. https://doi.org/10.1038/s41556-019-0439-6

17. Drissen, R., Buza-Vidas, N., Woll, P., Thongjuea, S., Gambardella, A., Giustacchini, A., Mancini, E., Zriwil, A., Lutteropp, M., Grover, A., Mead, A., Sitnicka, E., Jacobsen, S. E. W., & Nerlov, C. (2016). Distinct myeloid progenitor–differentiation pathways identified through single-cell RNA sequencing. *Nature Immunology*, *17*(6), 666–676. https://doi.org/10.1038/ni.3412

18. Paul, F., Arkin, Y., Giladi, A., Jaitin, D. A., Kenigsberg, E., Keren-Shaul, H., Winter, D., Lara-Astiaso, D., Gury, M., Weiner, A., David, E., Cohen, N., Lauridsen, F. K. B., Haas, S., Schlitzer, A., Mildner, A., Ginhoux, F., Jung, S., Trumpp, A., … Amit, I. (2015). Transcriptional Heterogeneity and Lineage Commitment in Myeloid Progenitors. *Cell*, *163*(7), 1663–1677. https://doi.org/10.1016/j.cell.2015.11.013

19. Klimmeck, D., Cabezas-Wallscheid, N., Reyes, A., von Paleske, L., Renders, S., Hansson, J., Krijgsveld, J., Huber, W., & Trumpp, A. (2014). Transcriptome-wide Profiling and Posttranscriptional Analysis of Hematopoietic Stem/Progenitor Cell Differentiation toward Myeloid Commitment. *Stem Cell Reports*, *3*(5), 858–875. https://doi.org/10.1016/j.stemcr.2014.08.012

20. Sommerkamp, P., Romero-Mulero, M. C., Narr, A., Ladel, L., Hustin, L., Scho, K., Renders, S., Altamura, S., Zeisberger, P., Ja, K., Camargo, F. D., Perie, L., Trumpp, A., & Cabezas-Wallscheid, N. (2021). *Mouse multipotent progenitor 5 cells are located at the interphase between hematopoietic stem and progenitor cells*. 7. https://doi.org/10.1182/blood.2020007876

21. Wilson, N. K., Kent, D. G., Buettner, F., Shehata, M., Macaulay, I. C., Calero-Nieto, F. J., Sánchez Castillo, M., Oedekoven, C. A., Diamanti, E., Schulte, R., Ponting, C. P., Voet, T., Caldas, C., Stingl, J., Green, A. R., Theis, F. J., & Göttgens, B. (2015). Combined Single-Cell Functional and Gene Expression Analysis Resolves Heterogeneity within Stem Cell Populations. *Cell Stem Cell*, *16*(6), 712–724. https://doi.org/10.1016/j.stem.2015.04.004

22. Borcherding, N., Vishwakarma, A., Voigt, A. P., Bellizzi, A., Kaplan, J., Nepple, K., Salem, A. K., Jenkins, R. W., Zakharia, Y., & Zhang, W. (2021). Mapping the immune environment in clear cell renal carcinoma by single-cell genomics. *Communications Biology*, *4*(1), 122. https://doi.org/10.1038/s42003-020-01625-6

23. Rodriguez-Fraticelli, A. E., Wolock, S. L., Weinreb, C. S., Panero, R., Patel, S. H., Jankovic, M., Sun, J., Calogero, R. A., Klein, A. M., & Camargo, F. D. (2018). Clonal analysis of lineage fate in native haematopoiesis. *Nature*, *553*(7687), 212–216. https://doi.org/10.1038/nature25168

24. Aibar, S., González-Blas, C. B., Moerman, T., Huynh-Thu, V. A., Imrichova, H., Hulselmans, G., Rambow, F., Marine, J.-C., Geurts, P., Aerts, J., van den Oord, J., Atak, Z. K., Wouters, J., & Aerts, S. (2017). SCENIC: Single-cell regulatory network inference and clustering. *Nature Methods*, *14*(11), 1083–1086. https://doi.org/10.1038/nmeth.4463

25. Street, K., Risso, D., Fletcher, R. B., Das, D., Ngai, J., Yosef, N., Purdom, E., & Dudoit, S. (2018). Slingshot: Cell lineage and pseudotime inference for single-cell transcriptomics. *BMC Genomics*, *19*(1), 477. https://doi.org/10.1186/s12864-018-4772-0

26. Wilkinson, A. C., Ishida, R., Kikuchi, M., Sudo, K., Morita, M., Crisostomo, R. V., Yamamoto, R., Loh, K. M., Nakamura, Y., Watanabe, M., Nakauchi, H., & Yamazaki, S. (2019). Long-term ex vivo haematopoietic-stem-cell expansion allows nonconditioned transplantation. *Nature*, *571*(7763), 117–121. https://doi.org/10.1038/s41586-019-1244-x

**Supplementary Tables**

**Table S1:**  **Detailed composition of culture media.**

| **Component of PVA-based medium, based on** ^[26]^ | **Final concentration** | **Company** |
| --- | --- | --- |
| Ham's F-12 Nutrient Mix | - | Gibco |
| Polyvinyl alcohol (PVA) | 0.1% | Sigma-Aldrich (cat#P8136) |
| Penicillin-Streptomycin | 1% | Gibco |
| Insulin-Transferrin-Selenium (100X) | 1% | Gibco |
| HEPES (10mM) | 10 mM | Gibco |
| Recombinant murine thrombopoietin (TPO) | 100 ng/mL | PreproTech |
| Recombinant murine stem cell factor (SCF) | 20 ng/mL | PreproTech |

| **Component of SFEM medium** | **Final concentration** | **Company** |
| --- | --- | --- |
| StemSpan™ SFEM  which contains :   - Iscove’s MDM - Bovine serum albumin - Recombinant human insulin - Human transferrin (iron-saturated) - 2-Mercaptoethanol - Supplements | - | Stem Cell Technologies  (cat #09605) |
| Penicillin-Streptomycin | 1% | Gibco |
| Recombinant murine thrombopoietin (TPO) | 50 ng/mL | PreproTech |
| Recombinant murine stem cell factor (SCF) | 50 ng/mL | PreproTech |

**Table S2:**  **Predicted off-targets using the IDT *in silico* CRISPR-Cas9 guide RNA design checker tool, for both *Apex1* sgRNAs.**

| **sgRNA Name** | **Target** | **Sequence (5’-> 3’)** | **PAM** | **Score** | **Mismatches** | **Locus** | **Coding region/Comments** |
| --- | --- | --- | --- | --- | --- | --- | --- |
| sgRNA#1 *Apex1* | *Apex1* On Target | GACTGGAATACCGACAGCGT | TGG | N/A |  | chr14:+50926557 | *Apex1* exon 5 (on target score 52; off target 86) |
| sgRNA#1 *Apex1* | Off Target 1 | GAATGGAAT-CCGACAGCTT | GGG | 47 | 3 | chrX:-4609528, chrX:+34311861,  chrX:+3349722, chrX:-5110947,  chrX:-34545005 | Repeating Elements on chr.X |
| sgRNA#1 *Apex1* | Off Target 2 | GACTGGAATACTGACAGCAT | TGG | 57 | 2 | chr1:-45520188 |  |
| sgRNA#1 *Apex1* | Off Target 3 | GACTGGACTACTGACAACGA | AGG | 57 | 4 | chr2:-117024878 |  |
| sgRNA#1 *Apex1* | Off Target 4 | GACGGGAACACCGGCAGCCT | TGG | 61 | 4 | chr13:-51725518 | *Sema4d* |
| sgRNA#1 *Apex1* | Off Target 5 | GAGTAGAATACCAACAGCAT | AGG | 61 | 4 | chr8:+89949807 |  |
| sgRNA#1 *Apex1* | Off Target 6 | GGCTAGAATACAGACAGCCT | TGG | 62 | 4 | chr2:+15166864 |  |
| sgRNA#1 *Apex1* | Off Target 7 | GACTGGACTAC--ACAGCGT | AGG | 63 | 3 | chr19:+46096986 |  |
|  |  |  |  |  |  |  |  |
| sgRNA#2 *Apex1* | *Apex1* On Target | ACGGAGCTGACCAGTACTGA | TGG | N/A |  | chr14:-50926261 | *Apex1* Exon 4 (on target score 76; off target 75) |
| sgRNA#2 *Apex1* | Off Target 1 | TCAGAGATGACAAGTACTGA | AGG | 34 | 4 | chr6:-84772018 | *Exo6b* |
| sgRNA#2 *Apex1* | Off Target 2 | ACAGAGCTGACCA-TACTGA | AGG | 48 | 2 | chr8:-13590024 | *Rasa3* |
| sgRNA#2 *Apex1* | Off Target 3 | ACAGATCAGAACAGTACTGA | CGG | 50 | 4 | chr13:+98425086 | Proximity of *2310005E17Rik* |
| sgRNA#2 *Apex1* | Off Target 4 | ATGGAGCTGACTAGAACTGA | AGG | 58 | 3 | chr14:-15862994 | *Gm47782* |
| sgRNA#2 *Apex1* | Off Target 5 | ACAGATGAGACCAGTACTGA | CGG | 58 | 4 | chr16:+37754914 |  |
| sgRNA#2 *Apex1* | Off Target 6 | AAGAAGCTGGCCAGGACTGA | AGG | 59 | 4 | chr7:+6295296 | *Zfp667* |
| sgRNA#2 *Apex1* | Off Target 7 | AAGGAGATGACTTGTACTGA | TGG | 59 | 4 | chr9:-123320978 |  |

**Table S3:** **Primer sequences used to amplify and sequence off-target and on-target regions.**

| **sgRNA Name** | **Target** | **Forward primer**  **(5’-> 3’)** | **Reverse primer**  **(5’-> 3’)** |
| --- | --- | --- | --- |
| sgRNA#1 *Apex1* | *Apex1* On-Target | TTGTGCCTCCAAGAGACCAA | AGTGTTGGGGTAGAGATGCC |
| sgRNA#1 *Apex1* | Off-Target 1 | - | - |
| sgRNA#1 *Apex1* | Off-Target 2 | - | - |
| sgRNA#1 *Apex1* | Off-Target 3 | CCCTAGCTGGAAAGGAAACC | TTTTCTGGAAGTTGCCTGGT |
| sgRNA#1 *Apex1* | Off-Target 4 | GACCCCACAGAGCATCCAAA | GAAGTTGGGAAGTGAGCCCT |
| sgRNA#1 *Apex1* | Off-Target 5 | AAAGACAGCCCGGTTCTACA | ATTCCCAGCGTTCCCTGTTA |
| sgRNA#1 *Apex1* | Off-Target 6 | TCCTGCCCATGAATGACTGT | TCAGGGGTGATAGCTGGTTG |
| sgRNA#1 *Apex1* | Off-Target 7 | CACCAGAGGATTTGCTTCGG | GTCTGATCGCTCCCTCACAT |
|  |  |  |  |
| sgRNA#2 *Apex1* | *Apex1* On-Target | CTGCTCGAATGCCTTCTGTG | TGGGGAGTAAAGCCAGCATT |
| sgRNA#2 *Apex1* | Off-Target 1 | TCTCAGCCATGTGTTTGCTC | CCATTGGGACATAAGGATGG |
| sgRNA#2 *Apex1* | Off-Target 2 | GCAGAGTGTCTGGAGGAACC | GTGCCTTGCACTGAGAATCA |
| sgRNA#2 *Apex1* | Off-Target 3 | GGCAAACGTTCAGGAGTGAT | TTGGGTTAGGTGAGGGTCAG |
| sgRNA#2 *Apex1* | Off-Target 4 | CTCACTGCCTCACCCACATT | AGATGGCAGGTGAAAAGGCT |
| sgRNA#2 *Apex1* | Off-Target 5 | ACCAGGAGAAAGTTGGCTCA | GGGACTTCACTGGGTGTCTG |
| sgRNA#2 *Apex1* | Off-Target 6 | TAAGCACTCACCCCATGTCA | TGCCAGGAGGAACCTGTATC |
| sgRNA#2 *Apex1* | Off-Target 7 | CACTCTTGGGAGACCAGCTC | TCCACAAGCATATGCACACA |

**Table S4: Antibodies used for flow cytometry.**

| **Experiment** | **Antibody** | **Clone** | **Conjugate** | **Company and cat#** | **Dilution used** |
| --- | --- | --- | --- | --- | --- |
| Primary bone marrow, Annexin V, CellTrace^TM^, IFN experiment, Combinaision treatment | Anti-mouse Lineage cocktail | anti-mouse CD3e (145-2C11)  anti-mouse Ly-6G/Ly-6C (RB6-8C5)  anti-mouse CD11b (M1/70)  anti-mouse CD45R/B220 (clone RA3-6B2)  anti-mouse TER-119 (Ter-119) | Biotin | eBioscience 88-7774-75 | 1/400 |
| Primary bone marrow, Annexin V, CellTrace^TM^, IFN experiment, Combinaision treatment | Streptavidin | - | PerCP-eFluor™ 710 | eBioscience  46-4317-82 | 1/400 |
| Primary bone marrow, CellTrace^TM^,  Cell sorting | Anti-mouse Ly6A/E (Sca-1) | D7 | PE-Cyanine7 | eBioscience  25-5981-82 | 1/400 |
| Primary bone marrow, CellTrace^TM^,  Cell sorting | Anti-mouse c-Kit | 2B8 | APC | eBioscience  17-1171-83 | 1/400 |
| Primary bone marrow, Annexin V, CellTrace^TM^, IFN experiment, Combinaision treatment | Anti-mouse CD150 | mShad150 | PE | eBioscience  12-1502-82 | 1/400 |
| Annexin V, CellTrace^TM^, IFN experiment | Anti-mouse CD48 | HM48-1 | eFluor™  450 | eBioscience  48-0481-82 | 1/400 |
| CellTrace^TM^, Combinaision treatment | Anti-mouse CD48 | HM48-1 | FITC | BD Pharmingen  557484 | 1/400 |
| Annexin V, CellTrace^TM^, IFN experiment, Combinaision treatment,  Cell sorting | Fixable Viability Dye | - | eFluor™ 780 | eBioscience  65-0865-14 | 1/2000 |
| Primary bone marrow | Anti-mouse CD48 | HM48-1 | APC-eFluor™ 780 | eBioscience  47-0481-82 | 1/400 |
| CellTrace^TM^ Violet | - | - | Violet  (405/450) | Invitrogen  C34571 | 5µM/10^6^cells |
| CellTrace^TM^  CFSE | - | - | CFSE  (495/519) | Invitrogen  C34570 | 5µM/10^6^cells |
| Annexin V,  IFN experiment, Combinaision treatment | Anti-mouse  c-Kit | 2B8 | PE-Cyanine7 | BioLegend  105814 | 1/400 |
| Annexin V,  IFN experiment | Anti-mouse Ly6A/E (Sca-1) | D7 | Brilliant Violet 605 | BioLegend  108134 | 1/400 |
| Annexin V | Annexin V | - | APC | BioLegend  640941 | 1/200 |
| IFN experiment, Combinaision treatment | Anti-mouse CD201 (EPCR) | RCR-16 | APC | BioLegend  141505 | 1/400 |
| IFN experiment | Anti-mouse CD34 | RAM34 | FITC | eBioscience  11-0341-85 | 1/400 |
| Peripheral blood, primary bone marrow | anti-mouse CD45.2 | 104 | eFluor™ 450 | eBioscience  48-0454-82 | 1/250 for blood, 1/400 for bone marrow |
| Peripheral blood, primary bone marrow | anti-mouse CD45.1 | A20 | Brilliant Violet 605 | BioLegend  110738 | 1/250 for blood, 1/400 for bone marrow |
| Peripheral blood, primary bone marrow | anti-mouse CD45.1 | A20 | APC-Cyanine7 | BioLegend  110716 | 1/250 for blood, 1/400 for bone marrow |
| Peripheral blood, primary bone marrow | anti-mouse CD3e | 145-2C11 | APC | eBioscience  17-0031-82 | 1/167 for blood,  1/333 for bone marrow |
| Peripheral blood, primary bone marrow | anti-mouse/human CD11b | M1/70 | PE | BioLegend  101207 or BD Pharmingen  557397 | 1/500 for blood, 1/1000 for bone marrow |
| Peripheral blood, primary bone marrow | anti-mouse Ly-6G/Ly-6C | RB6-8C5 | PE | BioLegend  108407 or BD Pharmingen  553128 | 1/500 for blood, 1/1000 for bone marrow |
| Peripheral blood, primary bone marrow | anti-mouse/human CD45R | RA3-6B2 | PE-Cyanine7 | eBioscience  25-0452-82 | 1/500 for blood, 1/1000 for bone marrow |
| Cell sorting,  MACS purity analysis | Anti-mouse Lineage cocktail | anti-mouse CD3e (145-2C11)  anti-mouse Ly-6G/Ly-6C (RB6-8C5)  anti-mouse CD11b (M1/70)  anti-mouse CD45R/B220 (clone RA3-6B2)  anti-mouse TER-119 (Ter-119) | FITC | BioLegend  133301 | 1/400 |
| Cell sorting | Anti-mouse Lineage cocktail | anti-mouse CD3e (145-2C11)  anti-mouse Ly-6G/Ly-6C (RB6-8C5)  anti-mouse CD11b (M1/70)  anti-mouse CD45R/B220 (clone RA3-6B2)  anti-mouse TER-119 (Ter-119) | APC | BD Pharmingen  51-9003632 | 1/100-1/200 |
| Cell sorting | Anti-mouse c-Kit | ACK2 | PE | eBioscience  12-1172-83 | 1/400 |
| Combinaision treatment, Cell sorting | Anti-mouse Ly6A/E (Sca-1) | D7 | Super Bright 436 | eBioscience 62-5981-80 | 1/400 |

Multiple different lots of the flow cytometry antibodies have been used over the course of this study.

**Table S5:** **TotalSeq™-A anti-mouse antibodies used for 10X Genomic sequencing**

|  | **Description** | **Clone** | **Barcode A-** | **BioLegend cat#** | **Sequence** |
| --- | --- | --- | --- | --- | --- |
| 1 | CD4 | RM4-5 | 0001 | 100569 | AACAAGACCCTTGAG |
| 2 | CD8a | 53-6.7 | 0002 | 100773 | TACCCGTAATAGCGT |
| 3 | CD366 | RMT3-23 | 0003 | 119729 | ATTGGCACTCAGATG |
| 4 | CD279 | RMP1-30 | 0004 | 109123 | GAAAGTCAAAGCACT |
| 5 | CD117 | 2B8 | 0012 | 105843 | TGCATGTCATCGGTG |
| 6 | Ly-6C | HK1.4 | 0013 | 128047 | AAGTCGTGAGGCATG |
| 7 | CD11b | M1/70 | 0014 | 101265 | TGAAGGCTCATTTGT |
| 8 | Ly-6G | 1A8 | 0015 | 127655 | ACATTGACGCAACTA |
| 9 | CD49f | GoH3 | 0070 | 313633 | TTCCGAGGATGATCT |
| 10 | CD54 | YN1/1.7.4 | 0074 | 116127 | ATAACCGACACAGTG |
| 11 | CD90.2 | 30-H12 | 0075 | 105345 | CCGATCAGCCGTTTA |
| 12 | CD15 | MC-480 | 0076 | 125615 | GCTAGTTTGTGCTGC |
| 13 | CD73 | TY/11.8 | 0077 | 127227 | ACACTTAACGTCTGG |
| 14 | CD49d | R1-2 | 0078 | 103623 | CGCTTGGACGCTTAA |
| 15 | CD200 | OX-90 | 0079 | 123811 | TCAATTCCGGTAGTC |
| 16 | IgG1, κ isotype | MOPC-21 | 0090 | 400199 | GCCGGACGACATTAA |
| 17 | IgG2a, κ isotype | MOPC-173 | 0091 | 400285 | CTCCTACCTAAACTG |
| 18 | IgG2b, κ isotype | MPC-11 | 0092 | 400373 | ATATGTATCACGCGA |
| 19 | CD19 | 6D5 | 0093 | 115559 | ATCAGCCATGTCAGT |
| 20 | IgG2b κ isotype | RTK4530 | 0095 | 400673 | GATTCTTGACGACCT |
| 21 | CD25 | PC61 | 0097 | 102055 | ACCATGAGACACAGT |
| 22 | CD135 | A2F10 | 0098 | 135316 | GTAGCAAGATTCAAG |
| 23 | CD45R/B220 | RA3-6B2 | 0103 | 103263 | CCTACACCTCATAAT |
| 24 | CD102 | 3C4 (MIC2/4) | 0104 | 105613 | GATATTCAGTGCGAC |
| 25 | CD115 | AFS98 | 0105 | 135533 | TTCCGTTGTTGTGAG |
| 26 | CD11c | N418 | 0106 | 117355 | GTTATGGACGCTTGC |
| 27 | CD21,CD35 | 7E9 | 0107 | 123427 | GGATAATTTCGATCC |
| 28 | CD23 | B3B4 | 0108 | 101635 | TCTCTTGGAAGATGA |
| 29 | CD16/32 | 93 | 0109 | 101343 | TTCGATGCTGGAGCA |
| 30 | CD43 | S11 | 0110 | 143211 | TTGGAGGGTTGTGCT |
| 31 | CD5 | 53-7.3 | 0111 | 100637 | CAGCTCAGTGTGTTG |
| 32 | CD62L | MEL-14 | 0112 | 104451 | TGGGCCTAAGTCATC |
| 33 | CD93 | AA4.1 | 0113 | 136513 | GGTATTTCCTGTGGT |
| 34 | F4/80 | BM8 | 0114 | 123153 | TTAACTTCAGCCCGT |
| 35 | FcεRIα | MAR-1 | 0115 | 134333 | AGTCACCTCGAAGCT |
| 36 | I-A/I-E | M5/114.15.2 | 0117 | 107653 | GGTCACCAGTATGAT |
| 37 | NK-1.1 | PK136 | 0118 | 108755 | GTAACATTACTCGTC |
| 38 | Siglec H | 551 | 0119 | 129615 | CCGCACCTACATTAG |
| 39 | TCR β chain | H57-597 | 0120 | 109247 | TCCTATGGGACTCAG |
| 40 | TCR γ/δ | GL3 | 0121 | 118137 | AACCCAAATAGCTGA |
| 41 | TER-119 | TER-119 | 0122 | 116247 | GCGCGTTTGTGCTAT |
| 42 | Ly-6A/E | D7 | 0130 | 108147 | TTCCTTTCCTACGCA |
| 43 | CD146 | P1H12 | 0134 | 361017 | CCTTGGATAACATCA |
| 44 | CD278 | C398.4A | 0171 | 313555 | CGCGCACCCATTAAA |
| 45 | CD206 | C068C2 | 0173 | discontinued | TCAACTCGGTGTTGC |
| 46 | CD3 | 17A2 | 0182 | 100251 | GTATGTCCGCTCGAT |
| 47 | CD335 | 29A1.4 | 0184 | 137633 | CCCTTTCACCTCGAA |
| 48 | CD274 | MIH6 | 0190 | 153604 | TCGATTCCACCAACT |
| 49 | CD27 | LG.3A10 | 0191 | 124235 | CAAGGTATGTCACTG |
| 50 | CD20 | SA275A11 | 0192 | 150423 | TCCACTCCCTGTATA |
| 51 | CD357 | DTA-1 | 0193 | 126319 | GGCACTCTGTAACAT |
| 52 | CD137 | 17B5 | 0194 | 106111 | TCCCTGTATAGATGA |
| 53 | CD134 | OX-86 | 0195 | 119426 | CTCACCTACCTATGG |
| 54 | CD69 | H1.2F3 | 0197 | 104546 | TTGTATTCCGCCATT |
| 55 | CD127 | A7R34 | 0198 | 135045 | GTGTGAGGCACTCTT |
| 56 | CD86 | GL-1 | 0200 | 105047 | CTGGATTTGTGTATC |
| 57 | CD103 | 2E7 | 0201 | 121437 | TTCATTAGCCCGCTG |
| 58 | CD64 | X54-5/7.1 | 0202 | 139325 | AGCAATTAACGGGAG |
| 59 | CD150 | TC15-12F12.2 | 0203 | 115945 | CAACGCCTAGAAACC |
| 60 | TCR Vγ1.1 | 2.11 | 0209 | 141113 | TCGTTTAACCAGCCT |
| 61 | TCR Vγ3 | 536 | 0210 | 137507 | TCGTGGTCCCTTTCT |
| 62 | TCR Vγ2 | UC3-10A6 | 0211 | 137709 | AAGCTGCACCGTAAT |
| 63 | CD24 | M1/69 | 0212 | 101841 | TATATCTTTGCCGCA |
| 64 | Integrin β7 | FIB504 | 0214 | 321227 | TCCTTGGATGTACCG |
| 65 | ERK1 | W15133A | 0222 | discontinued | CGCCACTTCATTCAT |
| 66 | RORg | 2F7-2 | 0223 | discontinued | TTCCCTACGCCGAAT |
| 67 | CD196 | 29-2L17 | 0225 | 129825 | CTCTCTGCATTCCTC |
| 68 | CD106 | 429 (MVCAM.A) | 0226 | 105725 | CGTTCCTACCTACCT |
| 69 | CD62P | RMP-1 | 0229 | discontinued | TGTGTGCCGTAGACT |
| 70 | CD8b | YTS156.7.7 | 0230 | 126623 | TTCCCTCTATGGAGC |
| 71 | MAdCAM-1 | MECA-367 | 0232 | 120713 | TTGGGCGATTAAGAA |
| 72 | TCR Vβ8.1,8.2 | KJ16-133.18 | 0235 | 118415 | ACTATCCGTTGTGCT |
| 73 | IgG1, κ isotype | RTK2071 | 0236 | 400459 | ATCAGATGCCCTCAT |
| 74 | IgG1, λ isotype | G0114F7 | 0237 | 401919 | GGGAGCGATTCAACT |
| 75 | IgG2a, κ isotype | RTK2758 | 0238 | 400571 | AAGTCAGGTTCGTTT |
| 76 | IgG2a, isotype |  | 0239 | Discontinued | CATTAACAGCGCCAA |
| 77 | Rat IgG2c, κ isotype | RTK4174 | 0240 | 400739 | TCCAGGCTAGTCATT |
| 78 | IgG isotype | HTK888 | 0241 | 400973 | CCTGTCATTAAGACT |
| 79 | IRF4 | IRF4.3E4 | 0249 | discontinued | GGATTTGTATCTCCC |
| 80 | KLRG1 | 2F1/KLRG1 | 0250 | 138431 | GTAGTAGGCTAGACC |
| 81 | TCR Vβ5.1, 5.2 | MR9-4 | 0354 | 139517 | CTCAACAGTATTCTG |
| 82 | CD195 | HM-CCR5 | 0376 | 107019 | ACCAGTTGTCATTAC |
| 83 | CD223 | C9B7W | 0378 | 125229 | ATTCCGTCCCTAAGG |
| 84 | CD62E | RME-1/CD62E | 0379 | discontinued | CTCCCTTTGTAACAT |
| 85 | CD90/CD90.1 | OX-7 | 0380 | 202547 | AGTATGGGATGCAAT |
| 86 | Panendothelial Cell Antigen | MECA-32 | 0381 | 120507 | CGTCCTAGTCATTGG |
| 87 | CD152 | UC10-4B9 | 0388 | 106325 | AGTGTTTGTCCTGGT |
| 88 | P2RY12 | S16007D | 0415 | 848009 | TTGCTTATTTCCGCA |
| 89 | CD300LG | ZAQ5 | 0416 | 147105 | CGGTCCGTATCATTT |
| 90 | CD163 | S15049I | 0417 | 155303 | GAGCAAGATTAAGAC |
| 91 | CD49b | HMα2 | 0421 | 103523 | CGCGTTAGTAGAGTC |
| 92 | CD172a (SIRPα) | P84 | 0422 | 144033 | GATTCCCTTGTAGCA |
| 93 | CD14 | Sa14-2 | 0424 | 123333 | AACCAACAGTCACGT |
| 94 | CD192 (CCR2) | SA203G11 | 0426 | 150625 | AGTGCGATCTGCAAC |
| 95 | CD48 | HM48-1 | 0429 | 103447 | AGAACCGCCGTAGTT |
| 96 | ReceptorD4 |  | 0434 | discontinued | TCTCTGCACCGCTTT |
| 97 | GABRB3 |  | 0435 | discontinued | GGTGTGAGCAGTTCT |
| 98 | CD201 | RCR-16 | 0439 | 141509 | TATGATCTGCCCTTG |
| 99 | CD169 | 3D6.112 | 0440 | 142425 | ATTGACGACAGTCAT |
| 100 | CD71 | RI7217 | 0441 | 113824 | ACCGACCAGTAGACA |
| 101 | Notch 1 | HMN1-12 | 0442 | 130617 | TCCGGTCACTCAGTA |
| 102 | CD41 | MWReg30 | 0443 | 133937 | ACTTGGATGGACACT |
| 103 | CD184 | L276F12 | 0444 | 146520 | GTCGTGGTGTTGTTC |
| 104 | CD204 | 1F8C33 | 0448 | 154703 | AGCTAGACACGTTGT |
| 105 | CD326 | G8.8 | 0449 | 118237 | ACCCGCGTTAGTATG |
| 106 | IgM | RMM-1 | 0450 | 406535 | AGCTACGCATTCAAT |
| 107 | CD301a | LOM-8.7 | 0551 | 145611 | TGTATTTACTCACCG |
| 108 | CD304 | 3E12 | 0552 | 145215 | CCAGCTCATTCAACG |
| 109 | CD309 | 89B3A5 | 0554 | 121921 | AGTTGTCCTGTACGA |
| 110 | CD36 | HM36 | 0555 | 102621 | TTTGCCGCTACGACA |
| 111 | CD370 | 7H11 | 0556 | discontinued | AACTCAGTTGTGCCG |
| 112 | CD38 | 90 | 0557 | 102733 | CGTATCCGTCTCCTA |
| 113 | CD55 | RIKO-3 | 0558 | 131809 | ATTGTTGTCAGACCA |
| 114 | CD63 | NVG-2 | 0559 | 143915 | ATCCGACACGTATTA |
| 115 | CD68 | FA-11 | 0560 | 137031 | CTTTCTTTCACGGGA |
| 116 | CD79b | HM79-12 | 0561 | 132811 | TAACTCAGTGCGAGT |
| 117 | CD83 | Michel-19 | 0562 | 121519 | TCTCAGGCTTCCTAG |
| 118 | CX3CR1 | SA011F11 | 0563 | 149041 | CACTCTCAGTCCTAT |
| 119 | Folate Receptor β | 10/FR2 | 0564 | 153307 | CTCAGATGCCCTTTA |
| 120 | MERTK | 2B10C42 | 0565 | discontinued | AGTAGAGCAACTCGT |
| 121 | CD301b | URA-1 | 0566 | 146817 | CTTGCCTTGCGATTT |
| 122 | Tim-4 | RMT4-54 | 0567 | 130011 | TGCTGGAGGGTATTC |
| 123 | XCR1 | ZET | 0568 | 148227 | TCCATTACCCACGTT |
| 124 | CD29 | HMβ1-1 | 0570 | 102233 | ACGCATTCCTTGTGT |
| 125 | IgD | 11-26c.2a | 0571 | 405745 | TCATATCCGTTGTCC |
| 126 | CD140a | APA5 | 0573 | 135917 | GTCATTGCGGTCCTA |
| 127 | CD11a | M17/4 | 0595 | 101125 | AGAGTCTCCCTTTAG |
| 128 | ESAM | 1G8/ESAM | 0596 | 136209 | TATAGTTTCCGCCGT |
| 129 | CD200R | OX-110 | 0807 | 123913 | ATTCTTTCCCTCTGT |
| 130 | CD193 | J073E5 | 0808 | 144523 | TAGAACCGTATCCGT |
| 131 | CD200R3 | Ba13 | 0809 | 142209 | ATCAACTTGGAGCAG |
| 132 | CD138 | 281-2 | 0810 | 142532 | GCGTTTGTATGTACT |
| 133 | CD317 | 927 | 0811 | 127027 | TGTGGTAGCCCTTGT |
| 134 | CD105 | MJ7/18 | 0812 | 120421 | TATCCCTGCCTTGCA |
| 135 | CD9 | MZ3 | 0813 | 124819 | TAGCAGTCACTCCTA |
| 136 | P2X7R | 1F11 | 0824 | 148711 | TGCTTCATTCATGTG |
| 137 | CD371 | 5D3/CLEC12A | 0825 | 143407 | GCGAGAAATCTGCAT |
| 138 | CD22 | OX-97 | 0827 | 126113 | AGGTCCTCTCTGGAT |
| 139 | CD39 | Duha59 | 0834 | 143813 | GCGTATTTAACCCGT |
| 140 | CD314 | CX5 | 0835 | 130215 | GAGGCTTATCATTTC |
| 141 | DR3 | 4C12 | 0836 | 144413 | GCTTGGGCAATTAAG |
| 142 | IL-33Rα | DIH9 | 0837 | 145317 | GCGATGGAGCATGTT |
| 143 | Ly49H | 3D10 | 0839 | 144715 | CCAGTAGGCTTATTA |
| 144 | Ly49D | 4E5 | 0841 | 138309 | TATATCCCTCAACGC |
| 145 | CD185 | L138D7 | 0846 | 145535 | ACGTAGTCACCTAGT |
| 146 | TIGIT | 1G9 | 0848 | 142115 | GAAAGTCGCCAACAG |
| 147 | CD80 | 16-10A1 | 0849 | 104745 | GACCCGGTGTCATTT |
| 148 | CD49a | HMα1 | 0850 | 142613 | CCATTCATTTGTGGC |
| 149 | CD1d | 1B1 | 0851 | 123529 | CAACTTGGCCGAATC |
| 150 | CD226 | 10E5 | 0852 | 128823 | ACGCAGTATTTCCGA |
| 151 | CD34 | HM34 | 0857 | 128619 | GATTCCTTTACGAGC |
| 152 | TLR4 (CD284)/MD2 Complex | MTS510 | 0875 | 117614 | GCAGTTGTCCGATTC |
| 153 | CD300c/d | TX52 | 0876 | 148005 | GTGATCTAAGATGCG |
| 154 | JAML | 4E10 | 0877 | 128507 | GTTATGGTTCGTGTT |
| 155 | CD272 | 6A6 | 0881 | 139113 | TGACCCTATTGAGAA |
| 156 | PIR-A/B | 6C1 | 0882 | 144105 | TGTAGAGTCAGACCT |
| 157 | CD26 | H194-112 | 0883 | 137811 | ATGGCCTGTCATAAT |
| 158 | DLL1 | HMD1-3 | 0884 | 128315 | AGACCTCCTTACGAT |
| 159 | CD270 | HMHV-1B18 | 0885 | 136307 | GATCCGTGTTGCCTA |
| 160 | CD137L | TKS-1 | 0890 | 107109 | CAGTTCAGTACGCAG |
| 161 | ENPP1 | YE1/19.1 | 0891 | 149209 | CATTAACCGCCCTTA |
| 162 | CD2 | RM2-5 | 0892 | 100117 | TTGCCGTGTGTTTAA |
| 163 | Mac-2 | M3/38 | 0895 | 125421 | GATGCAATTAGCCGG |
| 164 | CD31 | 390 | 0904 | 102437 | GCTGTAGTATCATGT |
| 165 | CD107a | 1D4B | 0905 | 121635 | AAATCTGTGCCGTAC |
| 166 | CD124 | I015F8 | 0916 | 144809 | GAACCGTAGTATAAC |
| 167 | CD95 (Fas) | SA367H8 | 0917 | 152614 | CACATCGTTTGTGTA |
| 168 | Ly6D |  | 5106 | discontinued | ATGTCCTACCTCAAA |

All the ADT were diluted according to the information published in Scheyltjens *et al*. 2022 ^[9]^.

**Table S6: Chi-square test on the cluster proportions of the different annotated RNA-seq clusters, for each of the treated HSPC culture.**

|  |  | **adj. P-value** *(cut-off < 0.05)* | | | **Log2 fold change between observed versus expected cluster proportions** | | |
| --- | --- | --- | --- | --- | --- | --- | --- |
|  |  | **Inh. III** | **E3330** | **APX2009** | **Inh. III** | **E3330** | **APX2009** |
| Cluster **0** | HSCs | 2,31x10^6^ | 2,99x10^16^ | 3,35x10^10^ | -0,3960825 | 0,327541 | 0,363174 |
| Cluster **1** | “stressed” HSPCs | 0,000138 | 0,287886 | 0,101744 | -0,27246 | -0,05567 | -0,12253 |
| Cluster **2** | myeloid prog. | 0,225226 | 4,2x10^122^ | 2,07x10^96^ | 0,16068668 | 0,862136 | 1,113362 |
| Cluster **3** | LSKs | 4,18x10^58^ | 6,2x10^119^ | 2,75x10^72^ | -1,1918198 | -1,86894 | -1,7209 |
| Cluster **4** | GMPs | 0,009625 | 0,734111 | 0,02979 | 0,16533788 | 0,018569 | 0,161229 |
| Cluster **5** | MEPs | 8,19x10^53^ | 5,06x10^11^ | 0,000268 | 0,81274444 | 0,406844 | 0,359949 |
| Cluster **6** | LMPs | 1,2x10^11^ | 3,77x10^16^ | 5,63 x10^8^ | -0,7632607 | -0,75726 | -0,62814 |
| Cluster **7** | Monocytes | 1,99x10^11^ | 2,25 x10^5^ | 0,00307 | 0,48363978 | -0,4622 | -0,43485 |
| Cluster **8** | DCPs/LPs | 1,79x10^5^ | 1,44x10^10^ | 0,101744 | 0,36532313 | -0,91269 | -0,22995 |
| Cluster **9** | MBPs | 3,65x10^25^ | 0,000585 | 0,328577 | 0,89985851 | -0,88601 | -0,25428 |
| Cluster **10** | “stressed” GMPs | 0,177703 | 0,001286 | 0,779239 | 0,22012837 | 0,355299 | 0,053846 |

**Table S7: Chi-square test on the cluster proportions of the different ADT-gated HSPC populations.**

|  | **adj. P-value** *(cut-off < 0.05)* | | | **Log2 fold change between observed versus expected cluster proportions** | | |
| --- | --- | --- | --- | --- | --- | --- |
|  | **Inh. III** | **E3330** | **APX2009** | **Inh. III** | **E3330** | **APX2009** |
| **LSK-SLAM EPCR+** | 0,943437 | 0,037442 | 4,99x10^5^ | 0,023514 | 0,419429 | 0,821185 |
| **LSK-SLAM EPCR-** | 0,062132 | 0,396892 | 0,844374 | -0,35891 | -0,1065 | 0,030945 |
| **Lin- Sca1+ ckit+ (LSK)** | 1,25x10^21^ | 3,71x10^61^ | 9,39x10^39^ | -0,42901 | -0,70782 | -0,71938 |
| **Lin- Sca1+ ckit-** | 0,08724 | 0,045611 | 0,006801 | -0,26494 | -0,24723 | -0,51281 |
| **Lin- Sca1- ckit+** | 3,99x10^10^ | 1,06x10^70^ | 8,67x10^51^ | 0,169239 | 0,304016 | 0,365899 |
| **Lin- Sca1- ckit-** | 3,67x10^7^ | 5,83x10^7^ | 0,058874 | 0,496338 | 0,395919 | 0,248662 |
| **CD11b+ Ly6C+ (Lin+)** | 0,041055 | 4,29x10^16^ | 4,84x10^10^ | 0,133592 | -0,61139 | -0,61518 |
| **LSK-SLAM (EPCR+/-)** | 0,08844 | 0,72728 | 0,021005 | -0,2798 | 0,03631 | 0,290382 |
| **MPP2** | 2,18x10^7^ | 0,00044 | 0,095969 | -0,79097 | -0,35433 | -0,20324 |
| **MPP3/MPP4** | 1,03x10^16^ | 9,76x10^51^ | 1,85x10^33^ | -0,54891 | -1,00492 | -1,05246 |
| **MPP5** | 0,683022 | 2,76x10^6^ | 4,56x10^5^ | -0,03565 | -0,42273 | -0,50687 |
| **Lin- (without LSK)** | 3,67x10^15^ | 3,2x10^86^ | 3,47x10^50^ | 0,178456 | 0,287309 | 0,321725 |

**Supplementary Methods**

**
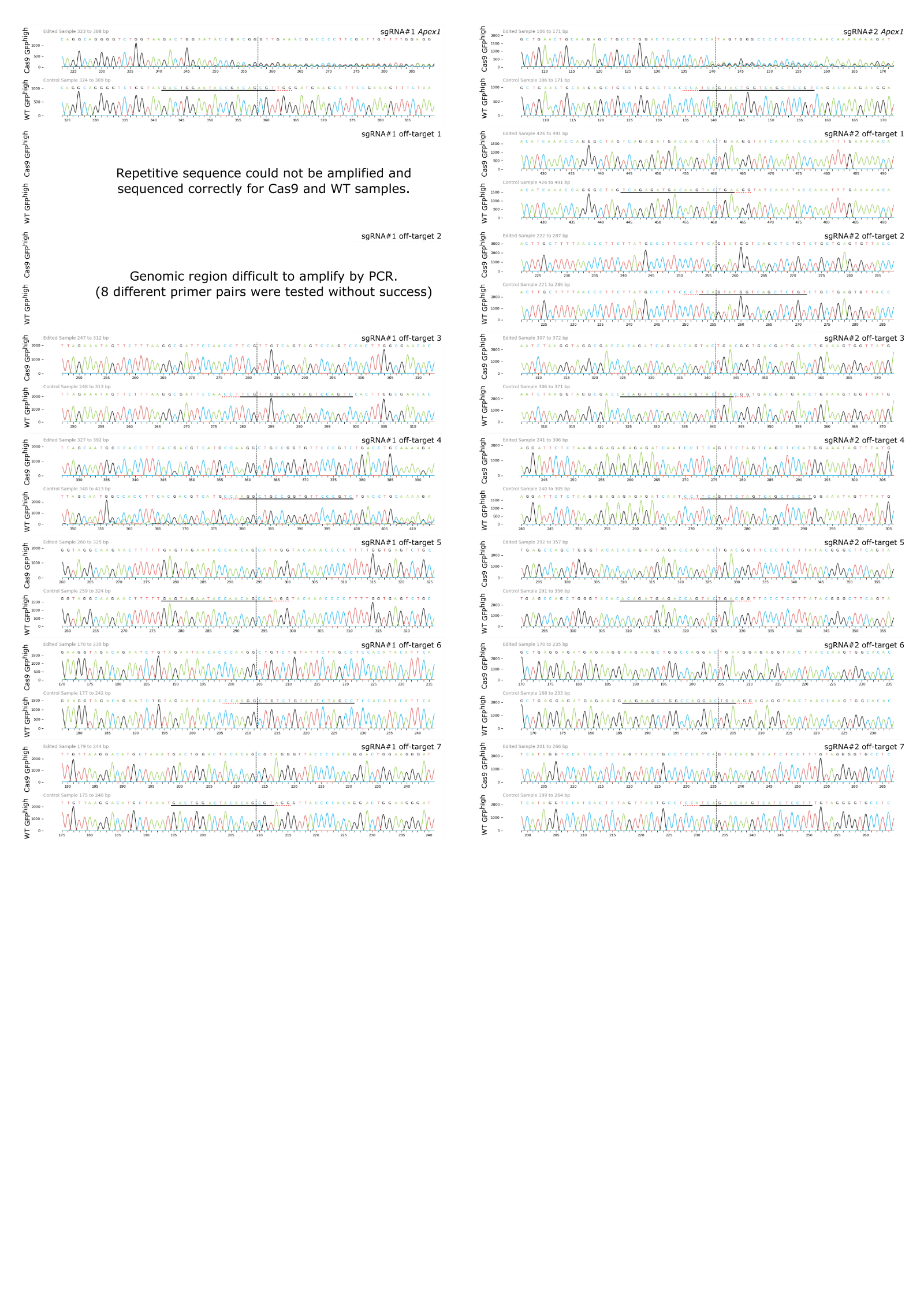
Figure S1 (related to main Figure 1): On-target and off-target analysis.** On-target site and the top 7 identified off-target sites (Table S2) of both *Apex1* sgRNAs at 2 weeks post-transduction, were sequenced using Sanger sequencing, and aligned between the Cas9 GFP^high^ cells and non-edited control cells (WT GFP^high^ cells). The Cas9 cutting site is indicated by a vertical black dotted line, PAM sequence is underlined by a horizontal red dotted line and the sgRNA sequence is underlined vertically in black. N=1.


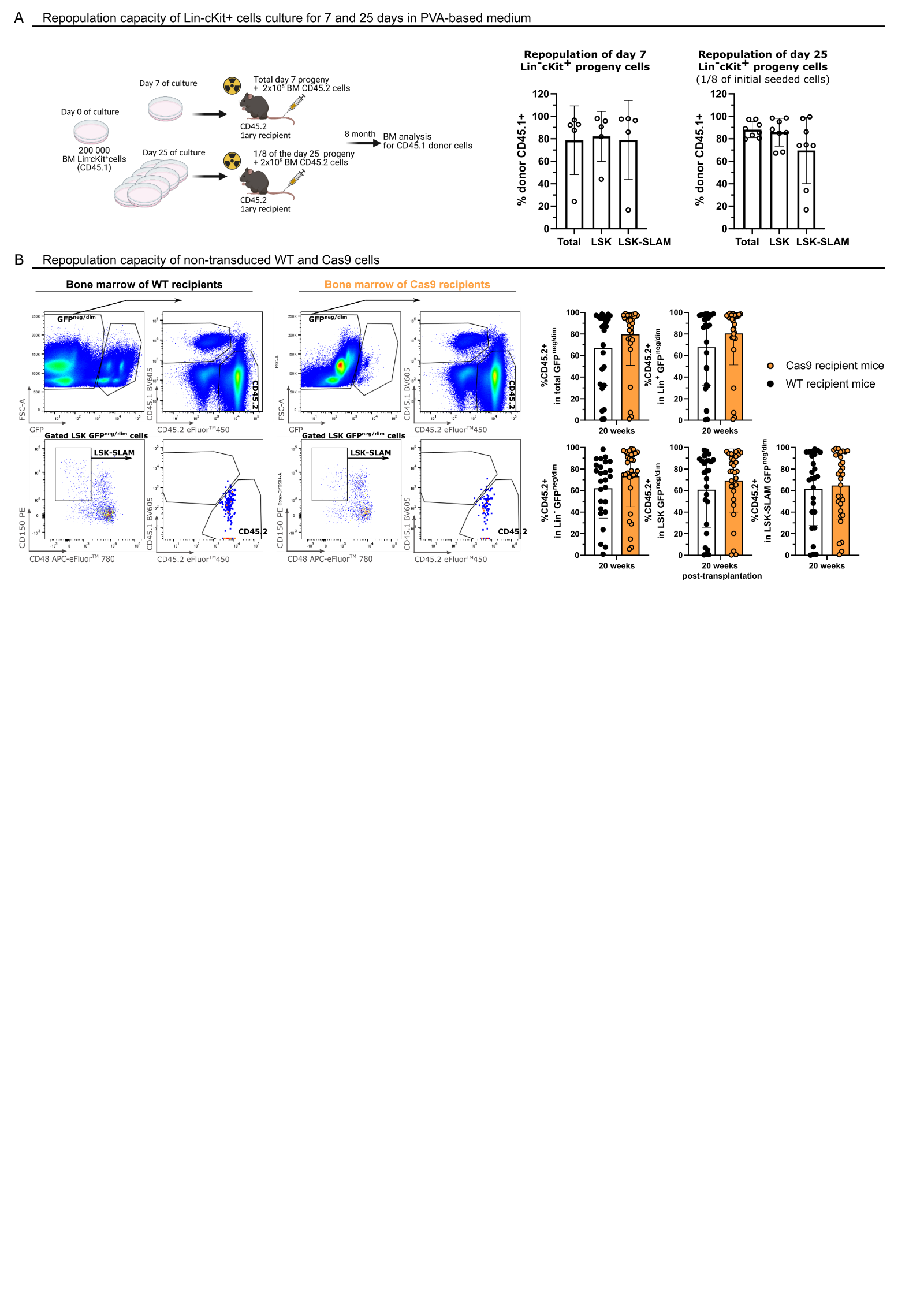


**Figure S2 (related to main Figure 1): Repopulation capacity of non-transduced Cas9 cells and long-term PVA-based expanded HSPCs.** (A) Experimental-set of the PVA-based HSPC expansion repopulation experiment (left) and bone marrow repopulation percentage of the donor cells (% CD45.1) in the total, LSK and LSK-SLAM compartment at 8 months after transplantation (right). N=1 independent experiments, with 2 initial donor sets and a total of 5-8 transplanted mice perday of culture. (B) Representative flow cytometry plots and quantification of the bone marrow chimerism (CD45.2 donor %) of the non-transduced Cas9 and WT cells (GFP^neg/dim^) in the total, lineage-committed (Lin^+^) and HSPC (Lin^-^, LSK and LSK-SLAM) populations ± 20 weeks after transplantation. N=4 independent experiments, with a total of 26-31 mice per group. Mann-Whitney test for all comparisons. Data bars represent the mean±SD. p < 0.05 (*), p < 0.01 (**), p < 0.001 (***), p < 0.0001 (****).


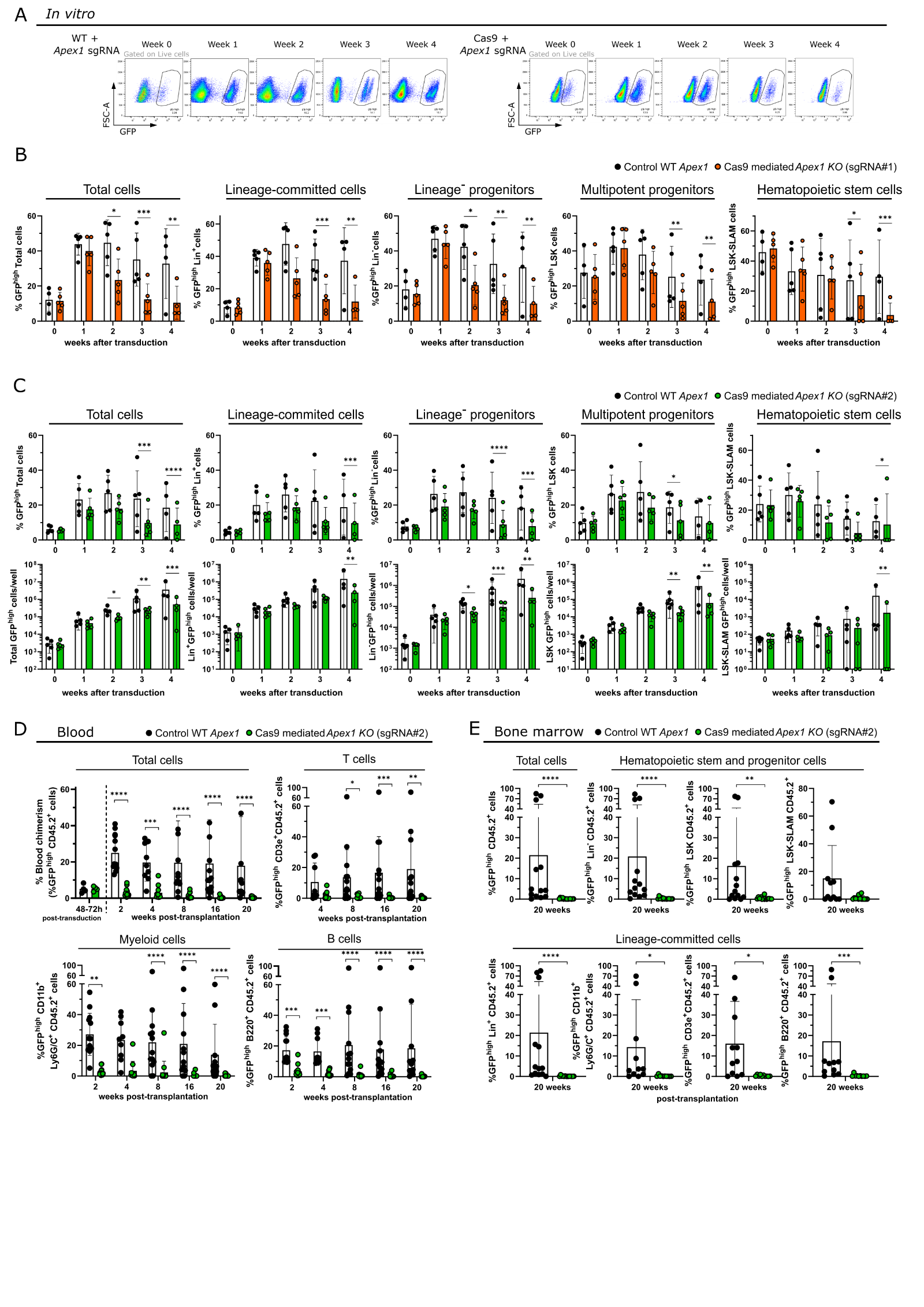


**Figure S3 (related to main Figure 1): APEX1 maintains HSPC function during *ex vivo* expansion and *in vivo* hematopoietic repopulation.** (A) Representative flow cytometry plots showing the percentage of GFP^high^ cells during the 4-week expansion. (B) Percentage of CRISPR-Cas9 mediated APEX1 KO cells (Cas9 GFP^high^ cells, orange dots) or WT cells (WT GFP^high^ cells, black dots), using sgRNA#1, in the total viable cells, Lin^+^, Lin^-^, LSK and LSK-SLAM cells, during a 4-week expansion culture. Percentage of GFP^high^ cells at week 0 displays the initial transduction efficiency in the different HSPC population. (C) Percentage and absolute number of CRISPR-Cas9 mediated APEX1 KO cells (Cas9 GFP^high^ cells, green dots) or WT cells (WT GFP^high^ cells, black dots), using sgRNA#2, in the total viable cells, Lin^+^, Lin^-^, LSK and LSK-SLAM cells, during a 4-week expansion culture. Log scaled axis was used for the expansion graphs. For (B) and (C), N=5 independent experiments with 5 independent donors for each group, except for the 4-week time point where N=4. Percentage of GFP^high^ cells at week 0 displays the initial transduction efficiency in the different HSPC population. (D) Blood percentage of APEX1 KO or WT cells (GFP^high^ cells) present in total and leucocyte lineages (B, T and myeloid) of CD45.2^+^ donor cells at 2, 4, 8, 16, and 20 weeks post-transplantation. Initial transduction efficiency of transplanted cells is shown as percentage of GFP^high^ cells 48-72hours after transduction. N=4 independent experiments for sgRNA#2, with a total of 9-15 mice per group and timepoint. (E) Bone marrow percentage of APEX1 KO or WT cells (GFP^high^ cells) in the total, lineage-committed (Lin^+^, B, T and myeloid), and HSPC (Lin^-^, LSK and LSK-SLAM) CD45.2^+^ donor cells ± 20 weeks after transplantation. N=4 independent experiments for sgRNA#2, with a total of 13-14 mice per group. Sidak post-hoc tests (following a two-way ANOVA/Mixed model repeated measures analysis) were used to compare the 2 groups (Cas9 and WT) at the different timepoints, in (B), (C) and (D). Mann-Whitney test for all comparisons in (E). Data bars represent the mean±SD. p < 0.05 (*), p < 0.01 (**), p < 0.001 (***), p < 0.0001 (****).

**
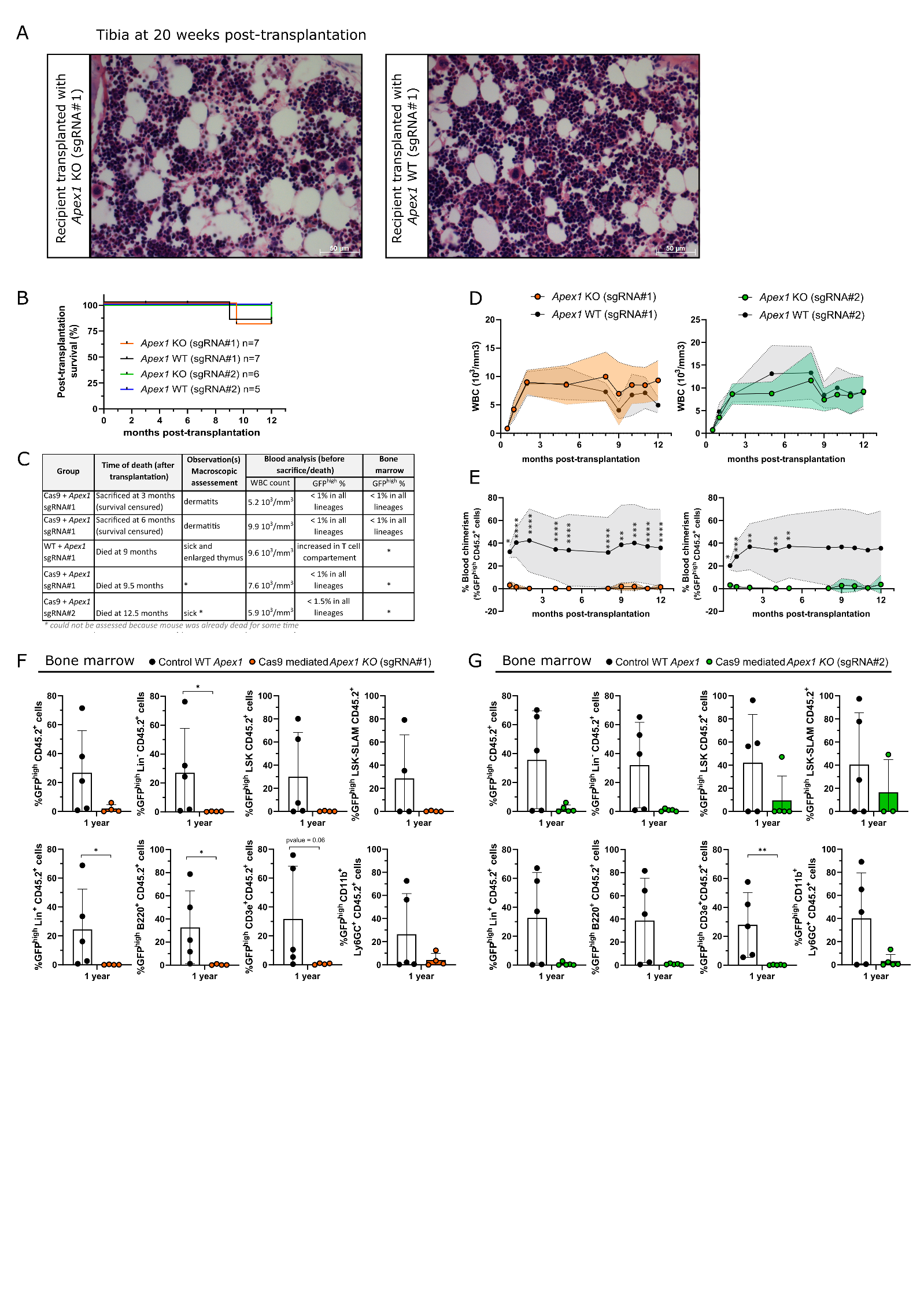
Figure S4 (related to main Figure 1): HSPC APEX1 deficiency does not cause hematological malignancy development 1 year after transplantation.** (A) Representative hematoxylin-eosin stained section of the tibia of transplanted mice with APEX1 KO or WT (sgRNA#1) 20 weeks after transplantation. Left image is from 1 of the 2 APEX1 KO transplanted mice with increased GFP^high^ LSK and LSK-SLAM compartment after 20 weeks, right image shows a matched control mouse transplanted with APEX1 WT HSPCs. (B) Post-transplantation survival curves of mice transplanted with APEX1 KO and WT HSPCs, for both sgRNAs. n indicated on the graph represents the total number of transplanted mice per group, pooled from 2 independent experiments. No significant difference in survival was detected between the APEX1 KO and WT group for each sgRNA using the Mantel Cox test. (C) Table containing detailed information about early sacrificed mice due to dermatitis and dead mice, during the 1-year follow-up. (D) White blood cell (WBC) counts of all transplanted recipients with APEX1 KO and WT HSPCs, followed for 1-year after transplantation. (E) Peripheral blood analysis at different timepoints during the 1-year post-transplantation follow-up. Percentage of all the APEX1 KO or WT cells (GFP^high^ cells) present in total leucocyte of CD45.2^+^ donor cells, at different timepoints during the 1 year post-transplantation follow-up. (F) (G) Bone marrow percentage of APEX1 KO or WT cells (GFP^high^ cells) in the total, lineage-committed (Lin^+^, B, T and myeloid), and HSPC (Lin^-^, LSK and LSK-SLAM) CD45.2^+^ donor cells. Sidak post-hoc tests (following a two-way ANOVA/Mixed model repeated measure analysis) were used to compare the 2 groups (Cas9 and WT) for each sgRNA at the different timepoints, in (D) and (E). Mann-Whitney test was used for all comparisons in (F) and (G). Data bars represent the mean±SD. p < 0.05 (*), p < 0.01 (**), p < 0.001 (***), p < 0.0001 (****).

**
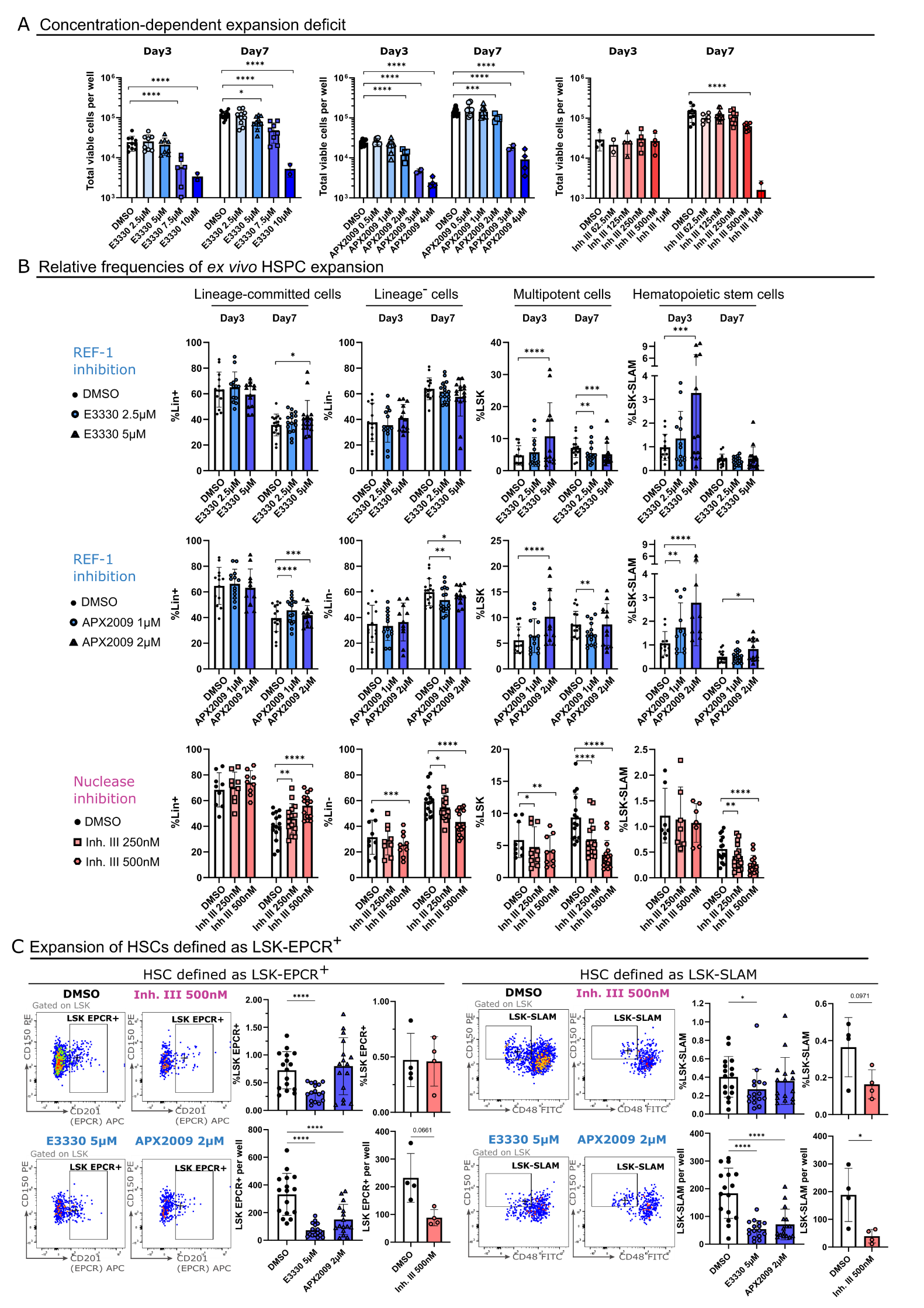
Figure S5** **(related to main Figure 2):** **Concentration-dependent expansion deficiency after APEX1 inhibitor exposure.** (A) Flow cytometry quantification of total viable cell progeny of Lin^-^cKit^+^ cells exposed to various concentration of E3330, APX2009 and Inhibitor III. The lowest drug concentrations showing a significant effect on expansion after 7-days and the concentration just below, were chosen for further characterization of the APEX1 inhibition effects. N=1-5 independent experiments. Log scaled axis was used for the expansion graphs. (B) Percentages of the Lin^-^cKit^+^ progeny exposed continuously for 3 or 7 days to low concentrations of E3330 or APX2009 REF-1 inhibitors, or Inh. III nuclease inhibitor. N=5-8 independent experiments, with a total of 9-16 donors per group. Dunnett’s post hoc tests (following a two-way ANOVA/Mixed model sample-matched analysis) were used to compare each treated group to their corresponding DMSO control condition in (A) and (B). (C) Representative flow cytometry plots, relative frequencies, and expansion (total number of cells/well) of HSCs defined as LSK EPCR^+^ (left) or defined as LSK-SLAM (right), after 7 days of Lin^-^cKit^+^ culture exposed to REF-1 or nuclease inhibitors. N=8 independent experiments for REF-1 inhibitor treatments, and N=2 for Inh. III treatment, with 2 biological replicates per experiment. Dunnett’s post hoc tests (following a one-way ANOVA sample-matched analysis) was used to compare the REF-1 inhibitors compared to their DMSO control. Paired-t test was performed to compare the Inh. III and DMSO groups.

Data bars represent the mean±SD. p < 0.05 (*), p < 0.01 (**), p < 0.001(***), p < 0.0001 (****).


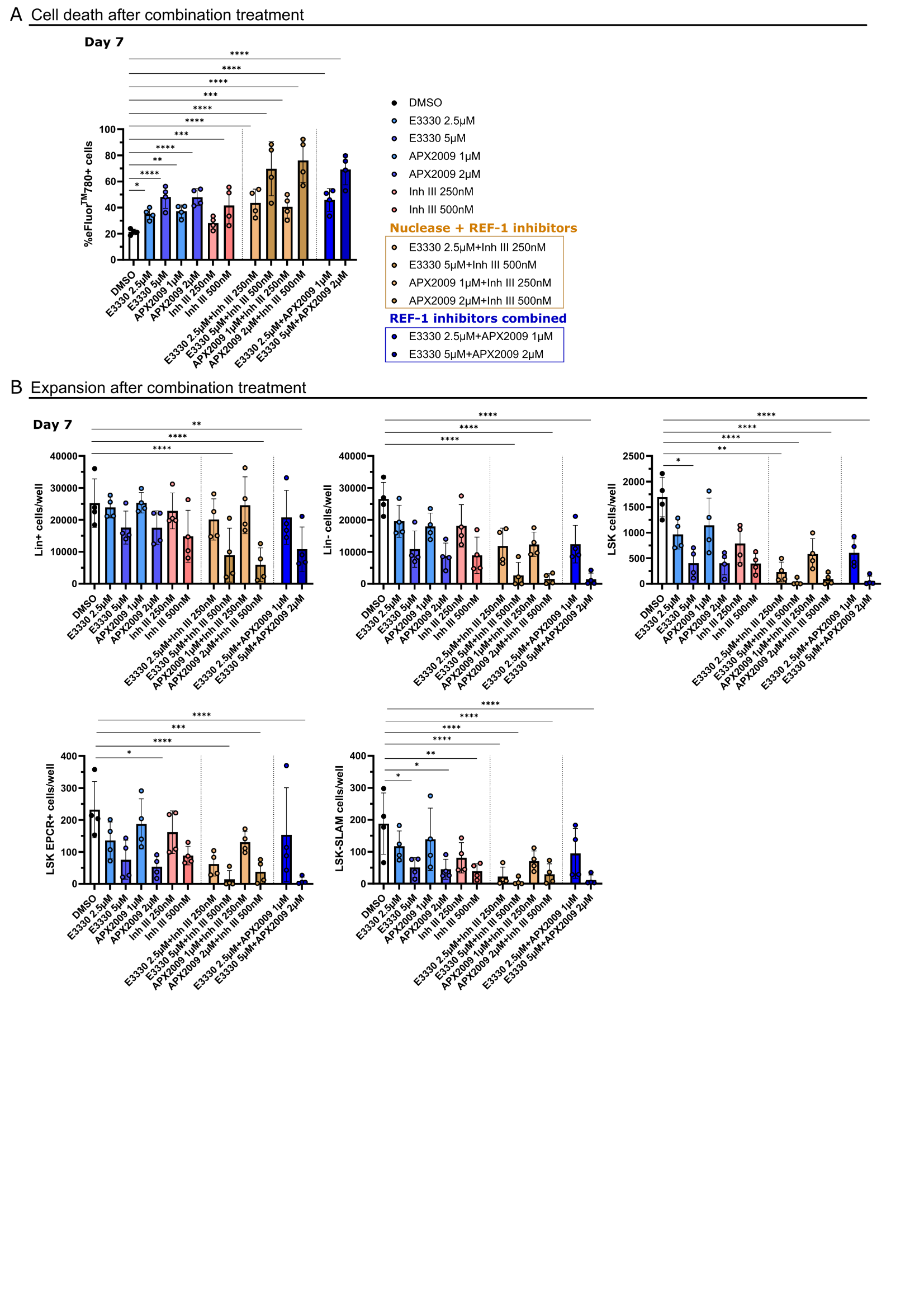


**Figure S6 (related to main Figure 2): Effect of combined treatment with APEX1 REF-1 and nuclease inhibitors on HSPC expansion.** (A) Percentage of dead cells in cultures treated with REF-1 inhibitors (APX2009 or E3330) or the nuclease inhibitor (Inh III) alone, or in combination for 7 days, using the flow cytometry eFluor^TM^780 cell dead permeable dye. (B) Expansion (number of cells/well) of the Lin^-^ cKit^+^ progeny exposed continuously for 7 days to E3330, APX2009, Inh. III alone or in combination. N=2 independent experiments, with 2 biological replicates per experiment. Dunnett’s post hoc tests (following a one-way ANOVA sample-matched analysis) were used to compare the APEX1 inhibitors to their DMSO control. Data bars represent the mean±SD. p < 0.05 (*), p < 0.01 (**), p < 0.001(***), p < 0.0001 (****).


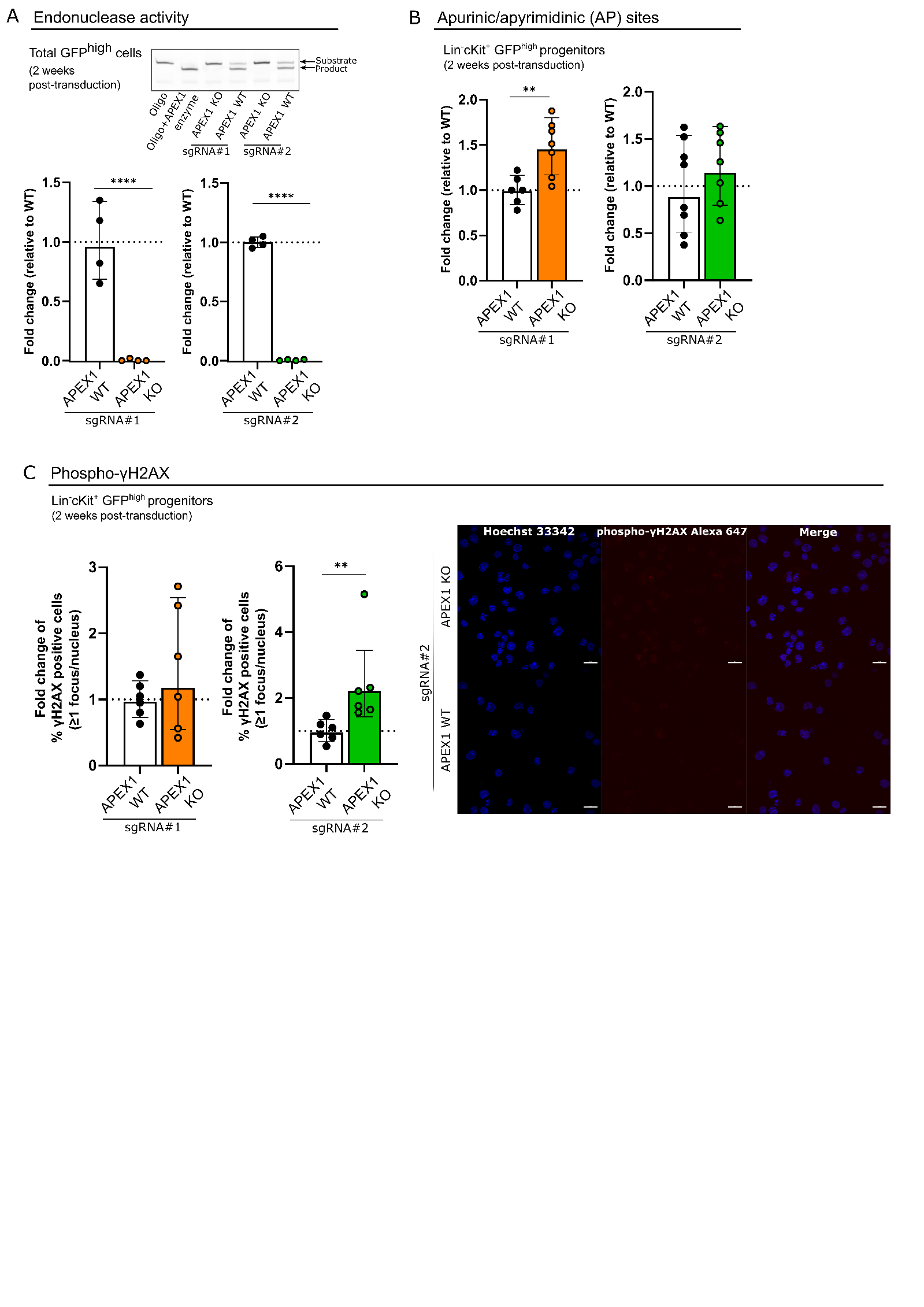


**Figure S7 (related to Figure 3): APEX1 KO induced some DNA damage in HSPCs**. (A) Apurinic/apyrimidinic (AP) incision assay to measure the endonuclease activity in total progeny of APEX1 KO and APEX1 WT 2-weeks expanded Lin^-^cKit^+^ cells. N= 2 indep. experiments with total of 4 biological replicates per group. (B) AP site quantification on Lin^-^cKit^+^ sorted KO and WT cells at 2 weeks after culture/transduction. N= 4 indep. experiments with a total of 6-8 biological replicates per group. (C) Confocal phospho-γH2AX representative images (scale bar represents 20µm) and quantification of Lin^-^cKit^+^ sorted KO and WT cells at 2 weeks after culture. N= 3 indep. experiments with a total of 6 biological replicate per group. Unpaired T test was used for all comparisons. Data bars represent the geometric mean±geometric SD. p < 0.05 (*), p < 0.01 (**), p < 0.001(***), p < 0.0001 (****).


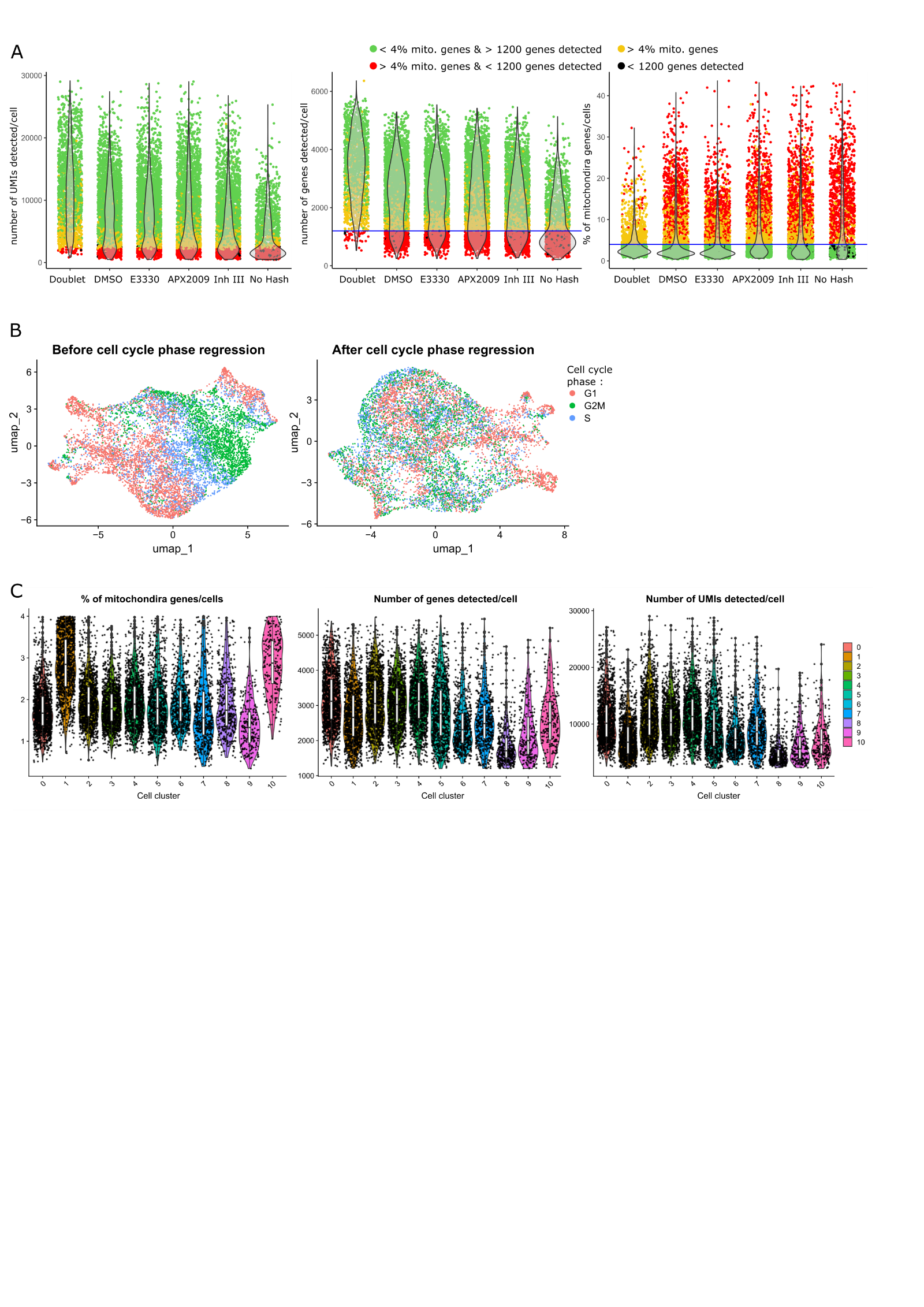


**Figure S8 (related to main Figure 4): Quality control single-cell sequencing data.** (A) Number of unique molecular identifier (UMI), number of genes detected and mitochondrial gene percentage per cell after multivariate scatter QC filtering and before extra filtering steps. Only cells (in green) with less than 4% mitochondrial gene transcripts and more than 1200 genes detected per cell were retained for the CITE-seq analysis. (B) RNA-UMAP of all samples with cell cycle phase classification after SCT normalization without cell cycle phase regression (left) and with cell cycle phase regression (right). (C) Number of UMI, number of genes detected and mitochondrial gene percentage per cell after all filtering steps, for each of the 10 identified CITE-seq cell clusters after cell cycle regression (Figure 4B).


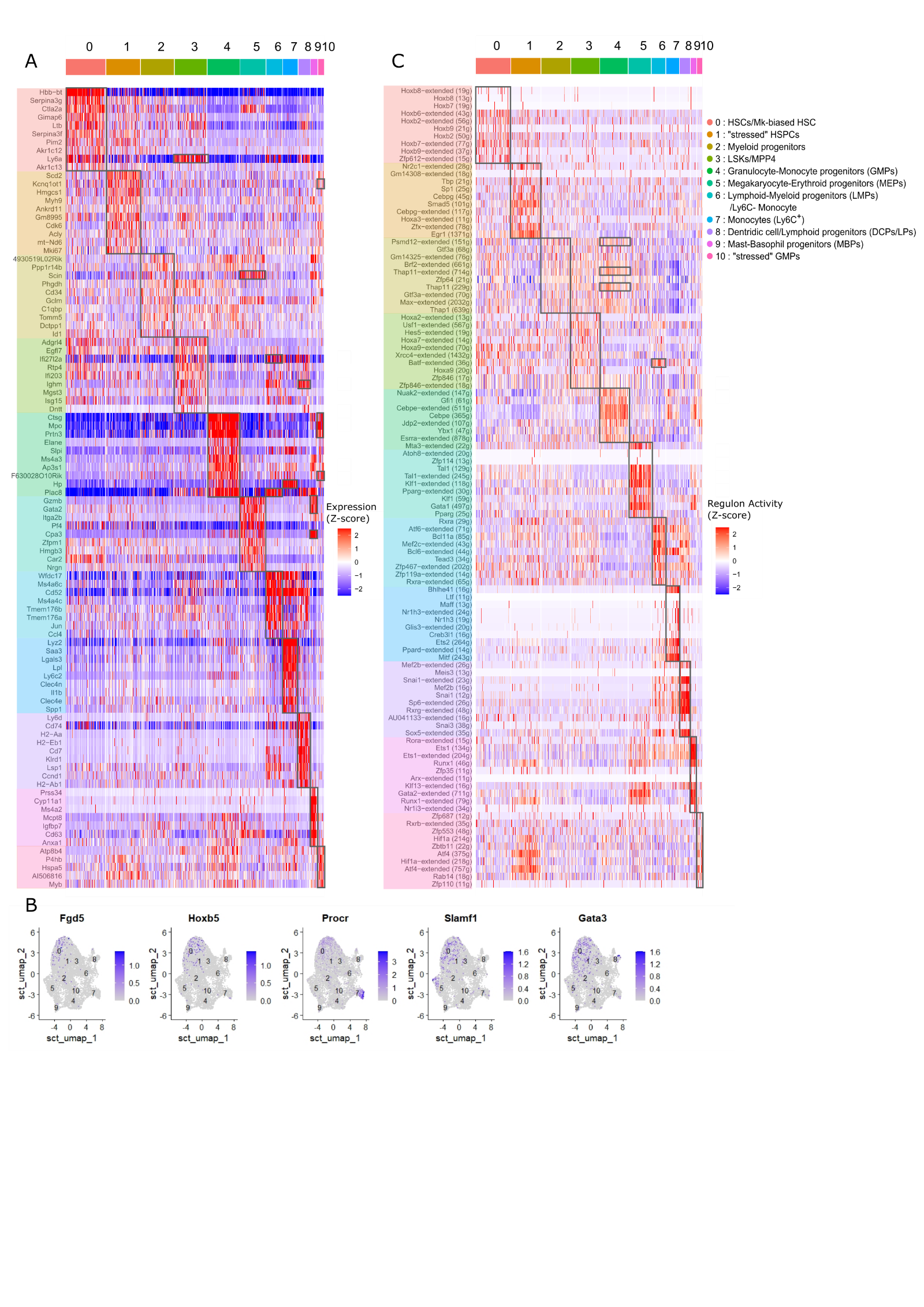


**Figure S9 (related to main Figure 4): Transcriptomic data used for CITE-seq cluster annotation.** (A) Heatmap showing the top 10 differentially expressed genes (DEGs) (top 10 based on fold change) per cluster compared to all other clusters. Cluster marker gene names are highlighted by the cluster colors. The scaled expression of the top 10 DEGs of each cluster is also highlighted by gray squares. (B) Expression of some additional HSC gene markers, highlighted on the RNA-UMAP. (C) Heatmap displaying the top 10 regulons (highest mean regulon activity, Z-score) for each cluster identified by SCENIC. Cluster regulon names are highlighted by the respective cluster colors. The scaled regulon activity of the top 10 regulons of each cluster is also highlighted by gray squares.


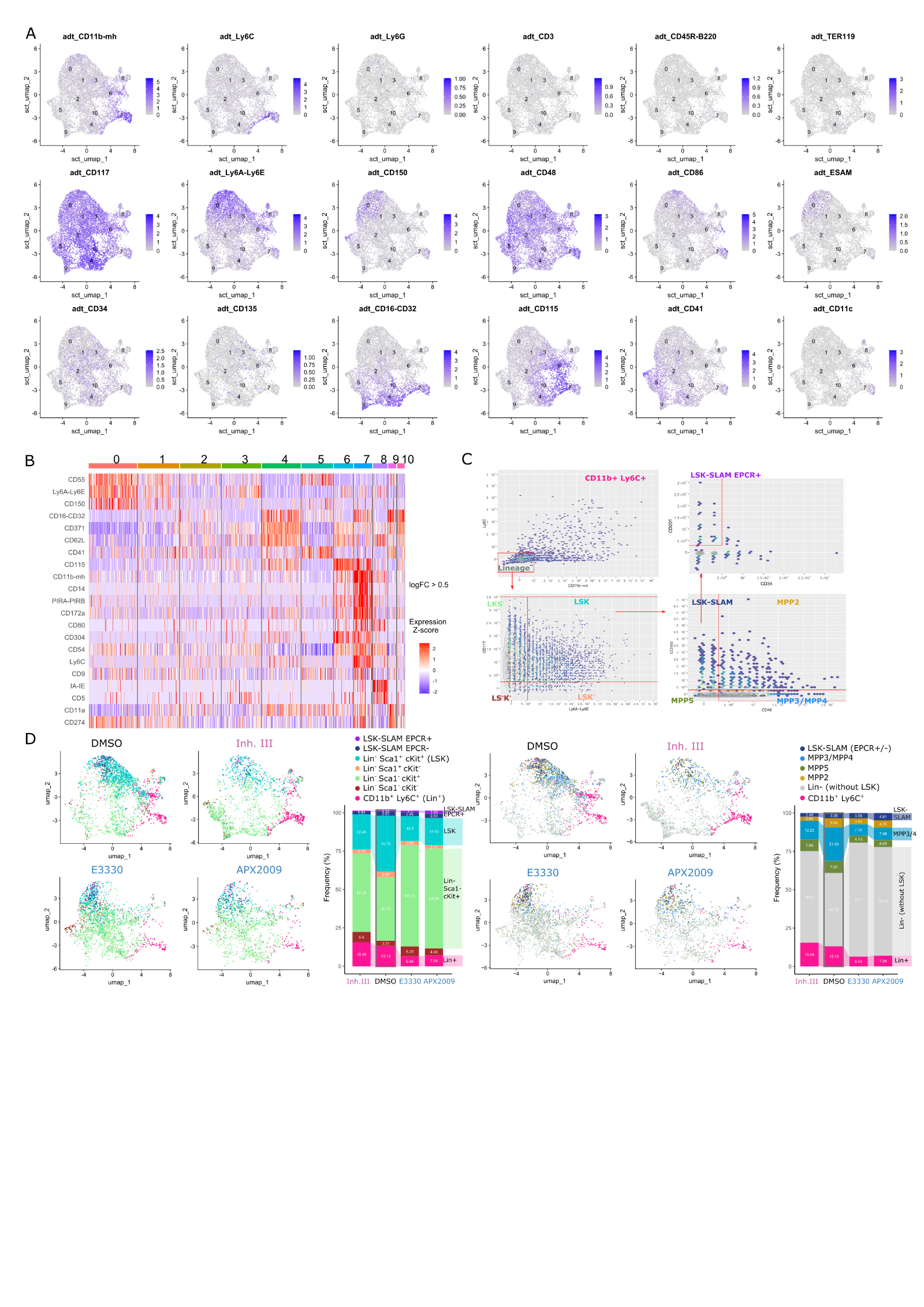


**Figure S10 (related to main Figure 4):** **Antibody-derived tag (ADT) data used for CITE-seq cluster annotation.**

(A) Expression of selected HSPC and lineage commitment surface proteins, highlighted on UMAP from RNA based clustering. (B) Heatmap showing differential expressed ADTs among the RNA clusters (cut-off: log fold change >0.5). (C) ADT gating strategy used for defining the HSPC population based on Ly6C, CD11b, CD117, Ly6A-Ly6E, CD48, CD150, CD34 and CD201 DSB (denoised and scaled by background) normalized expression. (D) ADT-gated HSPC populations across different sample conditions, highlighted on UMAP from RNA based clustering. Chi-square test was performed on cluster proportions (see Table S7).

**
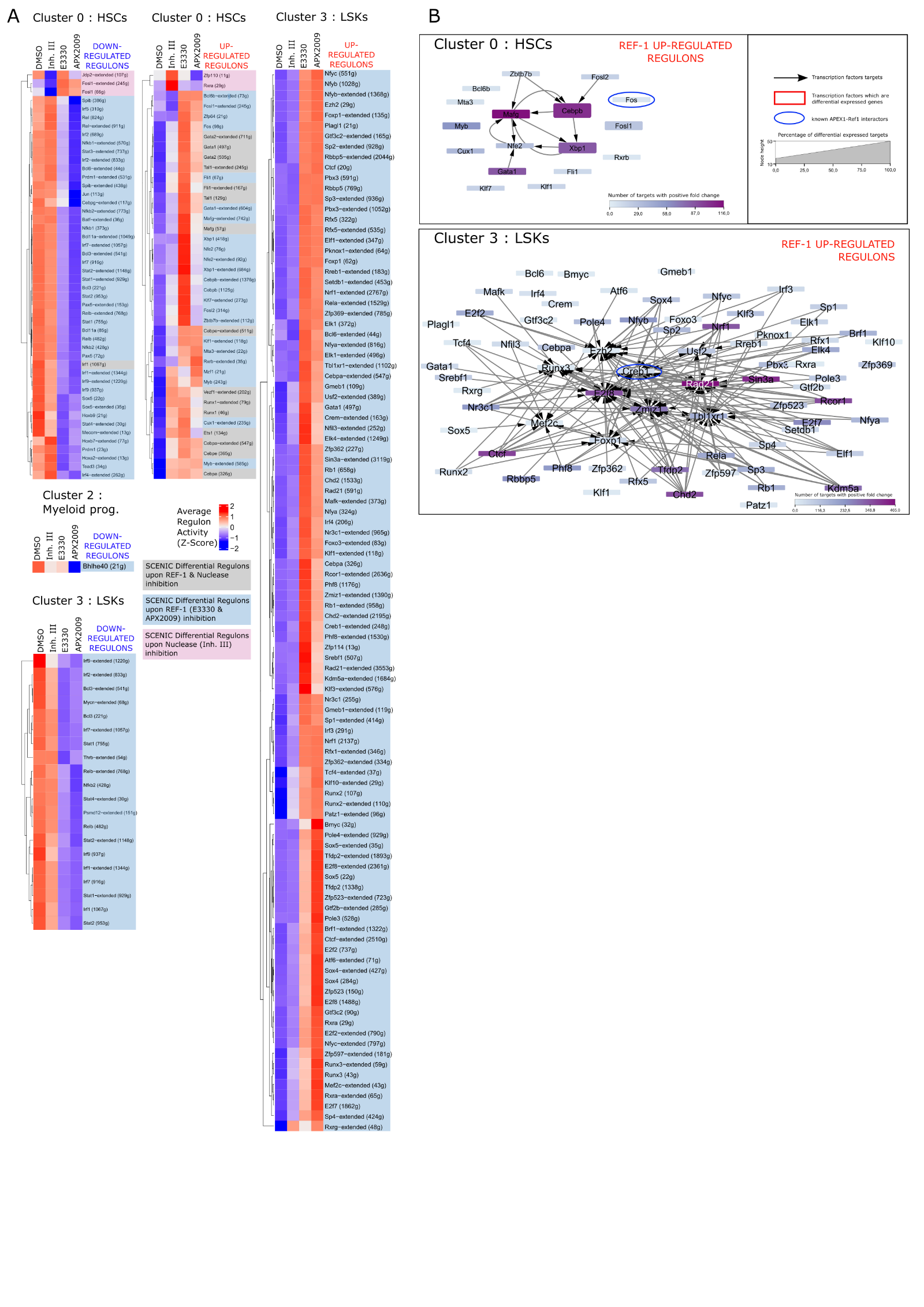
Figure S11 (related to main Figure 5):** **Differentially activated regulon analysis identify downregulated interferon related TFs in HSPC progeny following APEX1 REF1 inhibition.** (A) Heatmaps showing the differentially activated regulons (based on their activity score), between the different APEX1 inhibitor treated HSPCs and DMSO treated HSPCs. Heatmaps display the average scaled activity per cluster (Z-score) for the HSC, LSK and early myeloid progenitor clusters. Similar to the DEGs, common differentially regulated regulons for all 3 inhibitor treatments are highlighted in gray, differential regulons present following both REF-1 inhibitor treatments are highlighted in blue and differential regulons only present following nuclease inhibition with Inh. III are highlighted in pink. Number indicated in between brackets after the regulon, is the number of targets identified in the entire data set by SCENIC analysis. (B) Cytoscape representation of the specific upregulated regulons upon both APEX1-REF-1 inhibition for the HSC cluster 0 and the LSK cluster 3. The nodes are the differentially active transcription factors (TFs) following REF-1 inhibition, and the node size represents the percentage of target genes of each TF that are differential expressed. The node gradient color (from light blue to dark purple) represents the number of target genes that are positively regulated compared to DMSO condition (log2 fold change >0). TFs that are among the DEGs are highlighted by red squares, and the TFs that were previously published to interact directly with REF-1 are highlighted by blue circles. The black arrows indicate when a TF has another TF as target gene.


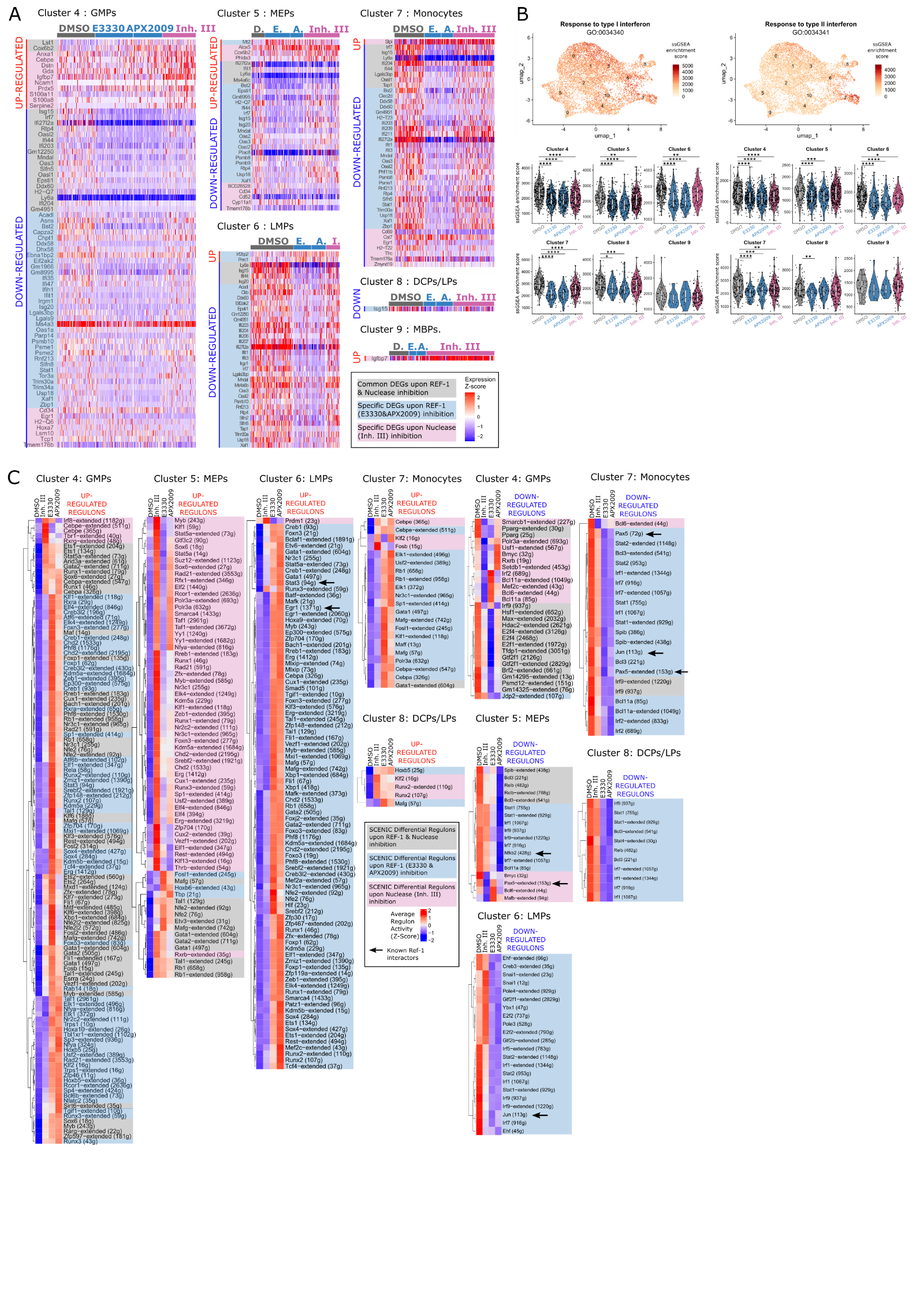
**Figure S12 (related to main Figure 5): Differential gene expression and differential regulon activity analyses of the more committed lineages after 7-days APEX1 REF-1 inhibition and APEX1 nuclease inhibition.** (A) Heatmaps displaying differentially expressed genes (DEGs) in cluster 4 (GMPs), 5 (MEPs), 6 (LMPs), 7 (monocytes), 8 (MBPs) and 9 (DCPs/LPs) following 7-day APEX1 REF-1 (E3330 and APX2009) or APEX1 nuclease (Inhibitor III) inhibition compared to DMSO treated cells. DEGs common to the 3 inhibitor treatments are highlighted in gray, DEGs present following both REF-1 inhibitor treatments are highlighted in blue and DEGs only present following nuclease inhibition with Inh. III are highlighted in pink. (B) RNA-UMAP showing the single-sample gene set enrichment score (ssGSEA) for interferon response using published gene lists (GO:0034340 for type I interferon and GO:0034341 for type II interferon) and corresponding violin plot showing ssGSEA score for cluster 4 (GMPs), 5 (MEPs), 6 (LMPs), 7 (Monocytes), 8 (DCPs/LPs) and 9 (MBPs) across the treatment conditions. Dunn’s post hoc tests (following Kruskal-Wallis rank sum test) were used to compare each treated group to the DMSO. p < 0.05 (*), p < 0.01 (**), p < 0.001(***), p < 0.0001 (****). (C) Heatmaps displaying the differential activated regulons, between the different APEX1 inhibitor treated HSPCs and the DMSO treated HSPCs. Heatmaps display the average scaled activity per cluster (Z-score) for the cluster 4 (GMPs), 5 (MEPs), 6 (LMPs), 7 (Monocytes) and 8 (DCPs/LPs). Similar to the DEGs, common differentially regulated regulons for all 3 inhibitor treatments are highlighted in gray, differential regulons present only following both REF-1 inhibitor treatments are highlighted in blue and differential regulons only present following nuclease inhibition are highlighted in pink. Previous published APEX1 REF-1 interactors are indicated by a black arrow. Number indicated in between brackets after the regulon is the number of targets identified in the entire data set by SCENIC analysis.

**
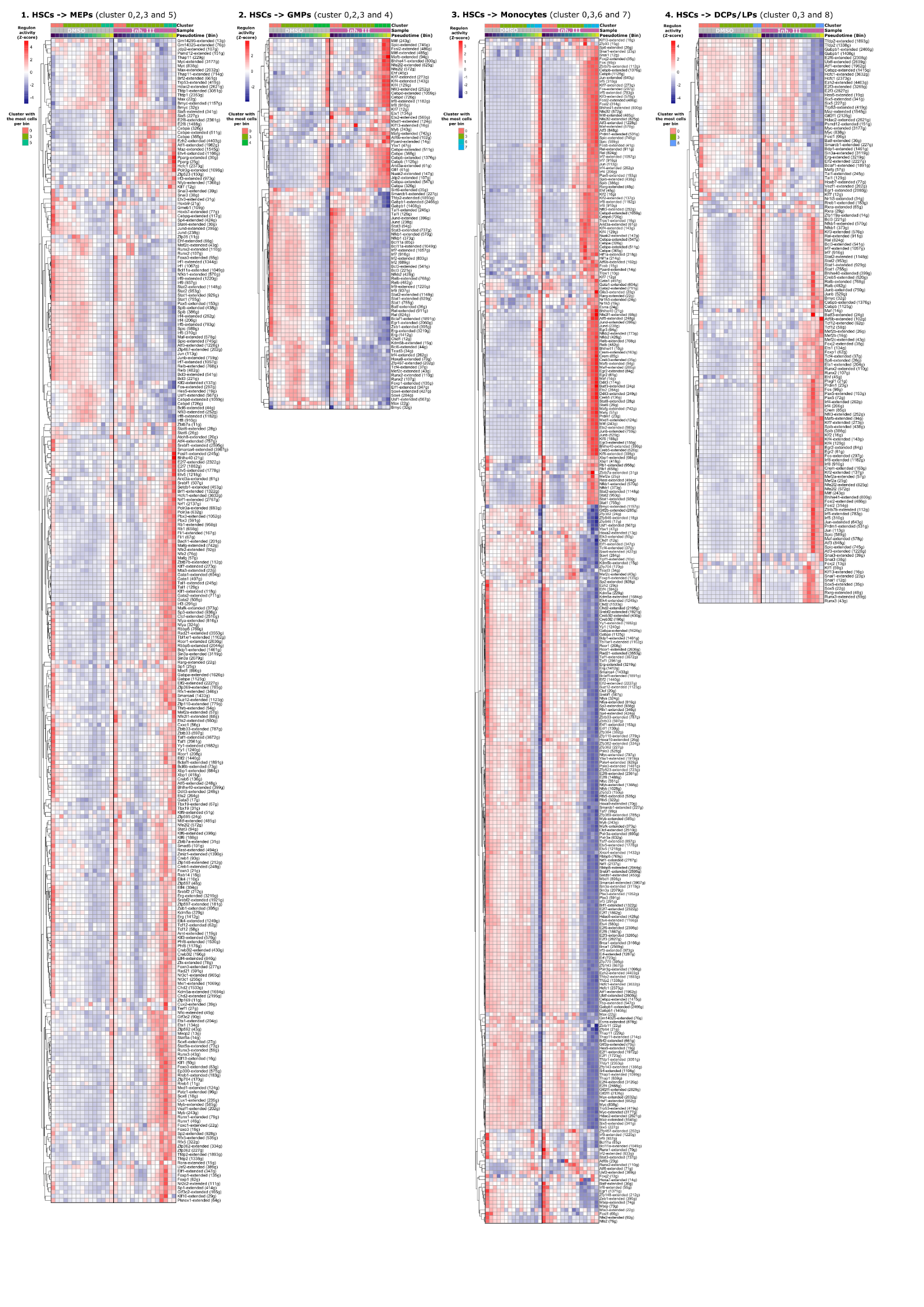
**

**Figure S13 (related to main Figure 6): Differential active regulons along 4 distinct trajectories upon APEX1 nuclease inhibition.** For each of the trajectories, cells were ordered along the differentiation pseudotime (15 bins), and all the SCENIC regulons that showed a significant difference (mixed ANOVA test, with Benjamini–Hochberg correction) in the magnitude (adj. p-value sample) and in the trend (adj. p-value interaction) of AUC score along the pseudotime between the nuclease and DMSO treated conditions are shown on each heatmap. Median regulon activity of each bin was scaled across pseudotime. Regulons were hierarchically clustered, based on their activity score in both conditions. Color bar at the top of the heatmaps indicates which cluster has the most cells for each bin of the pseudotime. Number indicated in between brackets after the regulon, is the number of targets identified in the entire data set by SCENIC analysis. MEP: Megakaryocyte-erythroid progenitor; GMP: granulocyte-monocyte progenitor; DCP/LP: dendritic cell/lymphoid progenitor.

**
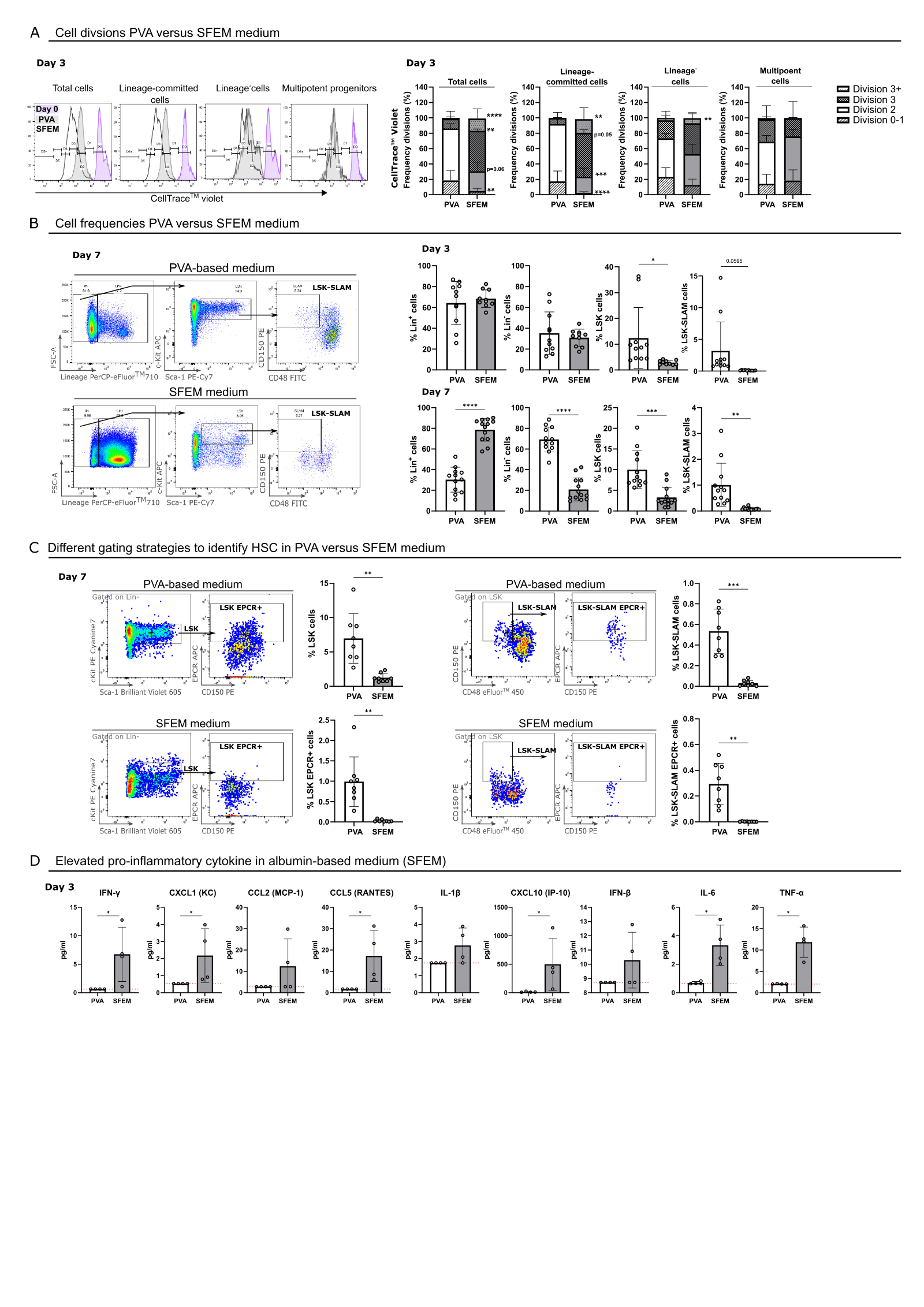
Figure S14 (related to main Figure 7): Faster proliferation, higher lineage-committed (Lin^+^) percentage and higher inflammatory cytokine secretion in SFEM compared to PVA-based medium.** (A) Cell divisions of the Lin^-^ cKit^+^ progeny were analyzed using CellTrace dye (N=3 indep. exp., with 4-5 biological replicates in total) after 3 days of culture in PVA supplemented medium or in SFEM medium. Sidak post hoc tests (following a two way ANOVA/Mixed model sample-matched analysis) were used to compare the cell divisions in PVA medium and in SFEM medium. (B) and (C) Representative flow cytometry plots (day7) and percentage of different Lin^-^cKit^+^ progeny cultured for 3 or 7 days in PVA supplemented medium or in SFEM medium. Paired T test was used to compare the groups, n=6-7 indep. experiments. (D) Cytokine measurement in the supernatant of PVA- and SFEM-based HSPC cultures after 3 days. N=2 independent culture experiments, with 2 biological replicates per experiment. Mann-Whitney test was used to compare the groups. Data bars represent the mean±SD. p < 0.05 (*), p < 0.01 (**), p < 0.001(***), p < 0.0001 (****).

**
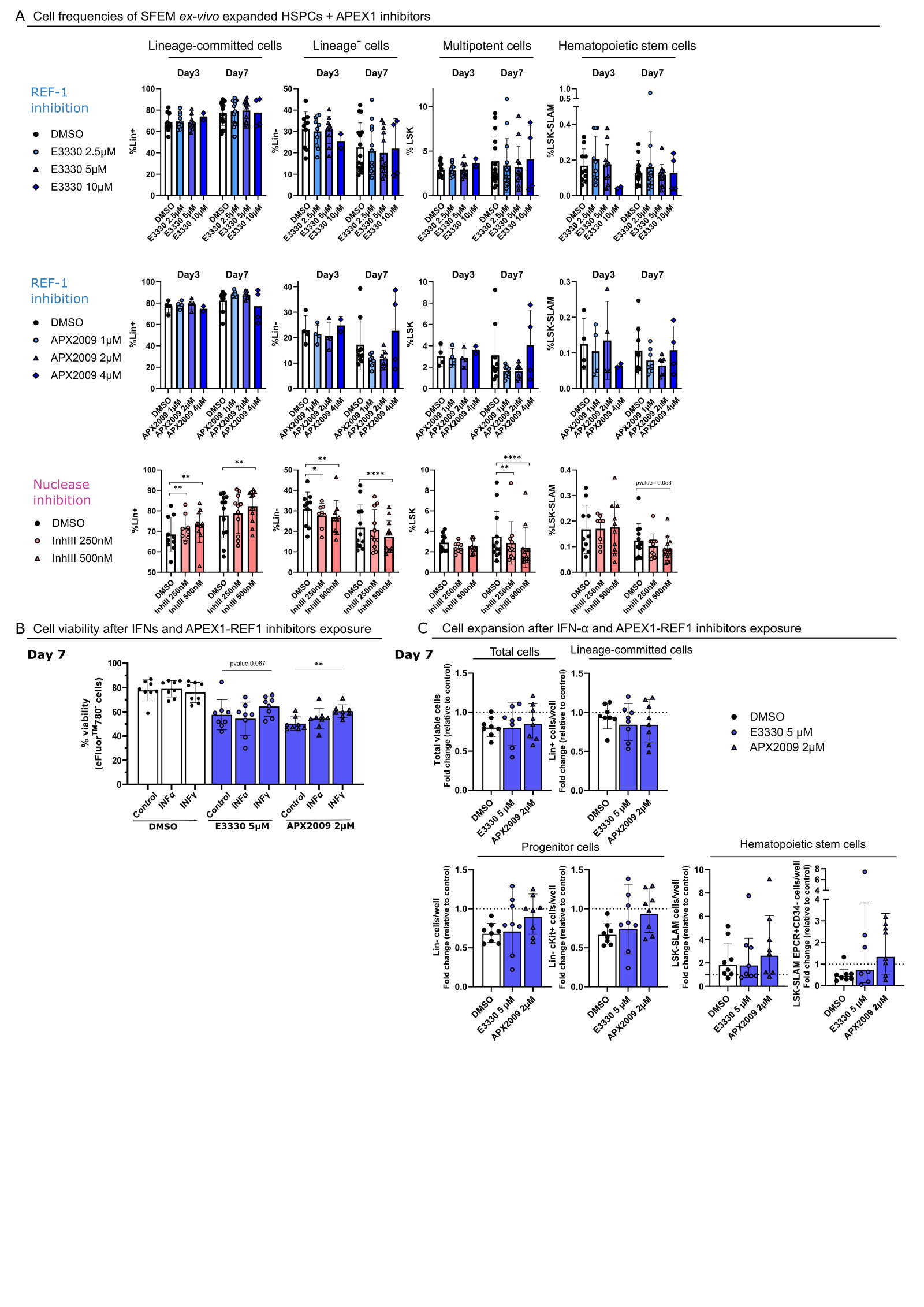
Figure S15 (related to main Figure 7): APEX1 REF-1 inhibition in SFEM medium and in PVA-based medium treated with IFN-α.** (A) Percentage of the different Lin^-^cKit^+^ progeny exposed continuously for 3 or 7 days to the E3330 or APX2009 REF-1 inhibitors, or the Inhibitor III (Inh. III) nuclease inhibitor in albumin-based medium (SFEM). N=2-7 independent experiments, with a total of 4-13 donors per group for day7, and N=1-6 independent experiment for day3, with a total of 2-10 replicates per group. (B) Cell viability (percentage of eFluor^TM^780^neg^ cells) on day 7 in cultures treated with IFN-α or -γ, and with or without REF-1 inhibitors. (C) Expansion of HSPC progeny (total cells, Lin^-^, Lin^-^cKit^+^, LSK-SLAM and LSK-SLAM EPCR^+^CD34^-^) following IFN-α treatment with or without REF-1 inhibitors. Fold change of expansion was calculated for each sample relative to their corresponding non-IFN-α control with or without REF-1 inhibitors. N=4 independent culture experiments in (C) and (B), with a total of 6-8 biological replicates/group. Dunnett’s post hoc tests (following a one-way (in (B) and (C)) or two-way (in (A)) ANOVA/Mixed model matched analysis) were used to compare each treated group to their corresponding DMSO control condition. Data bars represent the mean±SD, except for (C) where data bars represent geometric mean±geometric SD. p < 0.05 (*), p < 0.01 (**), p < 0.001(***), p < 0.0001 (****).

**
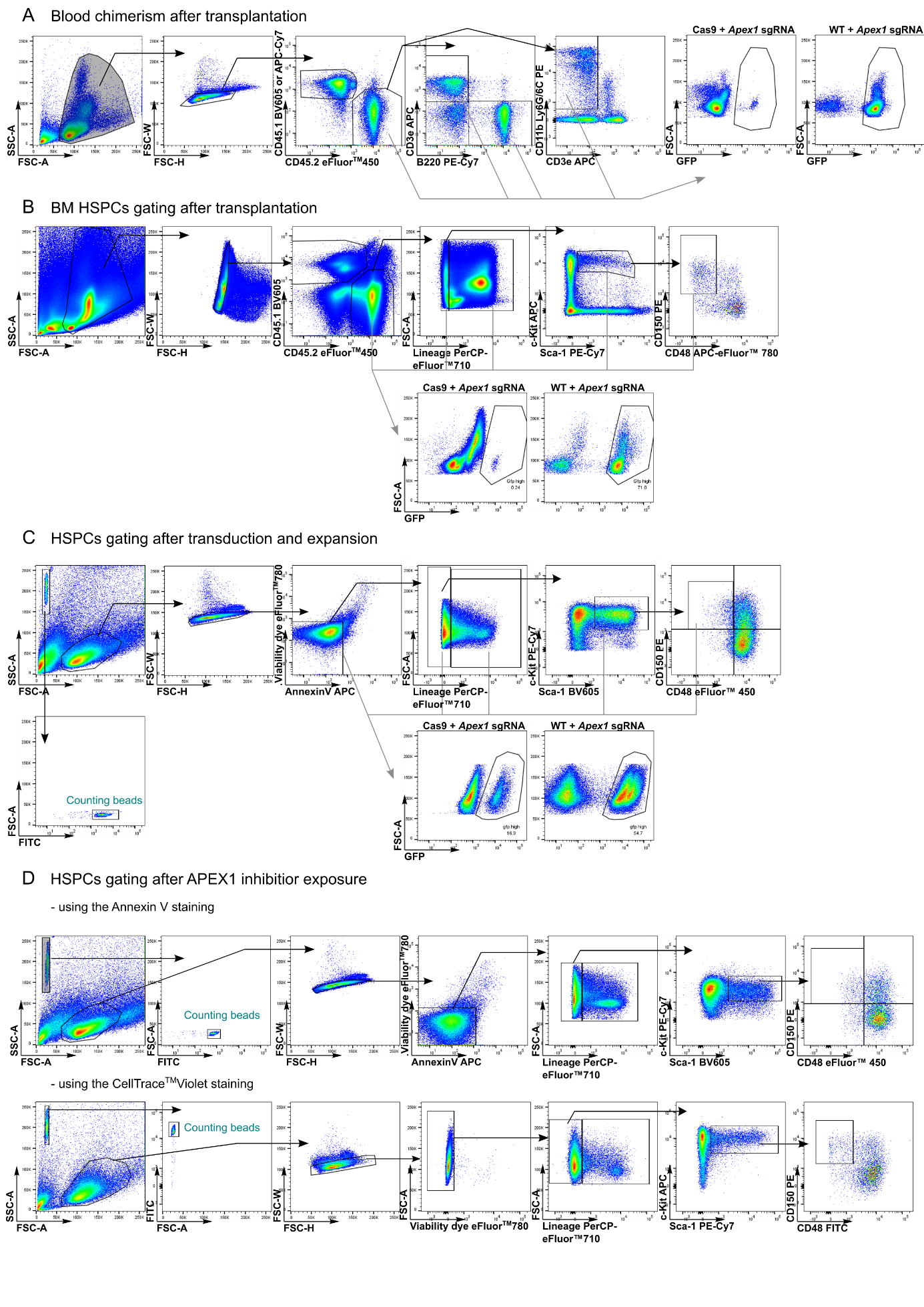
**

**Figure S16: Flow cytometry gating strategies**. (A) Blood repopulation percentage of the transduced cells in the total, B, T, and myeloid lineages (same approach was done for the lineage committed cell analysis in the BM). (B) BM repopulation of the transduced cells in the Lin^-^, LSK and LSK-SLAM populations. (C) Expansion of the transduced Lin^+^, Lin^-^, LSK and LSK-SLAM cells. (D) Expansion of the Lin^+^, Lin^-^, LSK and LSK-SLAM cells after APEX1 inhibitor or DMSO treatment.


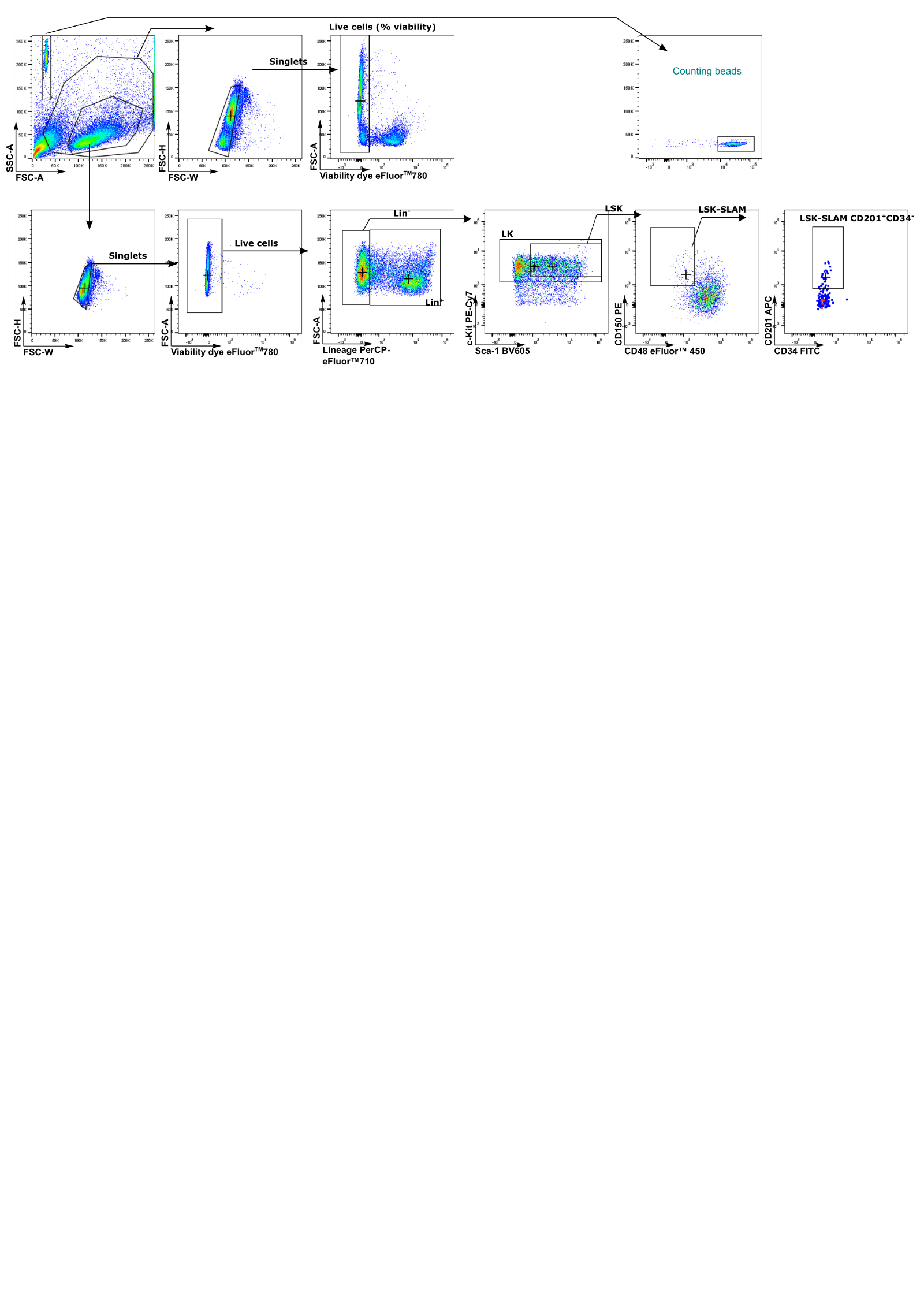


**Figure S17: Flow cytometry gating strategy upon IFN treatment.**


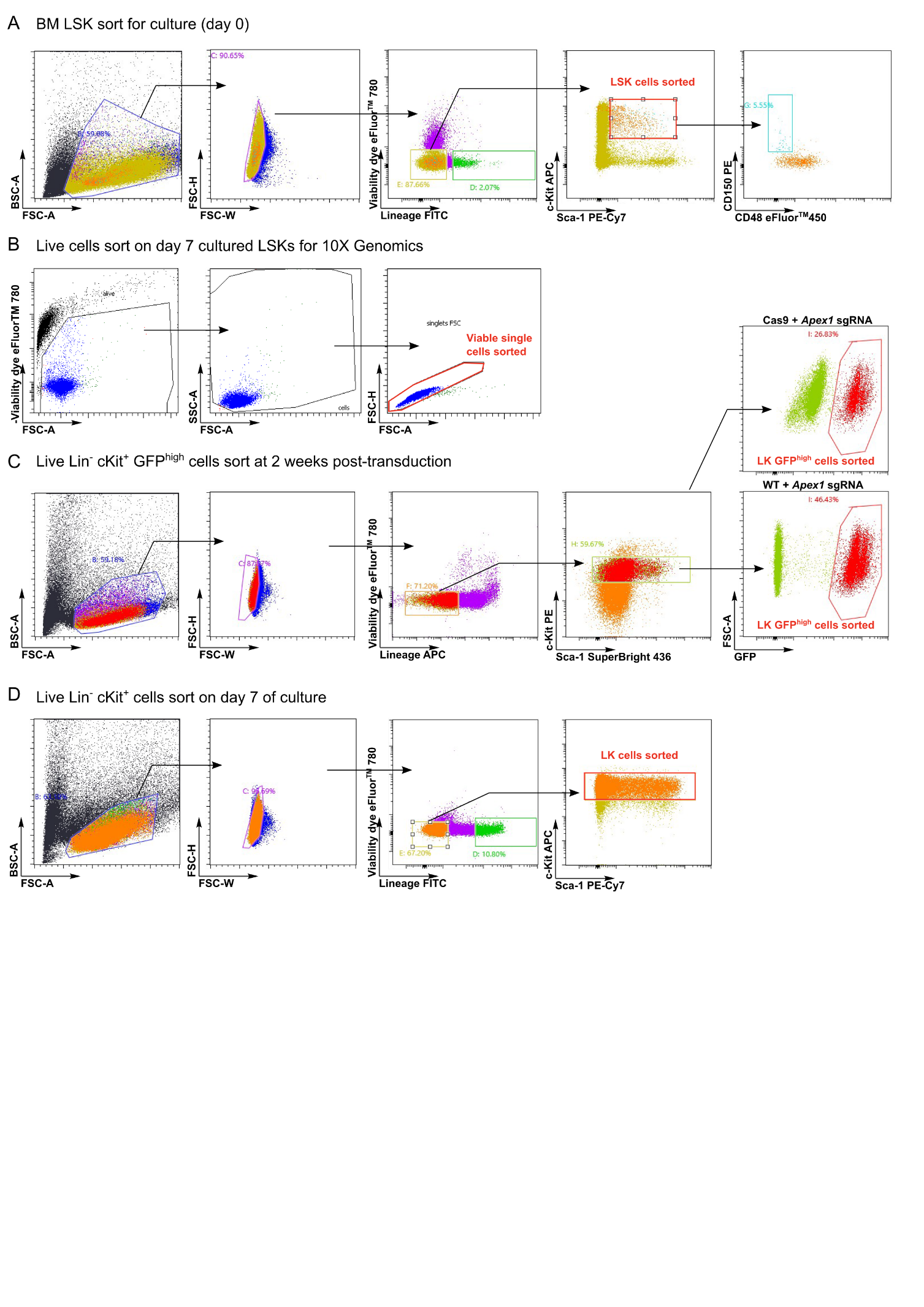


**Figure S18: FACS prolifes used for cell sorting.** (A) Freshly isolated BM LSK sort for culture (day0). (B) Viability sort on the LSK progeny after 7 days of culture for 10X Genomics experiment. (C) Lin^-^ cKit^+^ (LK) GFP^high^ sort from the transduced *Apex1* sgRNA Cas9 and WT cells, at 2 weeks post-transduction. (D) Lin^-^cKit^+^ (LK) sort after 7 days of APEX1 inhibitor or DMSO exposure.
